# Population genetics of the Mediterranean corn borer (*Sesamia nonagrioides*) differs between wild and cultivated plants

**DOI:** 10.1371/journal.pone.0230434

**Published:** 2020-03-19

**Authors:** Abdel Kader Naino Jika, B. Le Ru, C. Capdevielle-Dulac, F. Chardonnet, J. F. Silvain, L. Kaiser, S. Dupas

**Affiliations:** 1 Laboratoire Evolution, Génomes, Comportement et Ecologie, UMR CNRS, IRD & Université Paris-Sud Orsay, Gif-sur-Yvette cedex, France; 2 ICIPE- African Insect Science for Food and Health, Kasarani, Nairobi, Kenya; National Cheng Kung University, TAIWAN

## Abstract

The population genetic structure of crop pest populations gives information about their spatial ecology, which helps in designing management strategies. In this paper, we investigated the genetic structure of the Mediterranean Corn Borer (MCB), *Sesamia nonagrioides* Lefèbvre (Lepidoptera: Noctuidae), one of the most important maize pests in the Mediterranean countries, using microsatellite markers for the first time in this species. Insects were collected in twenty-five locations in southwest and southeast France from cultivated and wild host plants (*Zea mays*, *Sorghum halepense and Typha domingensis*). Contrary to what has been reported so far in France, we found that *MCB* populations could be locally abundant on wild poales plants. Analysis was carried out at 11 polymorphic microsatellite markers. Molecular variance was significantly determined by geography, then by host plant, with 17% and 4%, respectively, when considered as a major effect, and with 14% and 1%, respectively, when considered as a marginal effect in permutational analysis. Multidimensional scaling (MDS) and GENELAND Bayesian clustering suggested that populations infecting wild plants (*T*. *domingensis* and *S*. *halepense*) were more structured locally than those affecting cultivated maize. In *S*. *halepense*, significant Isolation By Distance (IBD) indicated that this factor could explain genetic differentiation of the moth populations. In *T*. *domingensis*, local population differentiation was strong but did not depend on distance. The implication of this absence of population structure in maize and the heterogeneity of population genetics patterns in wild plants are discussed in the context of the population dynamics hypothesis and population management strategies.

## Introduction

*Sesamia nonagrioides* Lefèbvre (Lepidoptera: Noctuidae), also known as the Mediterranean Corn Borer (MCB), corn stalk borer or pink stem borer, is one of the most damaging pests of maize (*Zea mays* L.) in several southern European countries. With *Ostrinia nubilalis* Huebner (Lepidoptera: Crambidae), which has an overlapping but more northerly distribution, they can damage up to 80% of maize plants [1]. In France, MCB populations are established on maize south of 45° north latitudes [2], in low altitude and humid areas (Aquitaine and the southern Rhone Valley). Their occurrence in maize is now tending to expand north along the Atlantic coast [3]. The pest has recently become a serious sanitary problem in maize, because there is no possible treatment against the second generation of larvae, which live in full-grown plants. A *Fusarium* fungus, which is toxic for the cattle, often develops in larval tunnels [4]. The number of generations in this species is governed by the onset of diapause and appears to be correlated with photoperiod and temperature [5]. The number ranges from two generations in France (May-June, July-August) [2], to five generations in Iran (four during the active season, with a partial fifth generation in second plantings) [6]. Very little is known about MCB occurrence on wild host plants in Europe and the Middle East or its role in population spatial dynamics across seasons, while the potential role of wild habitats as a reservoir remains poorly documented.

Phylogeographic analyses using one nuclear and two mitochondrial genes [7] suggested that European populations originated from a single colonization event from Africa. This colonization process is thought to have occurred about 100,000 years ago, either across the Strait of Gibraltar or across the Sahara and Sinai, which were vegetated places between the last two ice ages. Buès *et al*. (1996), who analyzed European and Moroccan populations using allozyme polymorphism, suggested the existence of two population groups, one ranging from northwest Spain to southwest France and the second from Morocco and northeast Spain to southeast France [8]. In Europe, with a more easterly sampling, Margaritopoulos *et al*. (2007), using amplified fragment length polymorphism (AFLP), observed another population group in the south of Greece that was separated from the population grouping in Spain, southwest France, Italy, and Northern Greece [9]. Finally, De la Poza *et al*. (2008), on the basis of random amplified polymorphic DNA (RAPD), suggested that populations from Spain and the Southwest of France were separated from populations from Italy, Greece and Turkey [10]. It is still difficult to draw a general populations structure from these studies since they deal with different regions and different markers, but they generally suggest the presence in Europe of one continental population, and several more southern populations in the European peninsula.

Although the MCB is known to feed on a diversity of wild and cultivated *Poales* [11], studies considering plant host species as a factor of the moth population genetic structure are still limited. Such genetic differentiation between wild and cultivated host plant populations have been demonstrated in the Lepidoptera species ([12–14] for *O*. *nubilalis*) and noctuids ([15, 16] for *Spodoptera frugiperda*). It was also suggested for *Sesamia calamistis* [17]. The question is of crucial interest for the MCB, which is assumed to have colonized Europe long before the advent of agriculture and probably co-evolved with wild host plants that have been in this region since then [7]. A pioneer study was carried out by Leniaud *et al*. (2006) on caterpillars collected from maize (*Z*. *mays*), cultivated sorghum (*Sorghum* sp), sunflower (*Helianthus annuus* L.), pepper (*Capsicum frutescens* L.) and cantaloupe (*Cucumis melo* L.) in southern France regions [18]. Using allozyme polymorphism, the authors found that host-plant diversity, rather than spatial distance, was clearly associated with genetic differentiation between populations. They interpreted their results as a consequence of different selective pressures exerted repeatedly on the various hosts and/or fixed genetic differences due to assortative mating, i.e. the MCB host races. Additional studies are therefore needed to better assess host plant and geographic determinants of the fine genetic structure of the MCB.

In this paper, we used microsatellite markers for the first time and sampled cultivated and non-cultivated host plants in southern France to assess the genetic structure of the MCB. Microsatellite markers are co-dominant, highly polymorphic and more likely to be neutral [19] as compared to previously used markers that lack at least one of these characteristics (allozymes, RFLP, AFLP, RAPD). The main objective of this study was to assess the relative role of the host plant and geography in the local genetic structure of the MCB in the south of France and to discuss the population dynamics of this species in the light of available data. We sampled MCB populations on Maize, *Sorghum halepense* and *Typha domingensis* across southern France. We jointly analysed geographic and ecological factors for genetic differentiation through multidimensional descriptive statistics and permutation tests for major and marginal effects.

## Material and methods

### Insect sampling

**Fig 1:**
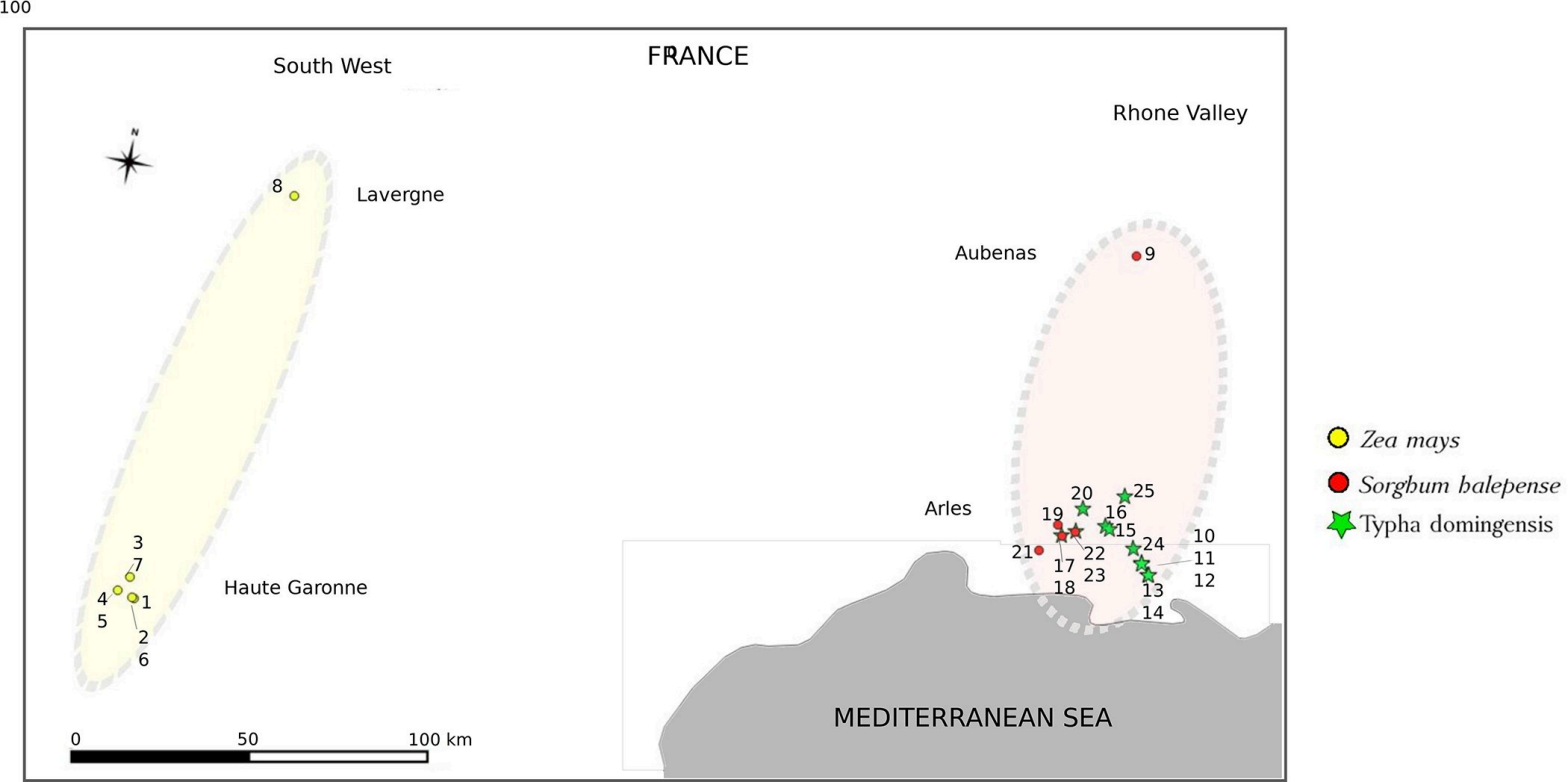
Geographical location of *S*. *nonagrioides* (MCB) samples. The ellipses represent the two regions studied (yellow: southwest and brown: Rhone Valley); the colored squares distinguish the county. Yellow and red circles represent MCB samples collected from *Zea mays* and *Sorghum halepense*, respectively; green stars represent individuals collected from *Typha domingensis*. A superposition of green star and red circle corresponds to the localities in which insects were collected on both *S*. *halepense*, and *T*. *domingensis*.

Larvae were collected from both wild and cultivated host plants in twenty-five locations in two regions of southern France (Fig 1). In the southwest region, sampling was done in one maize field in the Lot county (Lavergne) in December 2008 and in seven maize fields in the Haute Garonne county during March 2012 and 2014 (Table 1). In this area of intensive maize growing (e.g. in Longages in Haute Garonne, 33% of the utilized agricultural land is devoted to maize, Arvalis, 2010 unpublished data), the wild plant hosts for the MCB are scarce, and we failed to find the moth in them. In the Rhone Valley region, larvae were sampled from two wild host plants, *Typha domingensis* (the MCB major wild host plant in its native African region) and *Sorghum halepense*, in 14 collection points in the Bouche du Rhône county (Camargue, south of Arles) and in one collection point in the Rhone Valley area in the Ardèche county (Aubenas) in July 2012 (S1 Table). In this area maize is scarce (Arles, 0.15% of the utilized agricultural land is devoted to maize, Arvalis, 2010 unpublished data), making insect collection on this crop difficult. Thereafter, the term “population” refers to MCB *individuals* collected from the same host plant in the same location (field or collection point) on the same date. Even if we could not find places hosting MCB on both wild and cultivated plants, having samples on the same plant from different localities helped to determine the effect due to distance, and having some samples from the same locality on different wild plants helped to determine the effect from plants. A total of 451 caterpillars were sampled, and each individual was preserved in ethanol (95%) before DNA extraction.

**Table 1:** *S*. *nonagrioides* population sample characteristics and sample location coordinates. *Z*. *mays*: cultivated plant, *T*. *domingensis* and *S*. *halepense*: wild plant. The number in brackets in the locality column is the county number (Lot: 46; Haute-Garonne: 31; Bouches-du-Rhône: 13; Ardèche: 07).

Population	Locality	Host Plant	Date	Latitude	Longitude	Size
1	Longage 1 (31)	*Z*. *mays*	March-12	43.3695	1.1902	31
2	Longage 2 (31)	*Z*. *mays*	March -12	43.3746	1.2007	31
3	St-Clar-de-Rivière (31)	*Z*. *mays*	March -12	43.448	1.1937	29
4	Longages 3 (31)	*Z*. *mays*	March -14	43.3680	1.1926	23
5	Longages 4 (31)	*Z*. *mays*	March -14	43.3699	1.199	23
6	Poucharramet (31)	*Z*. *mays*	March -14	43.4205	1.1753	24
7	Cambernard (31)	*Z*. *mays*	March -14	43.4713	1.1792	21
8*	Lavergne (46)	*Z*. *mays*	Dec-08	44.78	1.75	2
9*	Aubenas (07)	S. *halepense*	July-12	44.569	4.698	4
10*	Arles 2 (13)	*T*. *domingensis*	July-12	43.493	4.714	4
11	Arles 3 (13)	*T*. *domingensis*	July -12	43.493	4.714	33
12	Arles 4 (13)	*T*. *domingensis*	July -12	43.492	4.715	29
13*	Arles 6 (13)	*T*. *domingensis*	July -12	43.453	4.741	1
14	Arles 7 (13)	*T*. *domingensis*	July -12	43.452	4.737	19
15	Arles 8 (13)	*T*. *domingensis*	July -12	43.611	4.605	8
16*	Arles 9 (13)	*T*. *domingensis*	July -12	43.622	4.589	1
17	Arles 12 (13)	S. *halepense*	July -12	43.59	4.438	48
18	Arles 12 (13)	*T*. *domingensis*	July -12	43.59	4.438	40
19*	Arles 13 (13)	S. *halepense*	July -12	43.62977	4.42562	2
20*	Arles 14 (13)	*T*. *domingensis*	July -12	43.684	4.513	4
21	Arles 15 (13)	S. *halepense*	July -12	43.541	4.355	31
22*	Arles 16 (13)	*T*. *domingensis*	July -12	43.605	4.486	1
23	Arles 16 (13)	S. *halepense*	July -12	43.605	4.486	26
24	Arles 17 (13)	*T*. *domingensis*	July -12	43.544	4.686	7
25	Arles 18 (13)	*T*. *domingensis*	July -12	43.727	4.658	9

*: These populations are not real populations given the small size, but are individuals collected from the same host plant in the same location at the same date

### Molecular analysis

The DNA extractions were performed on third-stage larvae (whole body) using the Qiagen ® Dneasy Blood and Tissue Kit. The microsatellite loci used were developed by Capdevielle *et al*. in 2012 (unpublished data) using next generation sequencing, confirmed by amplification, which allowed us to identify 17 suitable markers (SN01; SN16; SN20; SN44; SN15; SN22; SN37; SN18; SN25; SN34; SN42; SN21; SN32; SN45; SN68; SN59; SN61). In order to develop efficient and reliable multiplex PCR amplifications, these 17 microsatellites markers were tested in combinations of three to four loci per amplification reaction. Five multiplexes that showed clear and unambiguous amplification profiles were selected: **multiplex 1**: SN01; SN16; SN20; SN44; **multiplex 2**:SN15; SN22; SN37; **multiplex 3:** SN18; SN25; SN34; SN42; **multiplex 4**: SN21; SN32; SN45; **multiplex 5**: SN68; SN59; SN61. The final PCR consisted of 5μl Platinum® Multiplex PCR Master Mix (Applied Biosystems®), 1μl Primer Mix, 3μl H_2_O and 1μl DNA.

Amplifications were performed in 96-well thermocycler using the following program: 5 mn at 95° C to activate the taq polymerase, followed by 25 cycles of 30 s denaturation at 95° C, 30 s for primer annealing at 55° C and 30 s extension at 72° C, and final extension of 5 mn at 72° C. The amplified fragments were detected by a capillary sequencer ABI 3130xl (Applied Biosystems ®). Microsatellite profiles and allele scoring were made using GeneMapper Software (Version 4.0). The genotype scoring was manually checked for every individual. Two loci having too much missing data (SN32 and SN61) and two others, which were not polymorphic at all in our samples (SN25 and SN68), were removed. The remaining 13 microsatellite loci were analysed separately for conformity with Hardy–Weinberg (HW) equilibrium expectations using Genepop v4.0.1 [20].

Lepidoptera are known to have a high frequency of null alleles [21,22]. These alleles are due to nucleotide variation in the flanking region and make a locus appear homozygous, or result in no amplification at all if both alleles at the locus are null. Because the presence of null alleles may overestimate F-statistics [23], we checked their presence and estimated their proportions using INEst software [24]. Six loci were suspected to present null alleles (SN 01; SN16; SN 21; SN22; SN 37 and SN 44). The bias introduced by null alleles on F-statistics are considered significant when their frequencies are superior to 0.2 [23,25,26]. We therefore removed all suspicious loci having frequencies higher than 0.2 (SN 16 and SN59), which lead to the 11 microsatellites markers used in this study.

### Statistical analysis

#### Genetic diversity

Population genetic diversity was assessed for populations represented by at least 5 individuals, by estimating allelic richness (*A*_*r*_), using Fstat v2.9.3.2 software [27], and the number of alleles per locus, the observed (*H*_*o*_) and unbiased expected heterozygosity (*H*_*e*_) for each locus in each population, using Genetix v4.05 software [28]. We used Genetix and FreeNA software to estimate *F*_*IS*_ values for each population and pairwise F_*ST*_ values between all pairs of populations, between regions, county, localities or plants, and overall F_*ST*_ values. *F*_*IS*_ 95% confidence interval (95% CI) and F_*ST*_ significance were computed on the basis of 1,000 bootstraps over loci.

#### MCB genetic structure

A neighbor-joining tree was built on the basis of Cavalli-Sforza and Edward (1967) distances using POPULATIONS v 1.2.32 [29]. The reliability of each node was estimated by 1,000 re-samplings of the data over loci. We also carried out non-metric multidimensional scaling (MDS), a non-linear equivalent of Principal Coordinate Analysis, which allowed us to optimize the graphical representation of genetic distance data. The population genetic structure was further characterized using Bayesian assignment approaches implemented in STRUCTURE 2.3.4 [30], TESS 2.3.1 [31] and GENELAND 4.06 [32].

Although TESS and GENELAND can account for geographic information in the definition of clusters, this option was not used. It would have favored spatial factors to the detriment of the ecological factors that we intended to compare in our study. For STRUCTURE and TESS, the “Admixture” model was carried out, allowing K to range from 2 to 10 (5 replicates of 3.10^4^ burn-in iterations followed by 7.10^6^ iterations for STRUCTURE and 7.10^5^ iterations for TESS, for each value of K). For STRUCTURE, we used a model with correlated allele frequencies [33] and the best solution was identified using ΔK statistics [27]. For TESS we performed the Conditional Auto-Regressive (CAR) model with spatial trend (ψ) set to Zero, very close to the algorithm implemented in STRUCTURE [30,33]. Results obtained with the different K values were compared using the deviation information criterion (DIC). In order to identify the existence of distinct solutions across TESS and STRUCTURE replicates, we used CLUMPP v1.1.2 software [34] to compute a symmetrical similarity coefficient between the different replicates (greedy algorithm, 100 random input sequences, G’ statistic). The analysis performed with GENELAND used a non-spatial model and correlated allele frequencies among clusters [33], with a number of iterations of 106, a thinning of 100, and with the possible number of clusters varying from 1 to 20. Burning was set at 2000 (20% of the recorded iterations) after visualisation of posterior trace. Post process chain analysis considered a 100 by 100 grid for mapping.

Finally, we carried out a non Bayesian DAPC clustering based on PCA and discriminant analysis [35] using find.clusters command implemented in adegenet R package.

Graphical displays of the individual assignment probabilities were generated using Distruct v1.1 [36].

#### Correlation between genetic and geographic distances

Isolation by distance (IBD) was tested using individuals as replication unit. It used a linear regression of F_*ST*_ / (1-F_*ST*_) estimated by a_values, by the logarithm of geographical distances, as proposed by Rousset [37] using Genepop v4.0.1 software [20], while statistical relationship was tested using the Spearman rank correlation coefficient (permutations).

#### Plant and locality effects

Genetic diversity indices were used to investigate the differences between insects collected from different host plants or different locations. For that, we used Kruskal-Wallis non-parametric analysis of variance using populations as a repetition unit, followed by *post-hoc* multiple comparison (Wilcoxon rank sum test adjusted by a sequential Bonferroni when significant effects are detected), using R [38]. To distinguish the effect of geography and the host plant in the genetic differentiation among MCB populations, we carried out permutational multivariate analysis of variance (PAMOVA), an extension of molecular analysis of variance (AMOVA) [39] that allowed us to estimate effects in a non-hierarchical way, either as a principal or as a residual effect, and thereby account for correlation among factors [40]. Significance of region, locality and plants, were considered sequentially by permutational analyses using the *adonis* function implemented in the vegan R package [41]. Plant marginal effects were estimated after the locality effect was accounted for, to evaluate the structure due to the plant only. To evaluate the effect of geography on genetic differentiation we performed mantel tests within the different host plant samples in the southwest and in the Rhone Valley separately and among all datasets.

## Results

### Within-population genetic diversity

Allelic richness (*A*_*r*_) and expected heterozygosity (*H*_*e*_) ranged from 1.32 to 1.56 (mean of 1.46) and from 0.32 to 0.56 (mean of 0.46), respectively (Table 1). The number of alleles per locus ranged from 2 (locus 11) to 13 (locus 2) with a mean of 5.8 (S2 Table). The mean deviation from H-W expectations was variable among populations, with *F*_*IS*_ varying from 0.15 to -0.19 (mean of 0.032). These within-population genetic diversity indices were not significantly different among insects collected from the two regions (the southwest and the Rhone Valley; P = 0.6 for He, P = 0.55 for Ho; P = 0.14 for F_*IS*_, P = 0.6 for Ar), from the different populations within regions (P = 0.46 for He, P = 0.46 for Ho; P = 0.46 for F_*IS*_, P = 0.46 for Ar) and from the three different host plants (P = 0.24 for He, P = 0.84 for Ho; P = 0.16 for F_*IS*_, P = 0.24 for Ar) or maize vs wild host plants (P = 0.6 for He, P = 0.55 for Ho; P = 0.14 for F_*IS*_, P = 0.597 for Ar) (Table 2).

**Table 2:** Genetic diversity indices per population (populations with at least 5 individuals); SD: Standard deviation; CI: Confidence interval; *H*_*e*_: Unbiased expected heterozygosity; *H*_*o*_: Observed heterozygosity, F*is*: Inbreeding coefficient and *A*_*r*_ allelic richness.

Population	locality	H*e* (SD)	H*o* (SD)	F_*IS*_ [CI 95%]	A*r*
1	Longage 1 (31)	0.435 (0.23)	0.43 (0.25)	0.001 (- 12−0.092)	1.43
2	Longage 2 (31)	0.48 (0.25)	0.45(0.24)	0.07 (-0.03−0.13)	1.48
3	St-Clar-de-Rivière (31)	0.45 (0.25)	0.41 (0.26)	0.10 (-0.05−0.18)	1.45
4	Longages 3 (31)	0.47 (0.25)	0.42(0.22)	0.12 (-0.005−0.19)	1.47
5	Longages 4 (31)	0.49 (0.25)	0.44 (0.25)	0.09 (-0.03−0.16)	1.49
6	Poucharramet (31)	0.50 (0.23)	0.54 (0.28)	-0.08 (-0.21−0.01)	1.5
7	Cambernard (31)	0.49 (0.22)	0.42 (0.21)	0.15 (0.01−0.22)	1.49
11	Arles 3 (13)	0.44 (0.22)	0.44 (0.29)	-0.002 (-14−0.09)	1.44
12	Arles 4 (13)	0.41 (0.27)	0.49 (0.44)	-0.19 (-0.27−-0.14)	1.41
14	Arles 7 (13)	0.50 (0.23)	0.46 (0.28)	0.07(-0.10−0.18)	1.5
15	Arles 8 (13)	0.50 (0.30)	0.57 (0.39)	-0.15(-0.40−-0.10)	1.5
17	Arles 12 (13)	0.50 (0.19)	0.45 (0.22)	0.09 (0.001−0.15)	1.5
18	Arles 12 (13)	0.51 (0.20)	0.45 (0.21)	0.10(0.02−0.15)	1.51
21	Arles 15 (13)	0.50 (0.22)	0.45 (0.21)	0.11 (0.01−0.17)	1.5
23	Arles 16 (13)	0.50 (0.20)	0.49 (0.21)	0.02 (-0.1−0.01)	1.5
24	Arles 17 (13)	0.45 (0.28)	0.40 (0.31)	0.12 (-0.22−0.25)	1.45
25	Arles 18 (13)	0.49 (0.25)	0.52 (0.28)	-0.07(-0.32−0.04)	1.49

### Genetic clustering

**Fig 2:**
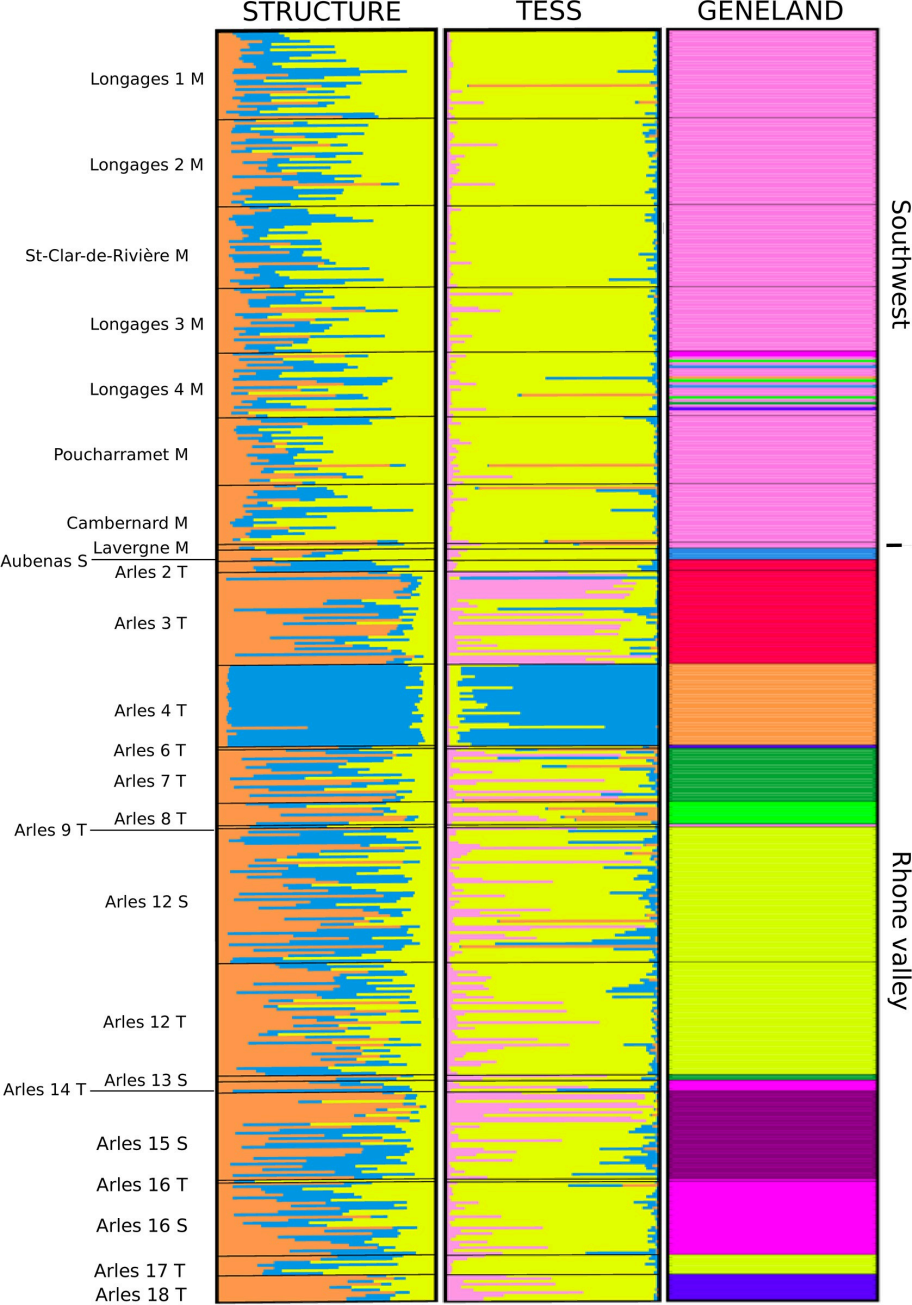
Genetic structure of *S*. *nonagrioides* in southern France provided by Bayesian clustering (structure (K = 3), TESS (K = 4) and Geneland (K = 10). Each thin vertical line corresponds to one individual. Coloured segments represent the proportion of each individual’s genome assigned to each genetic cluster.

The admixture models (TESS and STRUCTURE) gave comparable results and differed from GENELAND (Fig 2). The optimal number of clusters was K = 3 for STRUCTURE, while in the analysis with TESS software, the DIC curve did not strictly reach a plateau (S1 Fig). However, when K was greater than 4, solutions proposed by TESS included empty clusters, so that the observed genetic structure was very similar to the solutions obtained for K = 4. For these reasons, we focus on K = 4 as the TESS best solution; one of the clusters in TESS (K = 4 solution) made only a small and admixed contribution. Among the five repetitions carried out with the optimal number of clusters for both STRUCTURE and TESS, we found very similar results (Fig 2, S2 and S3 Figs). South-western populations (Haute Garonne and Lavergne) in TESS, and the Arles 4 population in both TESS and STRUCTURE were very little admixed and mostly composed of a single cluster. All other populations were constituted with admixed individuals, supporting high levels of ongoing gene flow among all analysed populations (Fig 2). Results revealed in both analyses that the Arles 4 population was clearly different from all other populations, even from the *MCB* specimens collected from very close localities (Fig 2). The results of GENELAND (non-admixture and non-spatial model) were different. Strong local clustering with 10 Clusters corresponding to local populations were observed (Fig 2). Individuals from different localities, even when very close, were generally assigned to different clusters, and individuals from the same locality were generally assigned to the same single cluster (except for Longages 4). The *Fis* in the 10 clusters ranged from -0.18 to 0.15 with an average of -0.01, suggesting that the defined clusters were close to Hardy Weinberg equilibrium. On the other hand, using the model-free DAPC, the BIC exhibits minimum values for number of clusters between 20 and 29 (S4 Fig) with no geographic signal (S5 Fig).

### Host plant and geographically associated genetic structure

**Fig 3:**
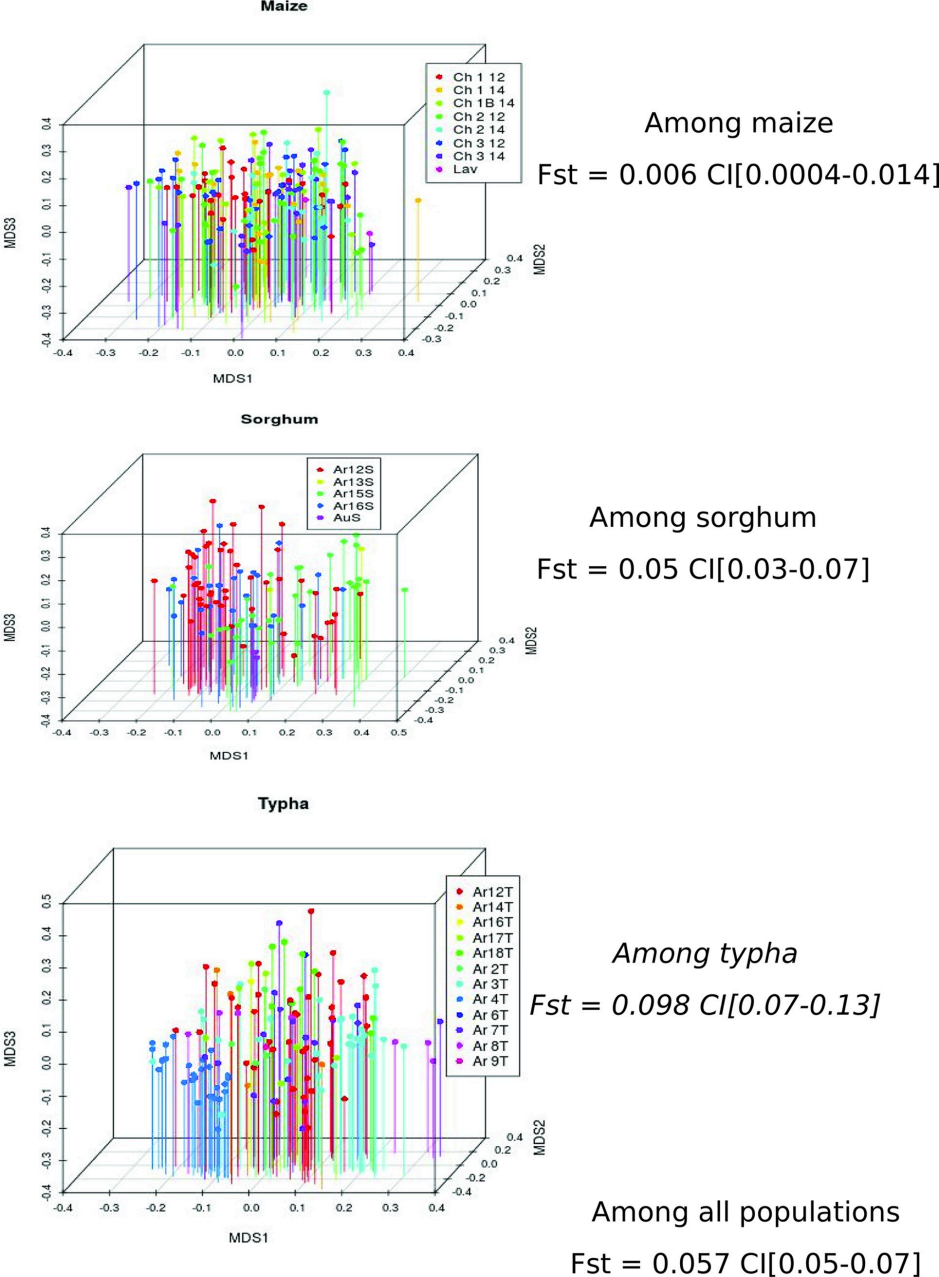
Multidimensional scaling representation of genetic distance among local populations in maize, *Sorghum halepense* and *Typha domingensis*, and F_*ST*_ values among individuals in each host plant and among all populations.

PAMOVA analysis was performed, considering the different factors (region, county, locality or plant) alone, as marginal effect, or within sub-samples for each host plant (i.e. individuals collected on the same host plant whatever the collection locality) (Table 3). We observed that all factors were significant when considered either alone (P < 10^−5^, F_*ST*_ = 0.057 among host plants, Fig 3) or as a marginal effect nested within their hierarchical superior (county within regions, P < 10^−5^; locality within county, P < 10^−4^), plants within localities (P = 0.0003) and reciprocally localities within plants (P < 0.0001). When considering genetic differentiation between localities within sub-samples of each host plant, geographic differentiation was significant for *T*. *domingensis* and *S*. *halepense* (P < 0.0001), but was not significant for maize, although the mean geographic distance between *T*. *domingensis* localities was smaller (16.2 km on average) than between *S*. *halepense* or maize localities (49.1 and 44.2 km on average, respectively). This difference in geographic structure according to the plant species can be visualized by MDS representations (Fig 3), where clustering of samples within locality can be seen for *T*. *domingensis* and *S*. *halepense* samples but not for maize samples. This indicates that individuals collected on wild host plants (*T*. *domingensis* and *S*. *halepense*) have more geographic structure compared to individuals collected on maize, suggesting that host plant species may influence the moth’s propensity to disperse (Fig 3).

**Table 3:** Permutational analysis of molecular variance (PAMOVA), using Cavalli-Sforza and Edward distances matrix with 10^4^ permutations. a) Analysis with one factor b) marginal effect c) analysis in one host plant species only.

	Factor	df	Var. prop.	F	P-value	F_*ST*_
**a)**	Region	1	0.034	15.946	1,00E-05	0.007
** **	County	3	0.045	7.044	1,00E-05	0.003
** **	Locality	21	0.172	4.242	1,00E-05	0.031
** **	Plant	2	0.042	9.789	1,00E-05	0.011
**b)**	County within region	2	0.034	16.055	1,00E-05	
** **	Locality within county	18	0.045	7.796	1,00E-04	
** **	Locality within plant	20	0.141	3.552	1,00E-05	
** **	Plant within locality	1	0.007	3.291	0.00021	
**c)**	Localities within *Typha*	10	0.27	5.36	1,00E-05	
** **	Localities within *Sorghum*	4	0.105	3.103	1,00E-05	
** **	Localities within maize	7	0.049	1.298	0.05749	

IDB patterns back up this result. To account for the difference in sample size among populations, we performed Mantel tests at the individual level (Rousset 2000). Results are shown in Table 4. Mantel tests on the whole data set showed that geographic distance only slightly explained the observed genetic differentiation (slope = 0.005; max. distance between populations ≈ 280 km). The genetic diversity did not show any significant IBD pattern in Southwest (mays) either in Rhone Valley (South West: P = 0.170; Rhone Valley: P = 0.220), likely reflecting very high levels of effective gene flow. However, when considering separate host plants, we observed significant IBD pattern in Rhone Valley (P = 0.0006) on samples from *S*. *halepense* but not on samples from *T*. *domingensis* (P = 0.23). Yet in *T*. *domingensis*, genetic differentiation was strong, even at a short distance (F_*ST*_ = 0.181 between Arles 3 and Arles 4 at a distance of 0.137 km) (S3 Table). This is illustrated by the important intercept of IBD regression in this host plant (Table 4). By contrast, F_*ST*_ value, on samples from S. halepense in Rhone Valley, were small at short distance (F_*ST*_ = 0.02–0.03 in Arles at a distance of 4.5–13 km), and increased to 0.147–0.30 between Arles and Aubenas at a distance of 99–113 km. This is illustrated by the smaller intercept and the larger slope in this host species (Table 4). In maize the differentiation was low at low distance and did not increase with distance.

**Table 4:** Mantel test of geographic differentiation between individuals (Rousset 2000). P-values were calculated over 10,000 permutations.

Sub-sample	Slope	P-value	CI Slope	Intercept	CI Intercept
All Data	0.005	1.71E-08	[0.003–0.009 ]	0.11	[0.052–0.1765 ]
Southwest (mays)	0.006	0.17	[-0.006–0.02 ]	0.09	[0.017–0.16 ]
Rhone Valley	0.004	0.22	[-0.005–0.02 ]	0.40	[0.211–0.82 ]
*Typha domingensis*	0.008	0.23	[-0.01–0.03 ]	0.39	[0.182–0.83 ]
*Sorghum halepense*	0.18	0.0006	[0.06–0.34 ]	0.91	[0.498–1.645 ]

## Discussion

### Existence of host races

The existence of host races, i.e. genetically differentiated populations specialized on different hosts, has been demonstrated in several Lepidoptera species [42] and in particular for *Ostrinia nubilalis* [12,14,18,43]. In our study, genetic differentiation due to host plant, was limited but significant (F_*ST*_ = 0.01, 4% of variance explained). It was still important in sympatry between *S*. *halepense* and *T*. *domingensis* (F_*ST*_ = 0.02 at Arles 12 and F_*ST*_ = 0.05 at Arles 16). It was observed in the AMOVA as a nested marginal effect within locality. Similar genetic differences between plants were observed by Leniaud (2006) on the basis of only 3 polymorphic allozyme markers between MCB populations collected from maize and *Sorghum* (F_*ST*_ = 0.01) in the South of France. As a comparison, the level of differentiation between mono- and dicotyledonous host plant variants of the European Corn Borer (ECB), now considered as different species (*Ostrinia nubilalis and O*. *scapulalis*, respectively) [13] ranges from F_*ST*_ = 0.016 [18] to F_*ST*_ = 0.026 [12] for allozyme markers and F_*ST*_ between 0.032 and 0.053 for AFLP markers [13]. The lower differentiation between MCB than ECB plant variants in Europe, is illustrated by STRUCTURE Bayesian clustering analyses, which did separate *Ostrinia* species [13] but did not separate MCB variants in our study. Ultimately, ECB host populations have been considered as different species on the basis of differences in timing of moth emergence and sex pheromone composition [44]. In Africa there are MCB sister species, with different levels of specialization toward the host plant or habitat [11]. The general picture is therefore a generalist species attacking maize and more specific wild relatives. In Europe, repetitive sampling of MCB across year would be necessary to evaluate the stability of the plant differentiation. Note that *S*. *nonagrioides* on maize lays eggs beneath windings of young leaf sheets, and in our rearing units, specifically on paper sheet rolls serving as structural surrogates, that are absent in dicotyledons [45,46]. The MCB sampled by Leniaud [18] on dicotyledons in Europe may therefore express a different oviposition behavior with a probable genetic basis, as opposed to the monocotyledon populations of the present study. Alternatively, *S*. *nonagrioides* oviposition and larval feeding preference traits may be transmitted between stages and generations not genetically, but by learning plant chemical cues transferred across stages and generation [47]. This fact has received a lot of attention in phytophagous insects because of its possible involvement in ecological speciation and its implication for the management of crop pests [48,49]; it has been documented in Lepidoptera [50,51]. In their Lepidoptera meta-analysis, Petit et *al*. (2015) reported that the most efficient transmission of preference was obtained in studies where the trait was adult oviposition preference and the exposure to the chemical cue at the larval stage [52]. This transmission of preference from larval feeding to adult oviposition traits was also observed in MCB [52]. Such traits can lead to the host-related genetic structure we observed in this study, without the need to invoke genetic based preference, at least among monocotyledonous host species. Therefore, our study does not demonstrate the presence of host races in the MCB, but of more or less sedentary populations on wild plant settlements, possibly explained by the known biology of *S*. *nonagrioides* regarding transmission of preferences among development stages.

### Contrasted and complementary results between Bayesian clustering methods

Our study showed contrasted results between GENELAND non-admixed and TESS-STRUCTURE admixed models. The GENELAND clusters in South East of France were exactly corresponding to local populations. The TESS and STRUCTURE clusters were more homogenous geographically. Only the Arles 4 population formed a separate cluster in the admixed analyses, whereas most of the locations on wild host plants formed different clusters in the GENELAND non-admixed analysis. Safner *et al*. showed by simulation that GENELAND is better at detecting permeable barriers than TESS and other clustering methods [53]. Kalinowski also suggested that STRUCTURE does not always detect within a species genetic structure [54]. Our study confirmed the results of Safner *et al*. (2011) and Kalinowski (2011). GENELAND captured a local structure that was not captured by TESS and STRUCTURE. This local structure is confirmed by the F_*ST*_ values observed at short distance between populations. The two approaches provide complementary information on the population structure of the MCB and seem to work at different scales. We next discuss how the other statistics performed in this paper cast light on the reason for these differences and thereafter, the population biology of MCB.

### Existence and nature of genetic structure depend on the host plant

Many authors [55,56] have considered *S*. *nonagrioides* as a sedentary species. In our study, significant genetic structure was observed among sampling sites (F_*ST*_ = 0.04, 17% of variance explained in PAMOVA). Mantel testing of genetic versus geographic distance was significant overall with extremely low slope indicating that distance plays a weak role in genetic differentiation (Table 4). Yet a neighbor-joining tree (S6 Fig) and the model free DAPC (S5 Fig) did not show geographic signal while Bayesian clusters assuming an admixed model showed very little geographic signal. Other studies, focussing on maize only, failed to find any genetic differentiations among MCB populations from localities sometimes very far away. Indeed, Margaritopoulos *et al*. (2007), using AFLP markers, did not find genetic differences between populations collected at sites 1,800 km apart, from Axioupoli (Greece) to Toulouse (France), and De La Poza *et al*. (2008) did not find significant IBD across the MCB collected in Europe, using RAPD markers [9, 10]. Similarly, Buès *et al*. (1996) found weak differentiations at 12 allozyme loci among MCB populations (F_*ST*_ = 0.064) collected across Morocco, northern Spain, and southern France [8]. These authors put forward the hypothesis that insects could fly over long distances, not across the Pyrenees, but via the Atlantic or Mediterranean coasts, thus creating gene flow between populations. Jones *et al*. (2016) recorded flight performances of tethered insects in a windmill and compared 24 noctuid species. They found total flown distances overnight to vary from 597 m to 12,352 m with a mean value of 4,566 m [57]. Even if this method certainly overestimates flown distances in field conditions, where flight may not be linear, it suggests that the MCB individuals could disperse over less than 12 kilometres during their lifespan [58], so long distance flight would be achieved upon several generations. Considering, for the first time, wild and cultivated plants in our analysis throws light on the debate on the dispersal behavior of the MCB: the GENELAND non-admixed model and MDS representation suggest the pattern of IBD depends on the host plant. GENELAND Bayesian clusters were highly localized on *S*. *halepense* and *T*. *domingensis* and highly regional on maize (one cluster over a maximum distance of 160 km). MDS representation also shows stronger geographic structure on *T*. *domingensis* and *S*. *halepense* than on maize. Lack of geographic distance effect on maize MCB differentiation and possible effect on wild plant MCB is congruent with Leniaud (2006), who sampled MCB on different host plants in southern France and found significant IBD within non maize plant groups and non-significant IBD within the maize group [18].

Our results and other direct experiments therefore strongly support the strong dispersal abilities of maize MCB contrary to what is often alleged so far.

### Implication for MCB management

Understanding the relationships between crop pest populations living in cultivated and uncultivated areas is essential for effective control at a landscape scale. To our knowledge, this is the first MCB population genetic study including non-cultivated plants. This moth is a serious maize pest in southern France, and one question linked to controlling it is its ability to use wild plants as an alternative host. In Africa, the MCB develops populations in many wild plants, including a *Typha* species [59]. In southwest France, a major maize cultivation area, the MCB has been quite rarely found on wild plants after successive searches since 2011 (Kaiser, unpublished). In Camargue (southern Rhone Valley), where maize fields are scarce, we observed it on *S*. *halepense* or *T*. *domingensis* wild host plants (Naino Jika and Le Ru, unpublished data). This suggests that populations may specialize locally on the most abundant resource. Overall, the result suggests relatively varied and separated population dynamics on the three studies host plants. Our hypothesis is that for maize populations, population sizes are large but crop rotation imposes insect dispersion, thus increasing the total effective number of migrants (*Ne×m*), the critical parameter in reducing population structure. In Camargue, *S*. *halepense* and *T*. *domingensis* represent more perennial settlements than maize, since they develop from rhizome in humid non-cropping areas and are both resistant to flooding and drying events. This situation may have allowed the establishment of a pattern of IBD as observed for the moth populations on *S*. *halepense*. In *T*. *domingensis* on the other hand, the moth population genetic structure is strong but does not depend on distance. More precise data on the evolution of both plants and moth populations around the year are needed to understand the factors of the observed geographic structure. The hypothesis that the presence of wild relatives does not increase or could even decrease population dynamics on maize through maladaptation remains to be confirmed. Sampling of the MCB in uncultivated areas must therefore be carried out all across Europe, especially because the biotope favorable to its development, i.e. river banks, ponds, wetlands, estuaries as occurring in the Rhone Valley, in Africa [59] or in Iran [56,60] exists all over Europe.

## Supporting information

S1 Fig A-delta K value as a function of k (STRUCTURE). B- Value of the deviation information criterion as a function of K (TESS). K is the number of inferred genetic clusters.
(TIFF)

S2 Fig Five repetitions of STRUCTURE optimal solution (K = 3). (TIFF)

S3 Fig Five repetitions of TESS optimal solution (K = 4). (TIFF)

S4 Fig Bayesian information criterion curve as a function of K, the number of inferred clusters. (TIF)

S5 Fig Genetic structure of *S*. *nonagrioides* in southern France provided by discriminant analysis of principal components (DAPC) (K = 20). Each thin vertical line corresponds to one individual. Coloured segments represent the proportion of each individual’s genome assigned to each genetic cluster.
(TIF)

S6 Fig Neighbour-joining tree between populations. (TIF)

S1 Table Microsatelite data. Ind: individual ID, Pop: Putative population of individuals, from Column 4, Microsatellite markers used.
(DOCX)

S2 Table Number of alleles per locus and per population. Pop: Putative population, Loc: microsatellite loci.
(DOCX)

S3 Table Pairwise Fst using ENA (Weir 1996). Loc.: Locality, Lo: Longages, Pou: Poucharamet, Cam: Cambernard, Lav: Lavergne, Ar: Arles. Dept.: Department, 07: Ardèche, 13: Bouches du Rhône, 46: Lot, 31: Haute Garonne. Reg.: Region. SW: Southwest, RV: Rhone Valley #: Population number.
(DOCX)
