# Supplementary figures and images for: Population genetics of the Mediterranean corn borer (Sesamia nonagrioides) differs between wild and cultivated plants
Source: PLoS One. 2020 Mar 19;15(3):e0230434. doi: 10.1371/journal.pone.0230434 (PMC7081988; doi:10.1371/journal.pone.0230434)

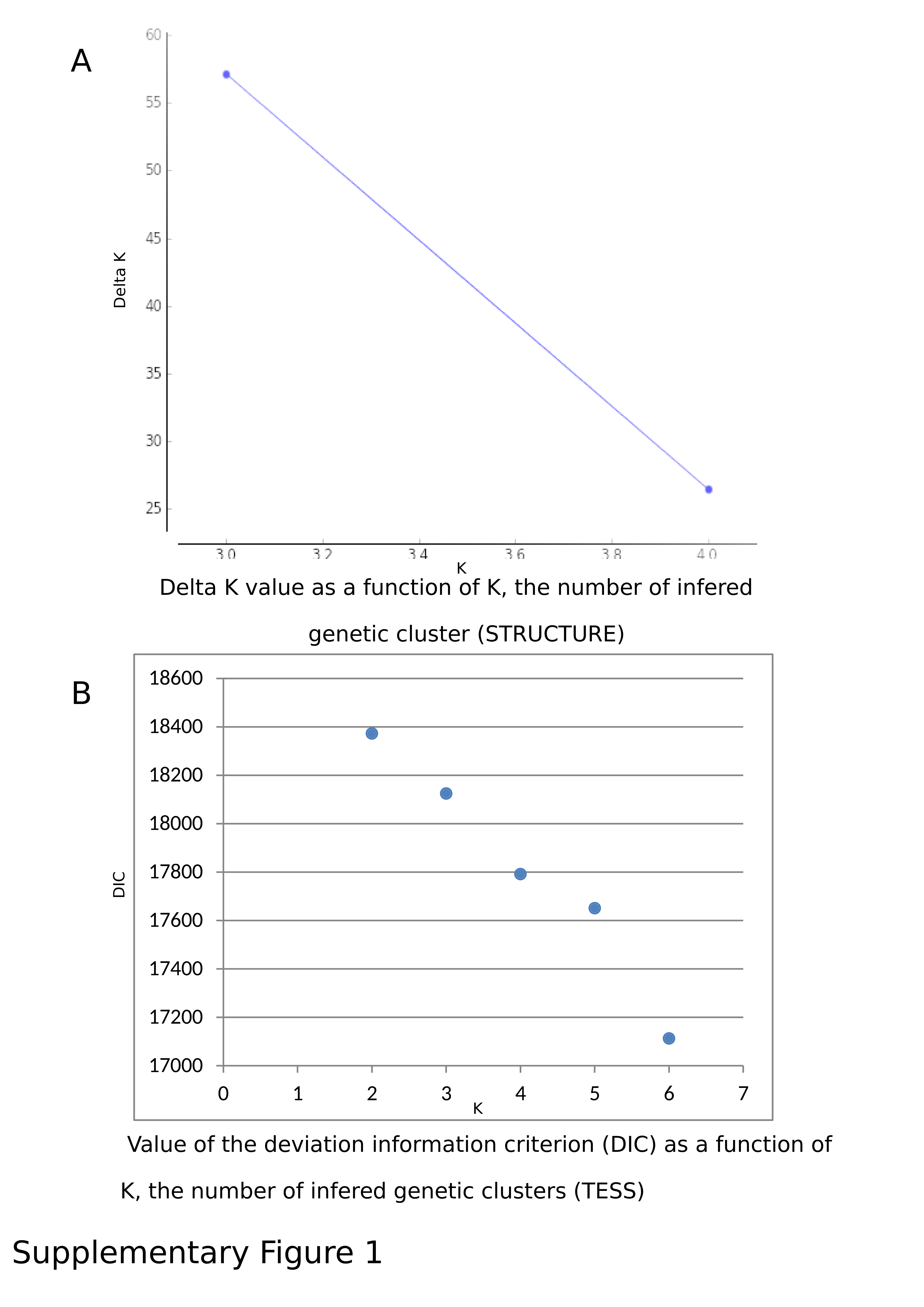

Supplement: S1 Fig — B- Value of the deviation information criterion as a function of K (TESS). K is the number of inferred genetic clusters. (TIFF) [file pone.0230434.s001.tiff]

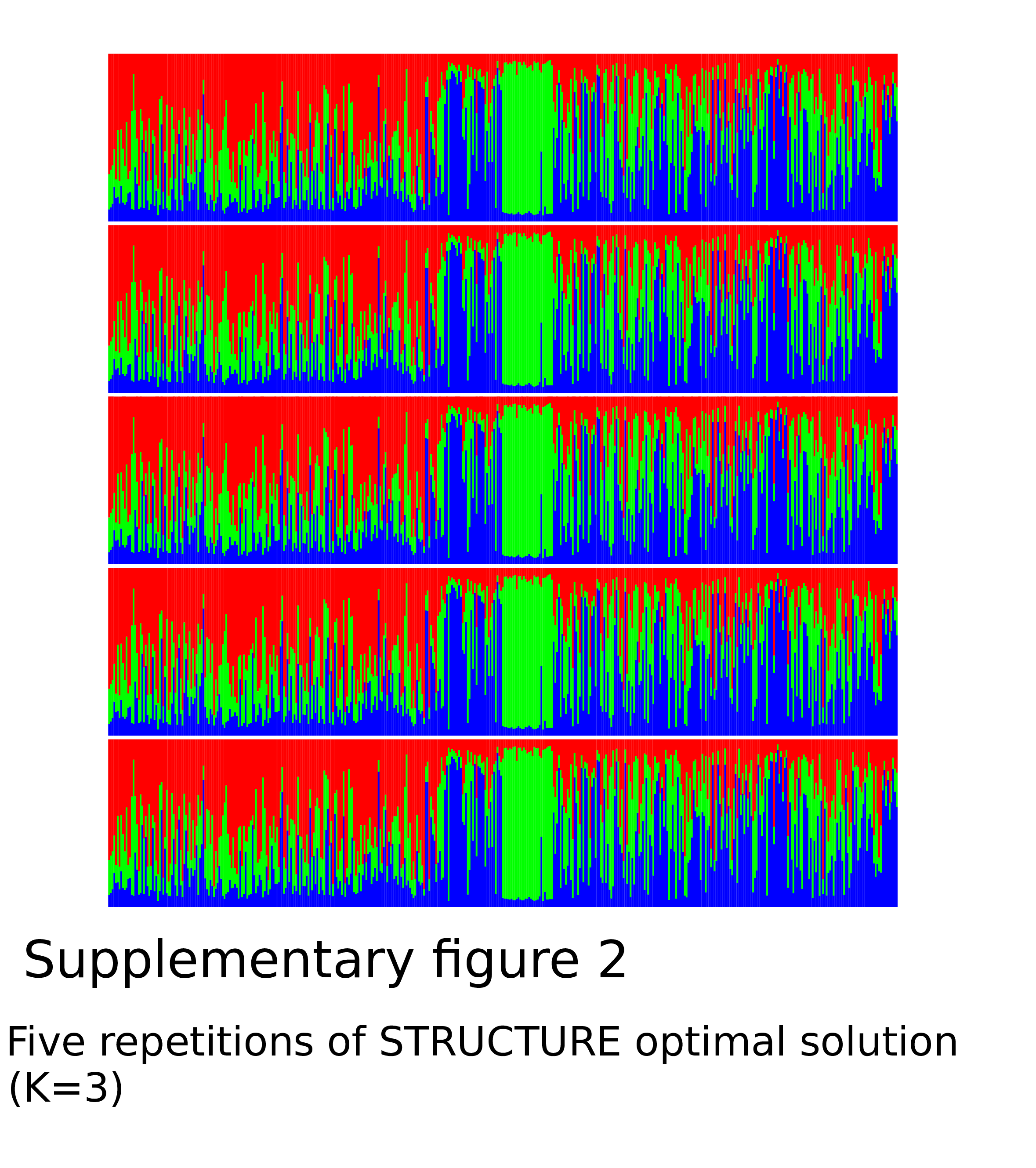

Supplement: S2 Fig — (TIFF) [file pone.0230434.s002.tiff]

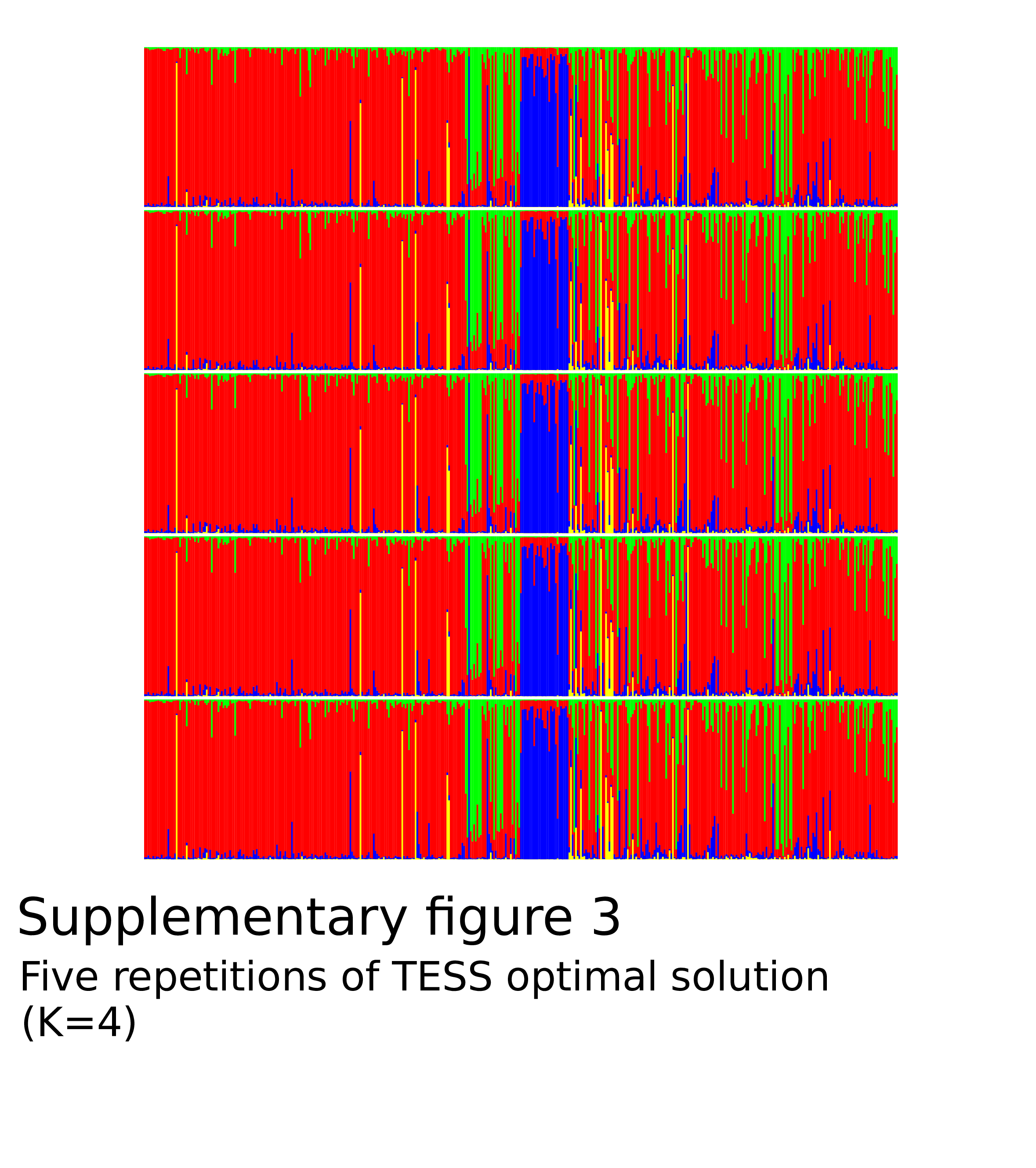

Supplement: S3 Fig — (TIFF) [file pone.0230434.s003.tiff]

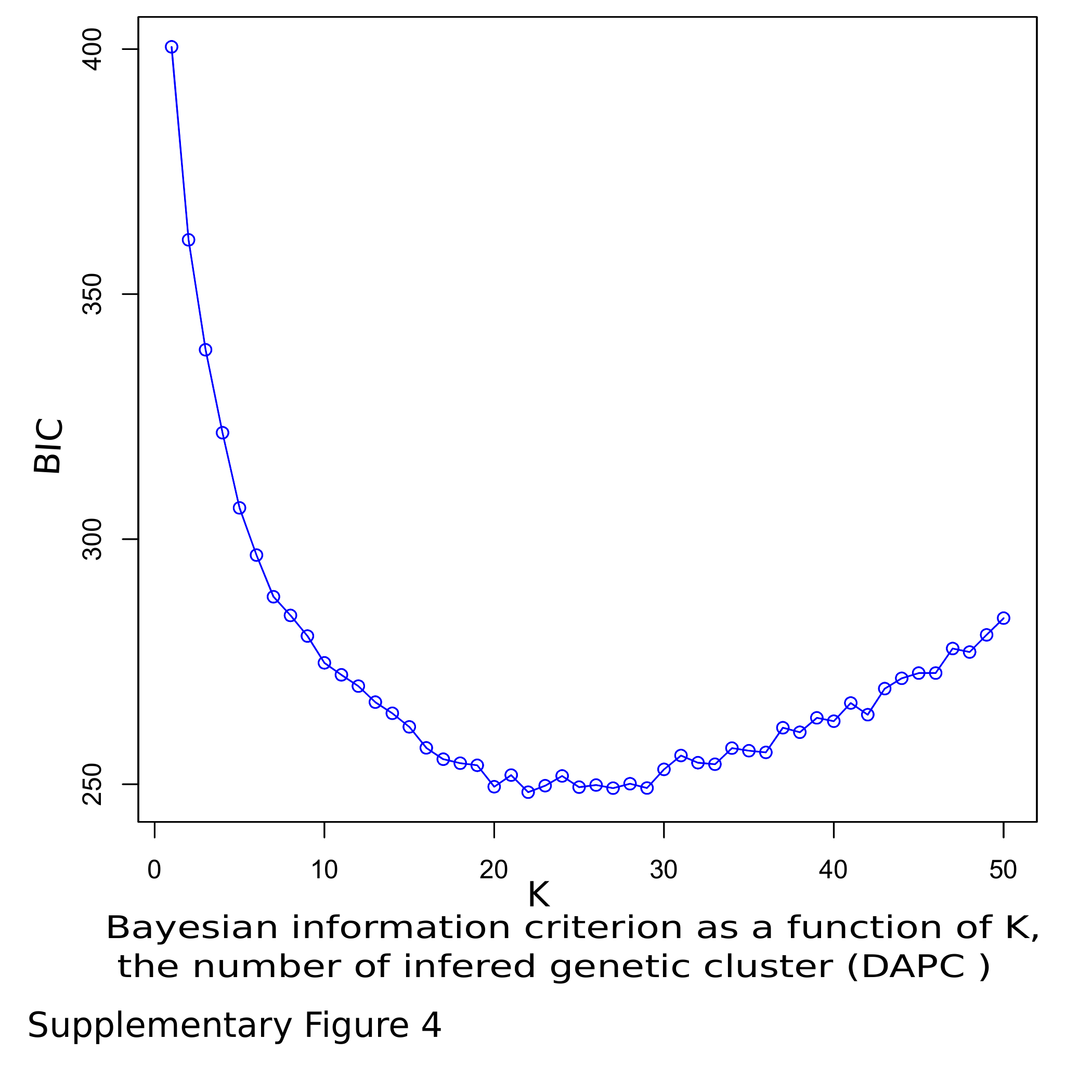

Supplement: S4 Fig — (TIF) [file pone.0230434.s004.tif]

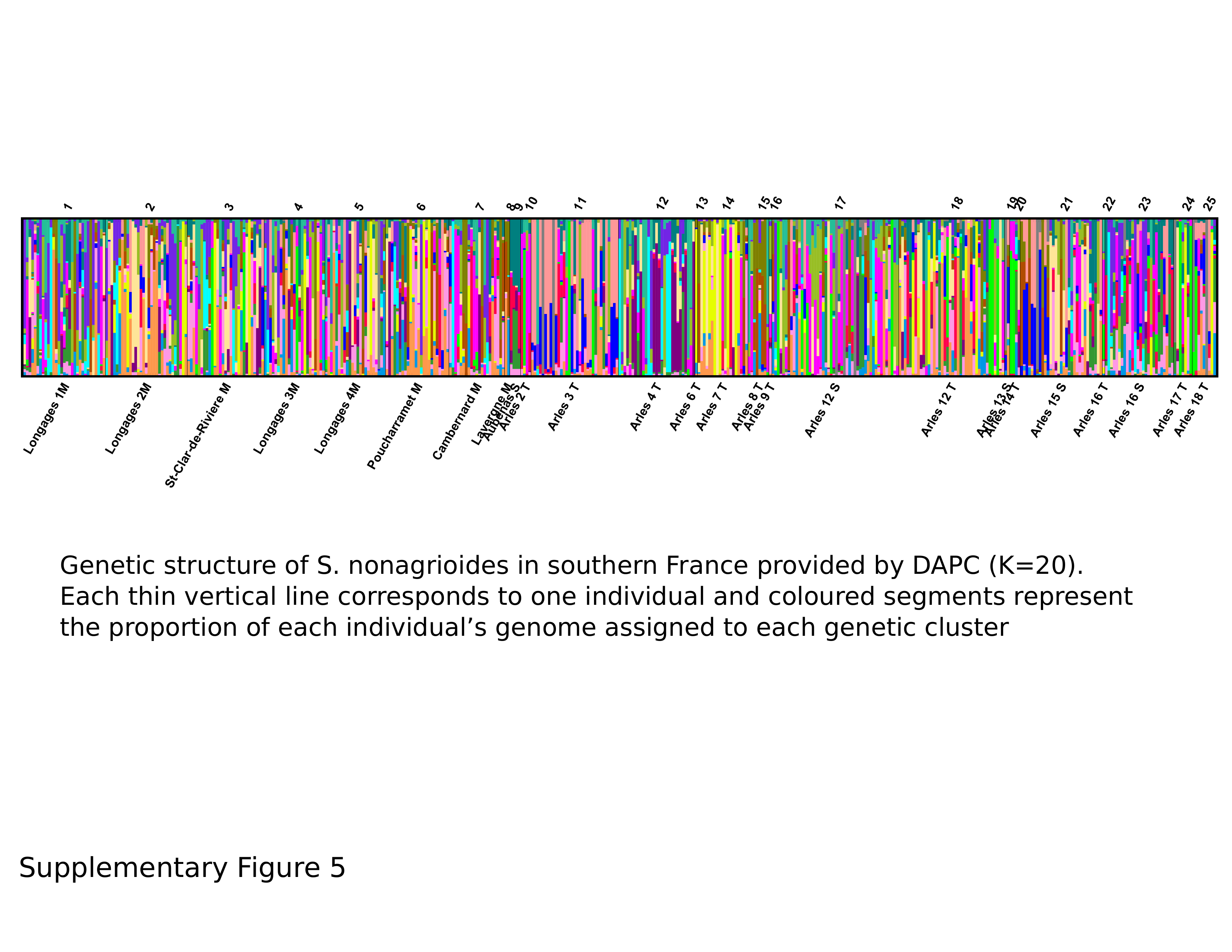

Supplement: S5 Fig — Each thin vertical line corresponds to one individual. Coloured segments represent the proportion of each individual’s genome assigned to each genetic cluster. (TIF) [file pone.0230434.s005.tif]
