# Supplementary material for: Population genetics of the Mediterranean corn borer (Sesamia nonagrioides) differs between wild and cultivated plants
Source: PLoS One. 2020 Mar 19;15(3):e0230434. doi: 10.1371/journal.pone.0230434 (PMC7081988; doi:10.1371/journal.pone.0230434)
Supplement: S1 Table — Ind: individual ID, Pop: Putative population of individuals, from Column 4, Microsatellite markers used. (DOCX) [file pone.0230434.s007.docx]

| Ind | pop | Latitude | Longitude | SN01 | SN01 | SN20 | SN20 | SN44 | SN44 | SN15 | SN15 | SN22 | SN22 | SN37 | SN37 | SN18 | SN18 | SN34 | SN34 | SN42 | SN42 | SN21 | SN21 | SN45 | SN45 |
| --- | --- | --- | --- | --- | --- | --- | --- | --- | --- | --- | --- | --- | --- | --- | --- | --- | --- | --- | --- | --- | --- | --- | --- | --- | --- |
| Long_1_5837 | 1 | 43.3695 | 1.1902 | 236 | 236 | 304 | 304 | 120 | 120 | 234 | 234 | 189 | 189 | 92 | 92 | 107 | 108 | 141 | 143 | 99 | 99 | 246 | 269 | 199 | 199 |
| Long_1_5838 | 1 | 43.3695 | 1.1902 | 236 | 236 | 304 | 304 | 120 | 120 | 234 | 234 | 189 | 191 | 90 | 96 | 107 | 107 | 141 | 141 | 99 | 99 | 247 | 269 | 199 | 199 |
| Long_1_5839 | 1 | 43.3695 | 1.1902 | 236 | 236 | 291 | 304 | 114 | 120 | 234 | 240 | 189 | 191 | 92 | 92 | 107 | 107 | 143 | 143 | 99 | 99 | 247 | 269 | 199 | 199 |
| Long_1_5840 | 1 | 43.3695 | 1.1902 | 236 | 236 | 291 | 306 | 120 | 120 | 234 | 234 | 189 | 191 | 92 | 92 | 107 | 107 | 141 | 143 | 99 | 108 | 246 | 247 | 199 | 199 |
| Long_1_5841 | 1 | 43.3695 | 1.1902 | 236 | 236 | 296 | 304 | 114 | 120 | 234 | 234 | 189 | 191 | 90 | 92 | 107 | 107 | 141 | 143 | 83 | 99 | 246 | 247 | 199 | 199 |
| Long_1_5842 | 1 | 43.3695 | 1.1902 | 236 | 236 | 291 | 304 | 120 | 120 | 223 | 234 | 188 | 189 | 90 | 90 | 107 | 108 | 141 | 143 | 99 | 99 | 247 | 247 | 199 | 199 |
| Long_1_5843 | 1 | 43.3695 | 1.1902 | 236 | 236 | 304 | 306 | 120 | 120 | 234 | 238 | 189 | 191 | 92 | 92 | 107 | 108 | 141 | 143 | 99 | 108 | 246 | 247 | 199 | 199 |
| Long_1_5844 | 1 | 43.3695 | 1.1902 | 236 | 236 | 304 | 306 | 114 | 114 | 234 | 234 | 189 | 189 | 90 | 92 | 107 | 111 | 139 | 141 | 99 | 99 | 246 | 247 | 199 | 199 |
| Long_1_5845 | 1 | 43.3695 | 1.1902 | 236 | 239 | 296 | 306 | 120 | 120 | 234 | 238 | 189 | 191 | 90 | 92 | 107 | 108 | 141 | 141 | 99 | 99 | 247 | 265 | 199 | 199 |
| Long_1_5846 | 1 | 43.3695 | 1.1902 | 236 | 236 | 304 | 306 | 120 | 123 | 234 | 238 | 189 | 189 | 92 | 92 | 107 | 114 | 141 | 141 | 99 | 99 | 246 | 247 | 199 | 199 |
| Long_1_5847 | 1 | 43.3695 | 1.1902 | 236 | 236 | 296 | 296 | 120 | 120 | 238 | 238 | 188 | 189 | 92 | 92 | 107 | 108 | 141 | 176 | 99 | 99 | 246 | 269 | 199 | 199 |
| Long_1_5848 | 1 | 43.3695 | 1.1902 | 236 | 236 | 304 | 304 | 120 | 120 | 223 | 234 | 189 | 189 | 90 | 92 | 107 | 108 | 143 | 176 | 83 | 83 | 246 | 265 | 199 | 199 |
| Long_1_5849 | 1 | 43.3695 | 1.1902 | 236 | 236 | 291 | 304 | 120 | 120 | 223 | 234 | 189 | 191 | 92 | 92 | 107 | 107 | 143 | 143 | 83 | 99 | 246 | 269 | 199 | 199 |
| Long_1_5850 | 1 | 43.3695 | 1.1902 | 236 | 236 | 306 | 306 | 120 | 123 | 234 | 234 | 189 | 189 | 92 | 92 | 107 | 108 | 141 | 141 | 99 | 99 | 247 | 247 | 199 | 199 |
| Long_1_5851 | 1 | 43.3695 | 1.1902 | 236 | 236 | 306 | 306 | 114 | 120 | 234 | 238 | 189 | 189 | 90 | 92 | 107 | 108 | 141 | 143 | 99 | 99 | 246 | 269 | 199 | 199 |
| Long_1_5852 | 1 | 43.3695 | 1.1902 | 236 | 236 | 296 | 306 | 120 | 120 | 234 | 240 | 189 | 191 | 92 | 96 | 107 | 108 | 141 | 143 | 99 | 99 | 246 | 265 | 199 | 199 |
| Long_1_5853 | 1 | 43.3695 | 1.1902 | 236 | 236 | 296 | 306 | 120 | 120 | 234 | 238 | 189 | 191 | 90 | 92 | 107 | 108 | 141 | 143 | 99 | 99 | 246 | 246 | 199 | 199 |
| Long_1_5854 | 1 | 43.3695 | 1.1902 | 236 | 236 | 304 | 304 | 114 | 120 | 223 | 238 | 189 | 191 | 90 | 92 | 107 | 108 | 143 | 143 | 99 | 99 | 246 | 247 | 199 | 199 |
| Long_1_5855 | 1 | 43.3695 | 1.1902 | 236 | 236 | 304 | 306 | 114 | 120 | 223 | 234 | 189 | 189 | 90 | 92 | 107 | 107 | 141 | 141 | 99 | 99 | 246 | 265 | 199 | 199 |
| Long_1_5856 | 1 | 43.3695 | 1.1902 | 239 | 239 | 300 | 300 | 120 | 120 | 234 | 234 | 189 | 189 | 92 | 92 | 107 | 114 | 141 | 141 | 83 | 99 | 269 | 269 | 199 | 199 |
| Long_1_5857 | 1 | 43.3695 | 1.1902 | 236 | 236 | 291 | 306 | 120 | 120 | 234 | 234 | 189 | 189 | 92 | 92 | 107 | 114 | 141 | 141 | 99 | 99 | 246 | 247 | 199 | 199 |
| Long_1_5858 | 1 | 43.3695 | 1.1902 | 236 | 236 | 296 | 304 | 114 | 120 | 223 | 234 | 188 | 189 | 90 | 92 | 107 | 114 | 141 | 143 | 83 | 99 | 246 | 246 | 199 | 199 |
| Long_1_5859 | 1 | 43.3695 | 1.1902 | 236 | 236 | 304 | 304 | 120 | 123 | 223 | 238 | 189 | 191 | 90 | 92 | 107 | 108 | 141 | 143 | 99 | 99 | 246 | 247 | 199 | 199 |
| Long_1_5860 | 1 | 43.3695 | 1.1902 | 236 | 236 | 291 | 306 | 120 | 120 | 234 | 234 | 189 | 189 | 92 | 92 | 107 | 108 | 141 | 141 | 99 | 99 | 246 | 247 | 199 | 199 |
| Long_1_5861 | 1 | 43.3695 | 1.1902 | 236 | 236 | 306 | 306 | 120 | 120 | 234 | 234 | 189 | 191 | 92 | 92 | 108 | 114 | 141 | 143 | 99 | 108 | 247 | 247 | 199 | 199 |
| Long_1_5862 | 1 | 43.3695 | 1.1902 | 236 | 236 | 298 | 310 | 120 | 120 | 223 | 234 | 189 | 189 | 92 | 92 | 107 | 108 | 139 | 143 | 83 | 83 | 246 | 269 | 199 | 199 |
| Long_1_5863 | 1 | 43.3695 | 1.1902 | 236 | 236 | 306 | 306 | 120 | 120 | 234 | 234 | 189 | 189 | 92 | 92 | 107 | 108 | 141 | 143 | 99 | 108 | 247 | 247 | 199 | 199 |
| Long_1_5864 | 1 | 43.3695 | 1.1902 | 236 | 236 | 304 | 304 | 120 | 120 | 223 | 234 | 189 | 191 | 90 | 92 | 107 | 108 | 143 | 143 | 83 | 99 | 246 | 265 | 199 | 199 |
| Long_1_5865 | 1 | 43.3695 | 1.1902 | 236 | 236 | 304 | 304 | 120 | 120 | 234 | 238 | 189 | 189 | 92 | 92 | 107 | 108 | 141 | 141 | 99 | 99 | 247 | 247 | 199 | 199 |
| Long_1_5866 | 1 | 43.3695 | 1.1902 | 236 | 236 | 306 | 306 | 120 | 120 | 234 | 238 | 189 | 189 | 90 | 90 | 107 | 108 | 141 | 176 | 99 | 99 | 246 | 269 | 199 | 199 |
| Long_1_5867 | 1 | 43.3695 | 1.1902 | 236 | 236 | 296 | 296 | 120 | 120 | 234 | 234 | 189 | 189 | 96 | 96 | 107 | 111 | 143 | 143 | 83 | 99 | 246 | 247 | 199 | 199 |
| Long_2_5869 | 2 | 43.3746 | 1.2007 | 236 | 236 | 304 | 306 | 120 | 123 | 223 | 234 | 189 | 189 | 92 | 94 | 114 | 114 | 141 | 141 | 99 | 99 | 247 | 247 | 199 | 199 |
| Long_2_5870 | 2 | 43.3746 | 1.2007 | 236 | 236 | 296 | 310 | 120 | 120 | 234 | 234 | 189 | 189 | 92 | 94 | 107 | 114 | 141 | 143 | 83 | 99 | 246 | 246 | 199 | 199 |
| Long_2_5872 | 2 | 43.3746 | 1.2007 | 236 | 236 | 296 | 304 | 120 | 120 | 234 | 238 | 189 | 189 | 92 | 96 | 107 | 107 | 143 | 143 | 99 | 99 | 269 | 269 | 199 | 199 |
| Long_2_5873 | 2 | 43.3746 | 1.2007 | 236 | 236 | 291 | 304 | 120 | 120 | 223 | 223 | 189 | 191 | 92 | 92 | 107 | 108 | 141 | 141 | 83 | 99 | 246 | 247 | 199 | 199 |
| Long_2_5874 | 2 | 43.3746 | 1.2007 | 236 | 239 | 291 | 304 | 114 | 120 | 223 | 234 | 189 | 191 | 92 | 92 | 107 | 114 | 141 | 141 | 99 | 99 | 269 | 269 | 199 | 199 |
| Long_2_5875 | 2 | 43.3746 | 1.2007 | 236 | 236 | 304 | 306 | 120 | 120 | 234 | 234 | 188 | 189 | 92 | 96 | 107 | 107 | 141 | 141 | 83 | 99 | 269 | 269 | 199 | 199 |
| Long_2_5876 | 2 | 43.3746 | 1.2007 | 234 | 236 | 304 | 304 | 114 | 120 | 234 | 234 | 189 | 189 | 90 | 96 | 107 | 107 | 141 | 143 | 83 | 83 | 246 | 269 | 199 | 199 |
| Long_2_5877 | 2 | 43.3746 | 1.2007 | 236 | 236 | 296 | 306 | 120 | 120 | 234 | 238 | 189 | 189 | 90 | 92 | 107 | 114 | 141 | 143 | 83 | 83 | 247 | 269 | 199 | 199 |
| Long_2_5878 | 2 | 43.3746 | 1.2007 | 236 | 236 | 291 | 304 | 114 | 120 | 234 | 234 | 189 | 189 | 90 | 92 | 108 | 108 | 141 | 176 | 83 | 99 | 265 | 269 | 199 | 199 |
| Long_2_5879 | 2 | 43.3746 | 1.2007 | 236 | 236 | 296 | 306 | 120 | 120 | 223 | 234 | 189 | 191 | 90 | 90 | 107 | 114 | 141 | 143 | 83 | 83 | 246 | 265 | 199 | 199 |
| Long_2_5880 | 2 | 43.3746 | 1.2007 | 236 | 236 | 296 | 306 | 120 | 120 | 223 | 238 | 189 | 189 | 90 | 92 | 107 | 107 | 143 | 143 | 83 | 99 | 247 | 247 | 199 | 199 |
| Long_2_5881 | 2 | 43.3746 | 1.2007 | 236 | 236 | 304 | 304 | 120 | 120 | 234 | 234 | 189 | 189 | 92 | 94 | 107 | 114 | 143 | 143 | 99 | 99 | 247 | 269 | 199 | 199 |
| Long_2_5882 | 2 | 43.3746 | 1.2007 | 236 | 236 | 291 | 306 | 114 | 120 | 234 | 240 | 189 | 189 | 92 | 96 | 107 | 111 | 141 | 143 | 83 | 99 | 247 | 265 | 199 | 199 |
| Long_2_5883 | 2 | 43.3746 | 1.2007 | 236 | 236 | 291 | 310 | 120 | 120 | 210 | 234 | 189 | 191 | 90 | 90 | 107 | 108 | 143 | 176 | 83 | 99 | 246 | 247 | 199 | 199 |
| Long_2_5884 | 2 | 43.3746 | 1.2007 | 236 | 236 | 304 | 306 | 114 | 120 | 234 | 234 | 189 | 191 | 90 | 94 | 107 | 107 | 141 | 143 | 83 | 83 | 246 | 269 | 199 | 199 |
| Long_2_5885 | 2 | 43.3746 | 1.2007 | 236 | 236 | 296 | 304 | 120 | 120 | 234 | 234 | 189 | 189 | 90 | 92 | 107 | 107 | 143 | 143 | 99 | 99 | 265 | 269 | 199 | 199 |
| Long_2_5886 | 2 | 43.3746 | 1.2007 | 236 | 236 | 296 | 306 | 120 | 120 | 223 | 234 | 191 | 191 | 92 | 94 | 107 | 107 | 141 | 143 | 83 | 83 | 246 | 247 | 199 | 199 |
| Long_2_5887 | 2 | 43.3746 | 1.2007 | 236 | 236 | 291 | 296 | 120 | 120 | 238 | 238 | 189 | 189 | 90 | 90 | 107 | 107 | 143 | 176 | 83 | 108 | 269 | 269 | 199 | 199 |
| Long_2_5888 | 2 | 43.3746 | 1.2007 | 236 | 236 | 304 | 304 | 120 | 120 | 223 | 223 | 189 | 189 | 90 | 92 | 107 | 107 | 143 | 143 | 83 | 83 | 246 | 269 | 199 | 199 |
| Long_2_5889 | 2 | 43.3746 | 1.2007 | 236 | 236 | 304 | 304 | 114 | 120 | 223 | 223 | 189 | 189 | 90 | 96 | 107 | 111 | 143 | 143 | 83 | 83 | 246 | 269 | 199 | 199 |
| Long_2_5890 | 2 | 43.3746 | 1.2007 | 236 | 236 | 291 | 306 | 114 | 120 | 223 | 223 | 189 | 189 | 90 | 92 | 108 | 108 | 141 | 143 | 99 | 99 | 246 | 247 | 199 | 199 |
| Long_2_5891 | 2 | 43.3746 | 1.2007 | 236 | 236 | 304 | 306 | 120 | 120 | 234 | 234 | 189 | 191 | 92 | 96 | 107 | 107 | 141 | 143 | 99 | 108 | 246 | 269 | 199 | 199 |
| Long_2_5892 | 2 | 43.3746 | 1.2007 | 236 | 236 | 291 | 310 | 120 | 120 | 234 | 238 | 189 | 189 | 90 | 92 | 107 | 111 | 141 | 143 | 99 | 99 | 246 | 246 | 199 | 199 |
| Long_2_5893 | 2 | 43.3746 | 1.2007 | 236 | 236 | 291 | 296 | 120 | 120 | 234 | 234 | 189 | 191 | 90 | 92 | 107 | 107 | 139 | 139 | 99 | 108 | 246 | 246 | 199 | 199 |
| Long_2_5894 | 2 | 43.3746 | 1.2007 | 236 | 236 | 296 | 306 | 120 | 120 | 234 | 238 | 189 | 191 | 92 | 92 | 107 | 107 | 141 | 143 | 99 | 108 | 246 | 247 | 199 | 199 |
| Long_2_5895 | 2 | 43.3746 | 1.2007 | 236 | 236 | 306 | 306 | 114 | 120 | 234 | 238 | 189 | 191 | 92 | 92 | 107 | 111 | 141 | 143 | 99 | 99 | 247 | 265 | 199 | 199 |
| Long_2_5896 | 2 | 43.3746 | 1.2007 | 236 | 236 | 296 | 306 | 120 | 120 | 234 | 238 | 189 | 189 | 90 | 92 | 107 | 114 | 141 | 141 | 99 | 99 | 269 | 269 | 199 | 199 |
| Long_2_5897 | 2 | 43.3746 | 1.2007 | 236 | 239 | 306 | 306 | 120 | 120 | 223 | 238 | 191 | 191 | 90 | 92 | 108 | 108 | 143 | 143 | 99 | 99 | 247 | 269 | 199 | 199 |
| Long_2_5898 | 2 | 43.3746 | 1.2007 | 236 | 236 | 291 | 304 | 114 | 120 | 223 | 234 | 189 | 189 | 90 | 90 | 107 | 108 | 141 | 141 | 99 | 108 | 246 | 269 | 199 | 199 |
| Long_2_5899 | 2 | 43.3746 | 1.2007 | 236 | 236 | 304 | 304 | 120 | 120 | 234 | 234 | 189 | 191 | 90 | 92 | 114 | 114 | 141 | 141 | 83 | 99 | 247 | 265 | 199 | 199 |
| Long_2_5900 | 2 | 43.3746 | 1.2007 | 236 | 236 | 291 | 304 | 114 | 120 | 223 | 234 | 189 | 189 | 90 | 92 | 108 | 114 | 141 | 143 | 83 | 99 | 246 | 265 | 199 | 199 |
| St_5902 | 3 | 43.448 | 1.1937 | 236 | 236 | 304 | 304 | 120 | 120 | 234 | 238 | 189 | 189 | 90 | 94 | 107 | 114 | 143 | 143 | 99 | 108 | 246 | 269 | 199 | 199 |
| St_5903 | 3 | 43.448 | 1.1937 | 236 | 236 | 296 | 306 | 120 | 120 | 234 | 238 | 191 | 191 | 90 | 92 | 107 | 107 | 143 | 143 | 83 | 99 | 269 | 269 | 199 | 199 |
| St_5904 | 3 | 43.448 | 1.1937 | 236 | 236 | 296 | 304 | 120 | 120 | 234 | 234 | 189 | 189 | 90 | 96 | 107 | 107 | 141 | 143 | 83 | 99 | 247 | 269 | 199 | 199 |
| St_5905 | 3 | 43.448 | 1.1937 | 236 | 236 | 291 | 291 | 120 | 120 | 234 | 234 | 189 | 189 | 90 | 90 | 108 | 114 | 141 | 141 | 99 | 99 | 247 | 269 | 199 | 199 |
| St_5906 | 3 | 43.448 | 1.1937 | 236 | 236 | 304 | 306 | 120 | 120 | 238 | 238 | 189 | 189 | 90 | 92 | 107 | 107 | 141 | 141 | 99 | 99 | 247 | 265 | 199 | 199 |
| St_5907 | 3 | 43.448 | 1.1937 | 236 | 236 | 304 | 306 | 120 | 120 | 234 | 234 | 189 | 189 | 90 | 92 | 107 | 107 | 141 | 143 | 99 | 99 | 265 | 269 | 199 | 199 |
| St_5908 | 3 | 43.448 | 1.1937 | 236 | 236 | 296 | 296 | 120 | 120 | 234 | 238 | 189 | 191 | 96 | 96 | 108 | 114 | 141 | 141 | 99 | 99 | 247 | 269 | 199 | 199 |
| St_5909 | 3 | 43.448 | 1.1937 | 236 | 236 | 304 | 306 | 120 | 120 | 234 | 238 | 189 | 191 | 92 | 92 | 107 | 108 | 139 | 143 | 99 | 99 | 246 | 247 | 199 | 199 |
| St_5910 | 3 | 43.448 | 1.1937 | 236 | 236 | 296 | 304 | 120 | 123 | 234 | 238 | 189 | 191 | 92 | 92 | 107 | 108 | 141 | 143 | 83 | 99 | 247 | 269 | 199 | 199 |
| St_5912 | 3 | 43.448 | 1.1937 | 236 | 236 | 306 | 306 | 114 | 114 | 234 | 238 | 191 | 191 | 90 | 90 | 107 | 114 | 141 | 141 | 99 | 99 | 246 | 247 | 199 | 199 |
| St_5913 | 3 | 43.448 | 1.1937 | 236 | 236 | 306 | 306 | 120 | 120 | 234 | 238 | 189 | 191 | 92 | 94 | 107 | 107 | 141 | 141 | 83 | 83 | 246 | 247 | 199 | 199 |
| St_5914 | 3 | 43.448 | 1.1937 | 236 | 236 | 296 | 304 | 120 | 120 | 223 | 234 | 189 | 191 | 92 | 94 | 107 | 107 | 143 | 143 | 83 | 99 | 247 | 265 | 199 | 199 |
| St_5915 | 3 | 43.448 | 1.1937 | 236 | 236 | 304 | 306 | 114 | 120 | 223 | 234 | 189 | 189 | 90 | 90 | 108 | 108 | 141 | 141 | 99 | 99 | 247 | 247 | -9 | -9 |
| St_5916 | 3 | 43.448 | 1.1937 | 236 | 239 | -9 | -9 | 120 | 120 | 223 | 234 | 189 | 189 | 90 | 92 | 107 | 114 | 141 | 143 | 99 | 108 | 246 | 269 | 199 | 199 |
| St_5917 | 3 | 43.448 | 1.1937 | 236 | 236 | 304 | 304 | 120 | 120 | 238 | 238 | 189 | 189 | 92 | 92 | 107 | 108 | 143 | 143 | 99 | 108 | 247 | 269 | 199 | 199 |
| St_5918 | 3 | 43.448 | 1.1937 | 236 | 236 | 296 | 304 | 120 | 120 | 234 | 238 | 189 | 189 | 90 | 92 | 107 | 108 | 143 | 143 | 99 | 99 | 247 | 265 | 199 | 199 |
| St_5919 | 3 | 43.448 | 1.1937 | 236 | 236 | 304 | 310 | 120 | 120 | 223 | 223 | 189 | 191 | 90 | 90 | 107 | 107 | 141 | 143 | 83 | 99 | 246 | 247 | 199 | 199 |
| St_5920 | 3 | 43.448 | 1.1937 | 236 | 236 | 291 | 304 | 114 | 120 | 223 | 234 | -9 | -9 | 92 | 92 | 107 | 114 | 141 | 141 | 99 | 99 | 269 | 269 | 199 | 199 |
| St_5921 | 3 | 43.448 | 1.1937 | 236 | 236 | 296 | 304 | 114 | 120 | 234 | 234 | 189 | 189 | 92 | 92 | 107 | 107 | 141 | 141 | 99 | 99 | 246 | 269 | 199 | 199 |
| St_5922 | 3 | 43.448 | 1.1937 | 236 | 236 | 304 | 306 | 114 | 120 | 223 | 234 | 189 | 189 | 90 | 92 | 107 | 108 | 141 | 141 | 83 | 99 | 265 | 269 | 199 | 199 |
| St_5923 | 3 | 43.448 | 1.1937 | 236 | 236 | 296 | 306 | 120 | 120 | 234 | 234 | 189 | 191 | 92 | 94 | 111 | 111 | 141 | 143 | 108 | 108 | 246 | 247 | 199 | 199 |
| St_5925 | 3 | 43.448 | 1.1937 | 236 | 236 | 306 | 306 | 120 | 120 | 223 | 238 | 191 | 191 | 90 | 96 | 107 | 114 | 141 | 141 | 99 | 99 | 246 | 269 | 199 | 199 |
| St_5926 | 3 | 43.448 | 1.1937 | 236 | 236 | 296 | 296 | 114 | 114 | 223 | 234 | 189 | 189 | 92 | 92 | 107 | 107 | 141 | 143 | 99 | 99 | 246 | 269 | 199 | 199 |
| St_5927 | 3 | 43.448 | 1.1937 | 236 | 236 | 304 | 306 | 120 | 120 | 223 | 234 | 189 | 191 | 92 | 92 | 107 | 107 | 141 | 141 | 83 | 83 | 265 | 265 | 199 | 199 |
| St_5928 | 3 | 43.448 | 1.1937 | 236 | 236 | 296 | 304 | 120 | 120 | 223 | 234 | 189 | 191 | 90 | 92 | 107 | 107 | 141 | 143 | 83 | 99 | 246 | 269 | 199 | 199 |
| St_5929 | 3 | 43.448 | 1.1937 | 236 | 236 | 296 | 304 | 120 | 120 | 234 | 234 | 189 | 191 | 90 | 92 | 107 | 107 | 141 | 143 | 99 | 99 | 246 | 269 | 199 | 199 |
| St_5930 | 3 | 43.448 | 1.1937 | 236 | 236 | 296 | 306 | 114 | 120 | 223 | 223 | 189 | 189 | 92 | 92 | 107 | 107 | 141 | 143 | 99 | 99 | 269 | 269 | 199 | 199 |
| St_5931 | 3 | 43.448 | 1.1937 | 236 | 236 | 296 | 306 | 120 | 120 | 223 | 238 | 189 | 189 | 90 | 96 | 107 | 114 | 141 | 143 | 99 | 99 | 247 | 269 | 199 | 199 |
| St_5932 | 3 | 43.448 | 1.1937 | 236 | 236 | 304 | 304 | 120 | 120 | 223 | 223 | 191 | 191 | 90 | 90 | 107 | 107 | 141 | 143 | 83 | 99 | 246 | 265 | 199 | 199 |
| Long_3_8085 | 4 | 43.3680 | 1.1926 | 236 | 236 | 296 | 304 | 120 | 120 | 234 | 238 | 191 | 191 | 92 | 94 | 107 | 107 | 141 | 141 | 99 | 108 | 269 | 269 | 199 | 199 |
| Long_3_8086 | 4 | 43.3680 | 1.1926 | 236 | 236 | 304 | 306 | 120 | 120 | 223 | 234 | 189 | 189 | 92 | 92 | 107 | 108 | 139 | 141 | 99 | 99 | 269 | 269 | 199 | 199 |
| Long_3_8087 | 4 | 43.3680 | 1.1926 | 236 | 236 | 298 | 304 | 114 | 120 | 223 | 223 | 191 | 191 | 90 | 92 | 107 | 114 | 143 | 143 | 83 | 83 | 246 | 246 | 199 | 199 |
| Long_3_8088 | 4 | 43.3680 | 1.1926 | 234 | 236 | 296 | 304 | 114 | 120 | 234 | 234 | 189 | 189 | 90 | 92 | 107 | 108 | 139 | 141 | 99 | 99 | 247 | 265 | 199 | 199 |
| Long_3_8089 | 4 | 43.3680 | 1.1926 | 236 | 236 | 306 | 306 | 114 | 114 | 223 | 223 | 189 | 191 | 92 | 94 | 107 | 107 | 141 | 143 | 99 | 108 | 246 | 247 | 199 | 199 |
| Long_3_8090 | 4 | 43.3680 | 1.1926 | 236 | 236 | 306 | 310 | 120 | 120 | 234 | 238 | 189 | 189 | 92 | 92 | 107 | 107 | 141 | 141 | 99 | 99 | 246 | 247 | 199 | 199 |
| Long_3_8091 | 4 | 43.3680 | 1.1926 | 236 | 236 | 304 | 304 | 120 | 120 | 223 | 238 | 189 | 191 | 90 | 90 | 107 | 107 | 141 | 143 | 83 | 99 | 246 | 269 | 199 | 199 |
| Long_3_8092 | 4 | 43.3680 | 1.1926 | 236 | 236 | 304 | 306 | 120 | 120 | 234 | 234 | 188 | 188 | 90 | 92 | 107 | 107 | 143 | 143 | 83 | 99 | 247 | 247 | 199 | 199 |
| Long_3_8093 | 4 | 43.3680 | 1.1926 | 236 | 236 | 296 | 306 | 120 | 120 | 223 | 234 | 189 | 189 | 90 | 90 | 107 | 108 | 139 | 139 | 83 | 108 | 265 | 269 | 199 | 199 |
| Long_3_8094 | 4 | 43.3680 | 1.1926 | 236 | 236 | 304 | 304 | 114 | 120 | 223 | 223 | 189 | 191 | 90 | 92 | 107 | 107 | 143 | 143 | 99 | 99 | 269 | 269 | 199 | 199 |
| Long_3_8095 | 4 | 43.3680 | 1.1926 | -9 | -9 | 304 | 306 | 120 | 120 | 223 | 234 | 189 | 191 | 92 | 92 | 107 | 107 | 141 | 141 | 83 | 99 | 246 | 269 | 199 | 199 |
| Long_3_8096 | 4 | 43.3680 | 1.1926 | -9 | -9 | 304 | 310 | 114 | 120 | 234 | 234 | 189 | 189 | 90 | 90 | 108 | 111 | 143 | 143 | 83 | 99 | 246 | 269 | 199 | 199 |
| Long_3_8133 | 4 | 43.3680 | 1.1926 | 236 | 236 | 291 | 291 | 120 | 120 | 234 | 234 | 191 | 191 | 90 | 92 | 107 | 107 | 141 | 141 | 99 | 99 | 246 | 269 | 199 | 199 |
| Long_3_8134 | 4 | 43.3680 | 1.1926 | 236 | 236 | 296 | 304 | 120 | 120 | 234 | 234 | 189 | 191 | 92 | 96 | 107 | 111 | 143 | 143 | 99 | 99 | 246 | 269 | 199 | 199 |
| Long_3_8135 | 4 | 43.3680 | 1.1926 | 236 | 236 | 296 | 304 | 114 | 120 | 234 | 234 | 189 | 189 | 90 | 92 | 107 | 108 | 141 | 141 | 99 | 99 | 246 | 269 | 199 | 199 |
| Long_3_8136 | 4 | 43.3680 | 1.1926 | 236 | 236 | 296 | 296 | 114 | 120 | 234 | 234 | 189 | 189 | 92 | 92 | 108 | 111 | 141 | 141 | 83 | 99 | 265 | 265 | 199 | 199 |
| Long_3_8138 | 4 | 43.3680 | 1.1926 | 236 | 236 | 304 | 306 | 120 | 120 | 234 | 238 | 189 | 191 | 92 | 94 | 107 | 111 | 143 | 143 | 99 | 99 | 247 | 247 | 199 | 199 |
| Long_3_8139 | 4 | 43.3680 | 1.1926 | 236 | 236 | 291 | 291 | 120 | 123 | 234 | 234 | 189 | 189 | 90 | 90 | 107 | 114 | 141 | 143 | 99 | 108 | 246 | 265 | 199 | 199 |
| Long_3_8140 | 4 | 43.3680 | 1.1926 | 236 | 236 | 304 | 304 | 120 | 120 | 223 | 234 | 189 | 189 | 90 | 92 | 107 | 107 | 141 | 143 | 99 | 99 | 265 | 269 | 199 | 199 |
| Long_3_8141 | 4 | 43.3680 | 1.1926 | 236 | 236 | 296 | 304 | 120 | 120 | 223 | 234 | 188 | 189 | 92 | 92 | 107 | 114 | 141 | 143 | 83 | 108 | 246 | 247 | 199 | 199 |
| Long_3_8142 | 4 | 43.3680 | 1.1926 | 236 | 236 | 291 | 306 | 114 | 120 | 223 | 234 | 189 | 189 | 90 | 90 | 107 | 114 | 141 | 141 | 83 | 83 | 247 | 269 | 199 | 199 |
| Long_3_8143 | 4 | 43.3680 | 1.1926 | 236 | 236 | 296 | 304 | 120 | 120 | 234 | 234 | 189 | 191 | 90 | 90 | 107 | 114 | 141 | 143 | 99 | 99 | 247 | 265 | 199 | 199 |
| Long_3_8144 | 4 | 43.3680 | 1.1926 | 236 | 236 | 304 | 306 | 120 | 120 | 234 | 238 | 188 | 189 | 90 | 92 | 107 | 107 | 141 | 143 | 83 | 99 | 247 | 247 | 199 | 199 |
| Long_4_8098 | 5 | 43.3699 | 1.199 | 236 | 236 | 304 | 306 | 114 | 120 | 234 | 238 | 189 | 191 | 90 | 94 | 107 | 108 | 141 | 141 | 99 | 108 | 247 | 269 | 199 | 199 |
| Long_4_8099 | 5 | 43.3699 | 1.199 | 236 | 236 | -9 | -9 | 114 | 120 | 234 | 234 | 189 | 189 | 90 | 94 | 107 | 111 | 139 | 143 | 83 | 99 | 246 | 246 | 199 | 199 |
| Long_4_8100 | 5 | 43.3699 | 1.199 | 236 | 236 | 296 | 304 | 120 | 120 | 223 | 238 | 189 | 191 | 90 | 92 | 107 | 107 | 141 | 141 | 99 | 99 | 246 | 265 | 199 | 199 |
| Long_4_8101 | 5 | 43.3699 | 1.199 | 236 | 236 | 304 | 304 | 120 | 120 | 223 | 234 | 189 | 189 | 90 | 90 | 107 | 107 | 143 | 143 | 83 | 99 | 246 | 265 | 199 | 199 |
| Long_4_8102 | 5 | 43.3699 | 1.199 | 236 | 236 | 296 | 296 | 114 | 120 | 238 | 238 | 188 | 189 | 90 | 92 | 107 | 108 | 141 | 143 | 83 | 99 | 247 | 269 | 199 | 199 |
| Long_4_8103 | 5 | 43.3699 | 1.199 | 236 | 236 | 291 | 296 | 114 | 120 | 234 | 238 | 189 | 191 | 90 | 96 | 107 | 107 | 141 | 143 | 83 | 99 | 246 | 246 | 199 | 199 |
| Long_4_8104 | 5 | 43.3699 | 1.199 | 236 | 236 | 304 | 304 | 114 | 120 | 234 | 234 | 189 | 189 | 92 | 96 | 107 | 107 | 141 | 141 | 99 | 99 | 246 | 265 | 199 | 199 |
| Long_4_8105 | 5 | 43.3699 | 1.199 | 236 | 236 | 304 | 304 | 114 | 120 | 234 | 234 | 191 | 191 | 90 | 90 | 107 | 107 | 143 | 143 | 83 | 99 | 269 | 269 | 199 | 199 |
| Long_4_8106 | 5 | 43.3699 | 1.199 | 236 | 236 | 304 | 306 | 120 | 120 | 223 | 240 | 189 | 189 | 92 | 92 | 108 | 108 | 141 | 143 | 83 | 99 | 269 | 269 | 199 | 199 |
| Long_4_8107 | 5 | 43.3699 | 1.199 | 236 | 236 | 291 | 306 | 114 | 120 | 223 | 238 | 189 | 191 | 92 | 96 | 107 | 108 | 143 | 143 | 99 | 99 | 269 | 269 | 199 | 199 |
| Long_4_8108 | 5 | 43.3699 | 1.199 | 236 | 236 | 296 | 306 | 120 | 120 | 223 | 240 | 189 | 189 | 90 | 96 | 107 | 107 | 143 | 143 | 83 | 99 | 265 | 269 | 199 | 199 |
| Long_4_8145 | 5 | 43.3699 | 1.199 | 236 | 236 | 291 | 296 | 114 | 120 | 234 | 234 | 188 | 189 | 90 | 94 | 107 | 107 | 143 | 143 | 83 | 99 | 246 | 246 | 199 | 199 |
| Long_4_8146 | 5 | 43.3699 | 1.199 | 236 | 236 | 296 | 306 | 120 | 120 | 238 | 238 | 189 | 191 | 90 | 92 | 107 | 107 | 141 | 143 | 99 | 99 | 247 | 247 | 199 | 199 |
| Long_4_8147 | 5 | 43.3699 | 1.199 | 236 | 236 | 304 | 306 | 120 | 120 | 223 | 238 | 189 | 191 | 90 | 92 | 107 | 107 | 143 | 143 | 99 | 108 | 246 | 246 | 199 | 199 |
| Long_4_8148 | 5 | 43.3699 | 1.199 | 236 | 236 | 304 | 304 | 120 | 120 | 234 | 234 | 189 | 189 | 92 | 92 | 108 | 114 | 141 | 143 | 83 | 83 | 269 | 269 | 199 | 199 |
| Long_4_8149 | 5 | 43.3699 | 1.199 | 239 | 239 | 300 | 308 | 114 | 114 | 234 | 234 | 191 | 191 | 90 | 92 | 107 | 107 | 141 | 143 | 83 | 99 | 247 | 269 | 199 | 199 |
| Long_4_8150 | 5 | 43.3699 | 1.199 | 236 | 236 | 306 | 306 | 114 | 120 | 234 | 234 | 189 | 189 | 92 | 96 | 107 | 107 | 141 | 143 | 83 | 99 | 246 | 247 | 199 | 199 |
| Long_4_8151 | 5 | 43.3699 | 1.199 | 236 | 236 | 291 | 304 | 120 | 120 | 234 | 238 | 189 | 189 | 90 | 92 | 107 | 107 | 141 | 141 | 83 | 83 | 246 | 247 | -9 | -9 |
| Long_4_8152 | 5 | 43.3699 | 1.199 | 236 | 236 | 296 | 304 | 120 | 120 | 223 | 223 | 189 | 189 | 90 | 90 | 107 | 107 | 141 | 143 | 99 | 108 | 247 | 269 | 199 | 199 |
| Long_4_8153 | 5 | 43.3699 | 1.199 | 236 | 236 | 304 | 304 | 114 | 114 | 234 | 234 | 189 | 189 | 90 | 92 | 107 | 107 | 141 | 143 | 99 | 108 | 247 | 269 | 199 | 199 |
| Long_4_8154 | 5 | 43.3699 | 1.199 | 236 | 236 | 291 | 296 | 120 | 120 | -9 | -9 | 188 | 188 | 90 | 92 | 107 | 108 | 141 | 143 | 83 | 99 | 246 | 269 | 199 | 199 |
| Long_4_8155 | 5 | 43.3699 | 1.199 | 236 | 236 | 296 | 304 | 120 | 120 | 234 | 234 | 189 | 191 | 92 | 94 | 107 | 114 | 141 | 143 | 99 | 99 | 247 | 269 | 199 | 199 |
| Long_4_8156 | 5 | 43.3699 | 1.199 | 236 | 236 | 296 | 306 | 120 | 120 | 223 | 240 | 191 | 191 | 92 | 92 | 108 | 114 | 141 | 143 | 83 | 99 | 246 | 247 | 199 | 199 |
| Pou_8109 | 6 | 43.4205 | 1.1753 | 236 | 236 | 304 | 306 | 114 | 120 | 234 | 234 | 189 | 189 | 90 | 96 | 107 | 108 | 143 | 143 | 99 | 108 | 265 | 269 | 199 | 199 |
| Pou_8110 | 6 | 43.4205 | 1.1753 | 236 | 236 | 296 | 306 | 114 | 120 | 223 | 238 | 189 | 189 | 90 | 92 | 107 | 111 | 143 | 143 | 99 | 99 | 246 | 269 | 199 | 199 |
| Pou_8111 | 6 | 43.4205 | 1.1753 | 236 | 236 | 306 | 306 | 114 | 120 | 223 | 234 | 189 | 191 | 90 | 96 | 107 | 108 | 141 | 143 | 83 | 108 | 246 | 269 | 199 | 199 |
| Pou_8112 | 6 | 43.4205 | 1.1753 | 236 | 239 | 296 | 304 | 114 | 120 | 223 | 238 | 189 | 191 | 96 | 96 | 107 | 108 | 141 | 141 | 99 | 99 | 247 | 247 | 199 | 199 |
| Pou_8113 | 6 | 43.4205 | 1.1753 | 236 | 236 | 296 | 304 | 120 | 120 | 223 | 234 | 189 | 191 | 92 | 96 | 107 | 108 | 141 | 141 | 83 | 99 | 247 | 247 | 199 | 199 |
| Pou_8114 | 6 | 43.4205 | 1.1753 | 236 | 239 | 296 | 304 | 114 | 120 | 223 | 238 | 189 | 189 | 90 | 90 | 107 | 108 | 141 | 143 | 99 | 99 | 247 | 247 | 199 | 199 |
| Pou_8115 | 6 | 43.4205 | 1.1753 | 236 | 239 | 304 | 304 | 120 | 120 | 234 | 240 | 189 | 191 | 90 | 92 | 107 | 111 | 141 | 143 | 83 | 99 | 247 | 247 | 199 | 199 |
| Pou_8116 | 6 | 43.4205 | 1.1753 | 236 | 236 | 296 | 304 | 114 | 120 | 223 | 223 | 189 | 189 | 90 | 96 | 108 | 108 | 141 | 143 | 99 | 99 | 246 | 247 | 199 | 199 |
| Pou_8117 | 6 | 43.4205 | 1.1753 | 236 | 236 | 304 | 306 | 120 | 120 | 223 | 234 | 189 | 191 | 90 | 92 | 108 | 114 | 143 | 176 | 83 | 83 | 246 | 269 | 199 | 199 |
| Pou_8118 | 6 | 43.4205 | 1.1753 | 236 | 236 | 306 | 306 | 120 | 120 | 223 | 234 | 189 | 191 | 90 | 92 | 107 | 114 | 141 | 143 | 83 | 108 | 265 | 269 | 199 | 199 |
| Pou_8119 | 6 | 43.4205 | 1.1753 | 236 | 236 | 296 | 304 | 120 | 120 | 234 | 234 | 189 | 189 | 90 | 92 | 107 | 114 | 141 | 143 | 83 | 99 | 246 | 269 | 199 | 199 |
| Pou_8120 | 6 | 43.4205 | 1.1753 | 236 | 236 | 291 | 296 | 120 | 120 | 234 | 240 | 189 | 191 | 90 | 92 | 107 | 114 | 141 | 143 | 99 | 108 | 246 | 247 | 199 | 199 |
| Pou_8157 | 6 | 43.4205 | 1.1753 | 236 | 236 | 304 | 306 | 120 | 120 | 234 | 234 | 189 | 189 | 90 | 92 | 107 | 114 | 143 | 143 | 99 | 108 | 246 | 265 | 199 | 199 |
| Pou_8158 | 6 | 43.4205 | 1.1753 | 236 | 236 | 296 | 304 | 120 | 120 | 240 | 240 | 189 | 189 | 92 | 92 | 107 | 107 | 141 | 141 | 99 | 99 | 265 | 269 | 199 | 199 |
| Pou_8159 | 6 | 43.4205 | 1.1753 | 236 | 236 | 304 | 306 | 114 | 120 | 234 | 234 | 189 | 191 | 90 | 92 | 107 | 114 | 141 | 143 | 83 | 108 | 265 | 269 | 199 | 199 |
| Pou_8160 | 6 | 43.4205 | 1.1753 | 236 | 236 | 304 | 306 | 120 | 120 | -9 | -9 | 189 | 189 | 90 | 90 | 107 | 107 | 141 | 143 | 83 | 99 | -9 | -9 | 199 | 199 |
| Pou_8161 | 6 | 43.4205 | 1.1753 | 236 | 236 | 304 | 306 | 114 | 120 | 234 | 234 | 189 | 189 | 90 | 92 | 107 | 108 | 141 | 176 | 83 | 83 | 246 | 265 | 199 | 199 |
| Pou_8162 | 6 | 43.4205 | 1.1753 | 239 | 239 | 300 | 308 | 120 | 120 | 234 | 234 | 188 | 189 | 92 | 96 | 107 | 107 | 143 | 143 | 99 | 108 | 246 | 247 | 199 | 199 |
| Pou_8163 | 6 | 43.4205 | 1.1753 | 236 | 236 | 296 | 306 | 114 | 120 | 234 | 234 | 191 | 191 | 90 | 92 | 107 | 107 | 141 | 143 | 83 | 99 | 265 | 269 | 199 | 199 |
| Pou_8164 | 6 | 43.4205 | 1.1753 | 236 | 236 | 304 | 304 | 120 | 120 | 223 | 234 | 189 | 191 | 90 | 92 | 107 | 108 | 143 | 143 | 83 | 83 | 246 | 269 | 199 | 199 |
| Pou_8165 | 6 | 43.4205 | 1.1753 | 236 | 236 | 296 | 304 | 114 | 114 | 240 | 240 | 189 | 191 | 90 | 90 | 107 | 108 | 143 | 143 | 99 | 99 | 246 | 265 | 199 | 199 |
| Pou_8166 | 6 | 43.4205 | 1.1753 | 236 | 236 | 296 | 306 | 120 | 120 | 223 | 234 | 189 | 191 | 90 | 90 | 107 | 108 | 143 | 143 | 83 | 83 | 246 | 269 | 199 | 199 |
| Pou_8167 | 6 | 43.4205 | 1.1753 | 236 | 236 | 296 | 304 | 120 | 120 | 223 | 234 | 189 | 189 | 90 | 92 | 107 | 114 | 141 | 143 | 83 | 99 | 246 | 269 | 199 | 199 |
| Pou_8168 | 6 | 43.4205 | 1.1753 | 236 | 236 | 296 | 310 | 120 | 120 | 223 | 234 | 189 | 191 | 90 | 92 | 107 | 114 | 143 | 143 | 83 | 99 | 246 | 247 | 199 | 199 |
| Cam_8169 | 7 | 43.4713 | 1.1792 | 236 | 236 | 296 | 304 | 120 | 120 | 223 | 234 | 189 | 191 | 90 | 90 | 107 | 111 | 141 | 143 | 83 | 99 | 269 | 269 | 199 | 199 |
| Cam_8170 | 7 | 43.4713 | 1.1792 | 239 | 239 | 294 | 300 | 120 | 120 | 238 | 238 | 188 | 189 | 92 | 94 | 107 | 114 | 141 | 141 | 99 | 99 | 247 | 269 | 199 | 199 |
| Cam_8172 | 7 | 43.4713 | 1.1792 | 236 | 236 | 298 | 298 | 114 | 114 | 234 | 234 | 189 | 191 | 90 | 90 | 107 | 108 | 141 | 143 | 99 | 99 | 247 | 265 | 199 | 199 |
| Cam_8173 | 7 | 43.4713 | 1.1792 | 236 | 236 | 306 | 306 | 114 | 120 | 234 | 238 | 189 | 191 | 90 | 90 | 107 | 108 | 141 | 179 | 83 | 99 | 247 | 269 | 199 | 199 |
| Cam_8175 | 7 | 43.4713 | 1.1792 | 236 | 236 | 291 | 306 | 120 | 120 | 234 | 238 | 189 | 189 | 90 | 92 | 107 | 107 | 141 | 141 | 83 | 99 | 246 | 269 | 199 | 199 |
| Cam_8176 | 7 | 43.4713 | 1.1792 | 236 | 236 | 296 | 304 | 120 | 120 | 234 | 240 | 189 | 191 | 92 | 92 | 107 | 107 | 141 | 143 | 108 | 108 | 246 | 269 | 199 | 199 |
| Cam_8177 | 7 | 43.4713 | 1.1792 | 236 | 239 | 296 | 304 | 120 | 120 | 223 | 223 | 189 | 191 | 92 | 92 | 107 | 107 | 141 | 176 | 83 | 83 | 246 | 269 | 199 | 199 |
| Cam_8178 | 7 | 43.4713 | 1.1792 | 236 | 236 | 304 | 306 | 120 | 120 | 223 | 238 | 189 | 191 | 90 | 92 | 107 | 107 | 141 | 141 | 83 | 99 | 246 | 269 | 199 | 199 |
| Cam_8179 | 7 | 43.4713 | 1.1792 | 236 | 236 | 304 | 306 | 114 | 120 | 234 | 234 | 189 | 189 | 90 | 90 | 107 | 107 | 143 | 143 | 99 | 99 | 246 | 246 | 199 | 199 |
| Cam_8180 | 7 | 43.4713 | 1.1792 | 236 | 236 | 291 | 306 | 120 | 120 | 238 | 238 | 188 | 189 | 90 | 90 | 107 | 107 | 141 | 143 | 99 | 99 | 246 | 265 | 199 | 199 |
| Cam_8121 | 7 | 43.4713 | 1.1792 | 236 | 236 | 306 | 306 | 114 | 114 | 223 | 234 | 189 | 191 | 90 | 92 | 108 | 111 | 141 | 141 | 83 | 99 | 246 | 247 | 199 | 199 |
| Cam_8122 | 7 | 43.4713 | 1.1792 | 236 | 236 | 306 | 306 | 120 | 120 | 223 | 234 | 189 | 191 | 92 | 92 | 107 | 107 | 143 | 143 | 99 | 99 | 247 | 269 | 199 | 199 |
| Cam_8123 | 7 | 43.4713 | 1.1792 | 236 | 236 | 296 | 304 | 114 | 120 | 223 | 234 | 189 | 191 | 92 | 94 | 107 | 108 | 141 | 143 | 83 | 83 | 269 | 269 | 199 | 199 |
| Cam_8124 | 7 | 43.4713 | 1.1792 | 236 | 236 | 296 | 304 | 120 | 120 | 234 | 234 | 189 | 189 | 90 | 92 | 107 | 108 | 141 | 141 | 99 | 99 | 247 | 247 | 199 | 199 |
| Cam_8125 | 7 | 43.4713 | 1.1792 | 236 | 236 | 304 | 304 | 120 | 120 | 223 | 223 | 189 | 189 | 92 | 92 | 107 | 108 | 139 | 141 | 99 | 99 | 247 | 247 | 199 | 199 |
| Cam_8126 | 7 | 43.4713 | 1.1792 | 236 | 239 | 296 | 296 | 120 | 120 | 234 | 240 | 189 | 189 | 90 | 90 | 107 | 107 | 141 | 143 | 99 | 99 | 247 | 269 | 199 | 199 |
| Cam_8128 | 7 | 43.4713 | 1.1792 | 236 | 236 | 291 | 306 | 120 | 120 | 234 | 238 | 189 | 191 | 90 | 92 | 107 | 107 | 141 | 143 | 83 | 83 | 247 | 269 | 199 | 199 |
| Cam_8129 | 7 | 43.4713 | 1.1792 | 236 | 239 | 291 | 304 | 114 | 120 | 234 | 234 | 189 | 189 | 90 | 90 | 107 | 107 | 141 | 141 | 83 | 99 | 247 | 269 | 199 | 199 |
| Cam_8130 | 7 | 43.4713 | 1.1792 | 236 | 239 | 304 | 304 | 120 | 120 | 234 | 234 | 189 | 189 | 90 | 90 | 107 | 108 | 141 | 143 | 99 | 108 | 247 | 247 | 199 | 199 |
| Cam_8131 | 7 | 43.4713 | 1.1792 | 236 | 236 | 304 | 304 | 120 | 120 | 234 | 234 | 191 | 191 | 92 | 92 | 108 | 114 | 141 | 143 | 83 | 108 | 247 | 247 | 199 | 199 |
| Cam_8132 | 7 | 43.4713 | 1.1792 | 239 | 239 | 300 | 306 | 120 | 120 | 234 | 240 | 189 | 189 | 90 | 92 | 107 | 107 | 143 | 143 | 83 | 99 | 246 | 269 | 199 | 199 |
| Lav_3637 | 8 | 44.78 | 1.75 | 236 | 236 | 308 | 310 | 120 | 120 | 234 | 238 | 189 | 189 | 96 | 96 | 107 | 107 | 139 | 143 | 83 | 83 | 246 | 269 | 199 | 199 |
| Lav_3638 | 8 | 44.78 | 1.75 | 236 | 236 | 304 | 304 | 114 | 120 | 223 | 238 | 191 | 191 | 92 | 92 | 107 | 111 | 141 | 143 | 83 | 99 | 246 | 269 | 199 | 199 |
| AuS_6758 | 9 | 44.569 | 4.698 | 236 | 236 | 296 | 315 | 120 | 123 | 223 | 234 | 189 | 191 | 90 | 90 | 107 | 107 | 143 | 143 | 99 | 99 | 247 | 247 | 199 | 199 |
| AuS_6759 | 9 | 44.569 | 4.698 | 236 | 236 | 296 | 315 | 114 | 120 | 223 | 238 | 191 | 191 | 90 | 92 | 107 | 108 | 143 | 143 | 99 | 99 | 246 | 246 | 199 | 199 |
| AuS_6760 | 9 | 44.569 | 4.698 | 236 | 236 | 296 | 315 | 120 | 123 | 223 | 238 | 191 | 191 | 90 | 92 | 107 | 107 | 143 | 143 | 99 | 99 | 247 | 247 | 199 | 199 |
| AuS_6761 | 9 | 44.569 | 4.698 | 236 | 236 | 296 | 304 | 114 | 120 | 238 | 240 | 191 | 191 | 90 | 90 | 107 | 107 | 143 | 143 | 99 | 99 | 247 | 247 | 199 | 199 |
| Ar2T_6677 | 10 | 43.493 | 4.714 | 236 | 236 | 296 | 306 | 114 | 120 | 223 | 234 | 189 | 191 | 90 | 90 | 107 | 108 | 143 | 143 | 99 | 99 | 246 | 265 | 199 | 199 |
| Ar2T_6678 | 10 | 43.493 | 4.714 | 236 | 236 | 296 | 306 | 120 | 120 | 223 | 238 | 188 | 189 | 90 | 90 | 107 | 108 | 141 | 141 | 83 | 99 | 246 | 265 | 199 | 199 |
| Ar2T_6679 | 10 | 43.493 | 4.714 | 236 | 236 | 306 | 306 | 120 | 123 | 234 | 238 | 189 | 189 | 90 | 90 | 107 | 108 | 141 | 143 | 99 | 99 | 246 | 265 | 199 | 199 |
| Ar2T_6680 | 10 | 43.493 | 4.714 | 236 | 236 | 306 | 306 | 114 | 120 | 234 | 238 | 189 | 191 | 90 | 90 | 107 | 108 | 143 | 143 | 99 | 99 | 246 | 265 | 199 | 199 |
| Ar3T_6681 | 11 | 43.493 | 4.714 | 236 | 236 | 296 | 306 | 120 | 120 | 223 | 234 | 189 | 191 | 90 | 90 | 107 | 107 | 143 | 143 | 83 | 108 | 246 | 269 | 199 | 199 |
| Ar3T_6682 | 11 | 43.493 | 4.714 | 236 | 236 | 296 | 306 | 120 | 120 | 223 | 234 | 188 | 189 | 90 | 90 | 107 | 114 | 143 | 143 | 83 | 108 | 246 | 246 | 199 | 199 |
| Ar3T_6683 | 11 | 43.493 | 4.714 | 236 | 236 | 306 | 313 | 114 | 120 | 234 | 238 | 188 | 189 | 92 | 92 | 105 | 107 | 141 | 141 | 99 | 99 | 265 | 265 | 199 | 199 |
| Ar3T_6684 | 11 | 43.493 | 4.714 | 236 | 236 | 296 | 306 | 120 | 123 | 234 | 234 | 188 | 191 | 90 | 90 | 107 | 107 | 139 | 143 | 83 | 99 | 246 | 269 | 199 | 199 |
| Ar3T_6685 | 11 | 43.493 | 4.714 | 236 | 236 | 296 | 306 | 120 | 123 | 223 | 234 | 188 | 188 | 90 | 90 | 108 | 108 | 139 | 139 | 83 | 83 | 246 | 246 | 199 | 199 |
| Ar3T_6686 | 11 | 43.493 | 4.714 | 236 | 236 | 296 | 306 | 120 | 120 | 234 | 240 | 188 | 191 | 90 | 90 | 107 | 107 | 139 | 143 | 83 | 99 | 246 | 246 | 199 | 199 |
| Ar3T_6687 | 11 | 43.493 | 4.714 | 236 | 236 | 296 | 306 | 120 | 123 | 234 | 234 | 189 | 191 | 90 | 90 | 107 | 107 | 143 | 143 | 83 | 108 | 246 | 246 | 199 | 199 |
| Ar3T_6688 | 11 | 43.493 | 4.714 | 236 | 236 | 296 | 306 | 120 | 120 | 234 | 234 | 188 | 191 | 90 | 90 | 107 | 107 | 139 | 143 | 83 | 99 | 246 | 269 | 199 | 199 |
| Ar3T_6689 | 11 | 43.493 | 4.714 | 236 | 236 | -9 | -9 | 120 | 120 | 223 | 240 | 188 | 188 | 90 | 90 | 107 | 114 | 139 | 139 | 83 | 99 | 246 | 246 | 199 | 199 |
| Ar3T_6690 | 11 | 43.493 | 4.714 | 236 | 236 | 296 | 306 | 120 | 120 | 234 | 240 | 188 | 189 | 90 | 90 | 107 | 114 | 143 | 143 | 83 | 108 | 246 | 269 | 199 | 199 |
| Ar3T_6928 | 11 | 43.493 | 4.714 | 236 | 239 | 304 | 306 | 114 | 120 | 234 | 234 | 189 | 191 | 90 | 90 | 107 | 114 | 139 | 143 | 99 | 99 | 246 | 265 | 199 | 199 |
| Ar3T_6929 | 11 | 43.493 | 4.714 | 236 | 236 | 296 | 304 | 120 | 123 | 234 | 238 | 188 | 189 | 90 | 92 | 107 | 107 | 141 | 143 | 99 | 99 | 246 | 246 | -9 | -9 |
| Ar3T_6930 | 11 | 43.493 | 4.714 | 236 | 236 | 296 | 296 | 120 | 120 | 234 | 234 | 189 | 189 | 90 | 96 | 107 | 107 | 143 | 143 | 99 | 108 | 246 | 246 | 199 | 199 |
| Ar3T_6931 | 11 | 43.493 | 4.714 | 236 | 236 | 291 | 306 | 114 | 120 | 223 | 234 | 188 | 189 | 90 | 90 | 107 | 108 | 141 | 143 | 99 | 99 | 265 | 265 | 199 | 199 |
| Ar3T_6932 | 11 | 43.493 | 4.714 | 236 | 236 | 304 | 306 | 120 | 120 | 234 | 234 | 188 | 188 | 90 | 90 | 107 | 107 | 141 | 141 | 99 | 99 | 265 | 269 | 199 | 199 |
| Ar3T_6933 | 11 | 43.493 | 4.714 | 236 | 239 | -9 | -9 | 120 | 120 | 234 | 240 | 188 | 191 | 90 | 90 | 105 | 107 | 143 | 143 | 83 | 99 | 246 | 265 | 199 | 199 |
| Ar3T_6934 | 11 | 43.493 | 4.714 | 236 | 236 | 304 | 313 | 120 | 120 | 234 | 234 | 188 | 189 | 92 | 96 | 107 | 107 | 143 | 143 | 99 | 99 | 246 | 246 | 199 | 199 |
| Ar3T_6935 | 11 | 43.493 | 4.714 | 236 | 236 | 296 | 306 | 120 | 120 | 234 | 234 | 188 | 189 | 90 | 90 | 107 | 114 | 143 | 143 | 83 | 108 | 246 | 246 | 199 | 199 |
| Ar3T_6936 | 11 | 43.493 | 4.714 | 221 | 236 | 304 | 306 | 120 | 120 | 223 | 234 | 189 | 189 | 90 | 92 | 107 | 107 | 141 | 143 | 99 | 99 | 246 | 265 | 199 | 199 |
| Ar3T_6937 | 11 | 43.493 | 4.714 | 236 | 236 | 296 | 306 | 120 | 123 | 223 | 234 | 188 | 191 | 90 | 90 | 107 | 114 | 139 | 143 | 83 | 99 | 246 | 269 | 199 | 199 |
| Ar3T_6938 | 11 | 43.493 | 4.714 | 236 | 236 | 296 | 306 | 120 | 120 | 223 | 240 | 188 | 189 | 90 | 90 | 107 | 107 | 143 | 143 | 83 | 108 | 246 | 269 | 199 | 199 |
| Ar3T_6939 | 11 | 43.493 | 4.714 | 236 | 236 | 296 | 306 | 120 | 120 | 223 | 234 | 189 | 191 | 90 | 90 | 107 | 107 | 143 | 143 | 83 | 108 | 246 | 246 | 199 | 199 |
| Ar3T_6940 | 11 | 43.493 | 4.714 | 236 | 236 | -9 | -9 | 120 | 123 | 234 | 234 | 189 | 191 | 90 | 90 | 107 | 107 | 143 | 143 | 83 | 108 | 246 | 269 | 199 | 199 |
| Ar3T_6941 | 11 | 43.493 | 4.714 | 236 | 236 | 296 | 304 | 114 | 123 | 234 | 238 | 188 | 189 | 90 | 90 | 107 | 107 | 141 | 141 | 99 | 99 | 246 | 247 | 199 | 199 |
| Ar3T_6942 | 11 | 43.493 | 4.714 | 236 | 236 | 296 | 304 | 120 | 120 | 234 | 240 | 188 | 189 | 96 | 96 | 105 | 107 | 143 | 143 | 99 | 99 | 246 | 246 | 199 | 199 |
| Ar3T_6943 | 11 | 43.493 | 4.714 | 236 | 236 | 296 | 304 | 120 | 120 | 234 | 234 | 189 | 189 | 90 | 92 | 107 | 107 | 143 | 143 | 99 | 108 | 246 | 265 | 199 | 199 |
| Ar3T_6944 | 11 | 43.493 | 4.714 | 236 | 236 | 304 | 306 | 120 | 120 | 234 | 234 | 189 | 191 | 90 | 92 | 107 | 108 | 141 | 143 | 83 | 89 | 246 | 246 | 199 | 199 |
| Ar3T_6945 | 11 | 43.493 | 4.714 | 239 | 239 | 296 | 306 | 120 | 120 | 223 | 238 | 189 | 189 | 90 | 92 | 105 | 107 | 141 | 143 | 99 | 99 | 246 | 246 | 199 | 199 |
| Ar3T_6946 | 11 | 43.493 | 4.714 | 236 | 236 | 296 | 306 | 120 | 120 | 223 | 240 | 189 | 191 | 90 | 90 | 107 | 114 | 143 | 143 | 83 | 108 | 246 | 246 | 199 | 199 |
| Ar3T_6947 | 11 | 43.493 | 4.714 | 236 | 236 | 291 | 296 | 120 | 120 | 234 | 234 | 189 | 189 | 96 | 96 | 107 | 107 | 141 | 141 | 99 | 99 | -9 | -9 | 199 | 199 |
| Ar3T_6948 | 11 | 43.493 | 4.714 | 236 | 236 | 296 | 306 | 120 | 123 | 223 | 240 | 188 | 188 | 90 | 90 | 107 | 107 | 143 | 143 | 83 | 108 | 246 | 246 | 199 | 199 |
| Ar3T_6949 | 11 | 43.493 | 4.714 | 236 | 236 | 296 | 306 | 120 | 120 | 234 | 234 | 188 | 191 | 90 | 90 | 107 | 107 | 139 | 143 | 83 | 99 | 246 | 269 | 199 | 199 |
| Ar3T_6950 | 11 | 43.493 | 4.714 | 236 | 236 | 296 | 306 | 120 | 120 | 223 | 234 | 188 | 189 | 90 | 90 | 107 | 114 | 143 | 143 | 83 | 108 | 246 | 269 | 199 | 199 |
| Ar4T_6691 | 12 | 43.492 | 4.715 | 236 | 236 | 291 | 306 | -9 | -9 | 223 | 234 | 188 | 189 | 92 | 92 | 107 | 108 | 141 | 141 | 99 | 99 | 269 | 269 | 199 | 199 |
| Ar4T_6692 | 12 | 43.492 | 4.715 | 236 | 236 | 291 | 306 | -9 | -9 | 223 | 234 | 188 | 189 | 92 | 96 | 105 | 107 | 141 | 141 | 99 | 99 | 265 | 265 | 199 | 199 |
| Ar4T_6693 | 12 | 43.492 | 4.715 | 236 | 236 | 291 | 306 | 114 | 120 | 223 | 234 | 189 | 189 | 90 | 96 | 105 | 107 | 143 | 143 | 99 | 99 | 265 | 265 | 199 | 199 |
| Ar4T_6694 | 12 | 43.492 | 4.715 | 236 | 236 | 291 | 291 | -9 | -9 | 223 | 234 | 189 | 189 | 90 | 96 | 105 | 107 | 141 | 143 | 99 | 99 | 269 | 269 | 199 | 199 |
| Ar4T_6695 | 12 | 43.492 | 4.715 | 236 | 236 | 306 | 313 | -9 | -9 | 234 | 238 | 189 | 189 | 90 | 96 | 107 | 108 | 141 | 143 | 99 | 99 | 265 | 265 | 199 | 199 |
| Ar4T_6696 | 12 | 43.492 | 4.715 | 236 | 236 | 291 | 306 | -9 | -9 | 223 | 234 | 189 | 189 | 90 | 96 | 105 | 107 | 141 | 143 | 99 | 99 | 269 | 269 | 199 | 199 |
| Ar4T_6697 | 12 | 43.492 | 4.715 | 236 | 236 | 306 | 313 | 114 | 120 | 234 | 238 | 189 | 189 | 90 | 96 | 105 | 107 | 143 | 143 | 99 | 99 | 269 | 269 | 199 | 199 |
| Ar4T_6698 | 12 | 43.492 | 4.715 | 236 | 236 | 306 | 313 | 114 | 120 | 234 | 238 | 188 | 189 | 90 | 96 | 105 | 107 | 141 | 141 | 99 | 99 | 269 | 269 | 199 | 199 |
| Ar4T_6699 | 12 | 43.492 | 4.715 | 236 | 236 | 291 | 306 | 114 | 120 | 223 | 234 | 189 | 189 | 92 | 92 | 107 | 108 | 143 | 143 | 99 | 99 | 269 | 269 | 199 | 199 |
| Ar4T_6700 | 12 | 43.492 | 4.715 | 236 | 236 | 306 | 313 | 114 | 120 | 234 | 238 | 188 | 189 | 90 | 90 | 107 | 108 | 141 | 143 | 99 | 99 | 265 | 265 | 199 | 199 |
| Ar4T_6951 | 12 | 43.492 | 4.715 | 236 | 236 | 291 | 306 | 114 | 120 | 223 | 234 | 188 | 189 | 92 | 96 | 105 | 107 | 141 | 141 | 99 | 99 | 265 | 265 | 199 | 199 |
| Ar4T_6952 | 12 | 43.492 | 4.715 | 236 | 236 | 306 | 313 | 114 | 120 | 234 | 238 | 188 | 189 | 92 | 92 | 107 | 108 | 141 | 141 | 99 | 99 | 269 | 269 | 199 | 199 |
| Ar4T_6953 | 12 | 43.492 | 4.715 | 236 | 236 | 306 | 313 | 114 | 120 | 234 | 238 | 188 | 189 | 92 | 92 | 105 | 107 | 141 | 143 | 99 | 99 | 265 | 265 | 199 | 199 |
| Ar4T_6954 | 12 | 43.492 | 4.715 | 236 | 236 | 306 | 313 | 114 | 120 | 234 | 238 | 189 | 189 | 90 | 90 | 107 | 108 | 143 | 143 | 99 | 99 | 269 | 269 | 199 | 199 |
| Ar4T_6955 | 12 | 43.492 | 4.715 | 236 | 236 | 291 | 306 | 114 | 120 | 223 | 234 | 189 | 189 | 92 | 96 | 107 | 108 | 143 | 143 | 99 | 99 | 269 | 269 | 199 | 199 |
| Ar4T_6956 | 12 | 43.492 | 4.715 | 236 | 236 | 306 | 313 | 114 | 120 | 223 | 234 | 189 | 189 | 90 | 90 | 107 | 108 | 143 | 143 | 99 | 99 | 269 | 269 | 199 | 199 |
| Ar4T_6957 | 12 | 43.492 | 4.715 | 236 | 236 | 291 | 306 | 114 | 120 | 223 | 234 | 188 | 189 | 92 | 92 | 105 | 107 | 141 | 143 | 99 | 99 | 269 | 269 | 199 | 199 |
| Ar4T_6959 | 12 | 43.492 | 4.715 | 236 | 236 | 291 | 306 | 114 | 120 | 223 | 234 | 189 | 189 | 92 | 92 | 107 | 108 | 141 | 143 | 99 | 99 | 265 | 265 | 199 | 199 |
| Ar4T_6960 | 12 | 43.492 | 4.715 | 236 | 236 | 306 | 313 | 114 | 120 | 234 | 238 | 188 | 189 | 92 | 96 | 105 | 107 | 141 | 141 | 99 | 99 | 269 | 269 | 199 | 199 |
| Ar4T_6962 | 12 | 43.492 | 4.715 | 236 | 236 | 306 | 313 | 114 | 120 | 234 | 238 | 188 | 189 | 92 | 96 | 107 | 108 | 141 | 141 | 99 | 99 | 269 | 269 | 199 | 199 |
| Ar4T_6963 | 12 | 43.492 | 4.715 | 236 | 236 | 306 | 313 | 114 | 120 | 234 | 238 | 189 | 189 | 90 | 90 | 105 | 107 | 143 | 143 | 99 | 99 | 269 | 269 | 199 | 199 |
| Ar4T_6964 | 12 | 43.492 | 4.715 | 236 | 236 | 291 | 306 | 114 | 120 | 223 | 234 | 189 | 189 | 90 | 90 | 107 | 108 | 143 | 143 | 99 | 99 | 269 | 269 | 199 | 199 |
| Ar4T_6965 | 12 | 43.492 | 4.715 | 236 | 236 | 291 | 313 | 120 | 123 | 223 | 223 | 189 | 189 | 90 | 96 | 107 | 111 | 143 | 143 | 99 | 99 | 247 | 265 | 199 | 226 |
| Ar4T_6968 | 12 | 43.492 | 4.715 | 236 | 236 | 306 | 313 | 114 | 120 | 234 | 238 | 189 | 189 | 92 | 96 | 107 | 108 | 141 | 143 | 99 | 99 | 269 | 269 | 199 | 199 |
| Ar4T_6969 | 12 | 43.492 | 4.715 | 236 | 236 | 291 | 306 | 114 | 120 | 223 | 234 | 188 | 189 | 92 | 96 | 105 | 107 | 144 | 143 | 99 | 99 | 269 | 269 | 199 | 199 |
| Ar4T_6970 | 12 | 43.492 | 4.715 | 236 | 236 | 306 | 313 | 114 | 120 | 234 | 238 | 189 | 189 | 90 | 90 | 105 | 107 | 143 | 143 | 99 | 99 | 269 | 269 | 199 | 199 |
| Ar4T_6971 | 12 | 43.492 | 4.715 | 236 | 236 | 306 | 313 | 114 | 120 | 234 | 238 | 188 | 189 | 90 | 90 | 105 | 107 | 141 | 141 | 99 | 99 | 265 | 265 | 199 | 199 |
| Ar4T_6972 | 12 | 43.492 | 4.715 | 236 | 236 | 306 | 313 | 114 | 120 | 234 | 238 | 188 | 189 | 90 | 96 | 105 | 107 | 141 | 143 | 99 | 99 | 265 | 265 | 199 | 199 |
| Ar4T_6973 | 12 | 43.492 | 4.715 | 236 | 236 | 306 | 313 | 114 | 120 | 223 | 234 | 189 | 189 | 92 | 96 | 107 | 108 | 143 | 143 | 99 | 99 | 269 | 269 | 199 | 199 |
| Ar6T_6701 | 13 | 43.453 | 4.741 | 236 | 236 | 304 | 313 | 120 | 120 | 234 | 234 | 191 | 191 | 85 | 92 | 107 | 108 | 139 | 139 | 83 | 99 | 265 | 265 | 199 | 199 |
| Ar7T_6702 | 14 | 43.452 | 4.737 | 228 | 228 | 303 | 304 | 120 | 120 | 234 | 238 | 189 | 189 | 92 | 92 | 107 | 111 | 143 | 143 | 83 | 99 | 265 | 269 | 199 | 199 |
| Ar7T_6703 | 14 | 43.452 | 4.737 | 236 | 236 | 303 | 306 | 120 | 120 | 234 | 238 | 189 | 189 | -9 | -9 | 107 | 111 | 143 | 143 | 83 | 99 | 265 | 269 | 199 | 199 |
| Ar7T_6704 | 14 | 43.452 | 4.737 | 236 | 236 | 296 | 303 | 120 | 120 | 223 | 234 | 188 | 189 | 90 | 92 | 107 | 114 | 141 | 143 | 89 | 89 | 246 | 265 | 199 | 199 |
| Ar7T_6705 | 14 | 43.452 | 4.737 | 228 | 228 | 306 | 313 | 120 | 120 | 234 | 238 | 189 | 189 | 90 | 92 | 107 | 107 | 143 | 143 | 99 | 108 | 265 | 265 | 199 | 199 |
| Ar7T_6706 | 14 | 43.452 | 4.737 | 236 | 236 | 303 | 306 | 120 | 120 | 234 | 238 | 189 | 191 | -9 | -9 | 107 | 111 | 141 | 143 | 83 | 108 | 265 | 269 | 199 | 199 |
| Ar7T_6707 | 14 | 43.452 | 4.737 | 236 | 236 | 304 | 306 | 120 | 120 | 223 | 234 | 189 | 189 | 96 | 96 | 107 | 107 | 141 | 176 | 83 | 99 | 246 | 265 | 199 | 199 |
| Ar7T_6708 | 14 | 43.452 | 4.737 | 228 | 228 | 304 | 313 | 120 | 120 | 234 | 238 | 189 | 191 | 92 | 92 | 107 | 107 | 141 | 141 | 83 | 108 | 265 | 265 | 199 | 199 |
| Ar7T_6710 | 14 | 43.452 | 4.737 | 236 | 236 | 303 | 304 | 114 | 120 | 234 | 238 | 189 | 189 | 92 | 92 | 107 | 107 | 143 | 143 | 99 | 108 | 265 | 269 | 199 | 199 |
| Ar7T_6711 | 14 | 43.452 | 4.737 | 236 | 236 | 303 | 304 | 120 | 120 | 234 | 238 | 189 | 191 | 90 | 92 | 107 | 111 | 141 | 143 | 83 | 108 | 265 | 265 | 199 | 199 |
| Ar7T_6974 | 14 | 43.452 | 4.737 | 236 | 236 | 306 | 306 | 120 | 120 | 234 | 234 | 189 | 189 | 90 | 90 | 107 | 107 | 141 | 143 | 99 | 99 | 246 | 269 | 199 | 199 |
| Ar7T_6975 | 14 | 43.452 | 4.737 | 236 | 236 | 304 | 304 | 114 | 120 | 234 | 238 | 189 | 189 | 90 | 92 | 107 | 114 | 141 | 143 | 99 | 99 | 265 | 269 | 199 | 199 |
| Ar7T_6976 | 14 | 43.452 | 4.737 | 236 | 236 | 296 | 303 | 120 | 120 | 223 | 234 | 188 | 189 | 90 | 92 | 107 | 107 | 141 | 143 | 89 | 89 | 265 | 265 | 199 | 199 |
| Ar7T_6977 | 14 | 43.452 | 4.737 | 236 | 236 | 296 | 296 | 120 | 120 | 234 | 234 | 188 | 189 | 90 | 90 | 107 | 107 | 141 | 143 | 99 | 99 | 246 | 246 | 199 | 199 |
| Ar7T_6978 | 14 | 43.452 | 4.737 | 236 | 236 | 296 | 304 | 114 | 120 | 223 | 238 | 189 | 189 | 90 | 92 | 107 | 107 | 143 | 143 | 99 | 99 | 265 | 269 | 199 | 199 |
| Ar7T_6979 | 14 | 43.452 | 4.737 | 236 | 236 | 304 | 313 | 120 | 120 | 234 | 238 | 189 | 191 | 92 | 92 | 107 | 111 | 141 | 143 | 83 | 108 | 265 | 265 | 199 | 199 |
| Ar7T_6980 | 14 | 43.452 | 4.737 | 236 | 236 | 296 | 303 | 120 | 120 | 223 | 234 | 188 | 189 | 90 | 92 | 107 | 114 | 141 | 143 | 89 | 89 | 246 | 265 | 199 | 199 |
| Ar7T_6981 | 14 | 43.452 | 4.737 | 236 | 236 | 296 | 313 | 114 | 123 | 223 | 234 | 189 | 189 | 90 | 90 | 107 | 114 | 141 | 143 | 99 | 99 | 265 | 269 | 199 | 199 |
| Ar7T_6982 | 14 | 43.452 | 4.737 | 236 | 236 | 296 | 304 | 123 | 123 | 234 | 240 | 188 | 191 | 92 | 92 | 107 | 108 | 143 | 143 | 83 | 83 | 246 | 246 | 199 | 199 |
| Ar7T_6983 | 14 | 43.452 | 4.737 | 238 | 238 | 300 | 300 | 120 | 120 | 234 | 234 | 189 | 189 | 90 | 96 | 107 | 107 | 139 | 141 | 83 | 99 | 266 | 269 | 199 | 199 |
| Ar8T_6713 | 15 | 43.611 | 4.605 | 236 | 236 | 296 | 306 | 114 | 120 | 223 | 234 | 188 | 189 | 90 | 94 | 107 | 107 | 141 | 141 | 99 | 99 | 265 | 265 | 199 | 199 |
| Ar8T_6714 | 15 | 43.611 | 4.605 | 236 | 236 | 296 | 306 | 120 | 120 | 223 | 234 | 189 | 191 | 90 | 94 | 107 | 107 | 139 | 141 | 99 | 99 | 247 | 265 | 199 | 199 |
| Ar8T_6715 | 15 | 43.611 | 4.605 | 236 | 239 | 303 | 310 | 120 | 120 | 223 | 240 | 188 | 189 | 92 | 92 | 107 | 107 | 139 | 141 | 83 | 99 | 247 | 265 | 199 | 199 |
| Ar8T_6716 | 15 | 43.611 | 4.605 | 236 | 239 | 304 | 310 | 120 | 120 | 234 | 240 | 188 | 189 | 90 | 92 | 107 | 107 | 139 | 143 | 83 | 83 | 269 | 269 | 199 | 199 |
| Ar8T_6717 | 15 | 43.611 | 4.605 | 236 | 239 | 304 | 313 | 120 | 120 | 234 | 234 | 188 | 189 | 92 | 96 | 107 | 107 | 141 | 143 | -9 | -9 | 247 | 265 | 199 | 199 |
| Ar8T_6718 | 15 | 43.611 | 4.605 | 236 | 239 | 303 | 310 | 120 | 120 | 223 | 240 | 188 | 189 | 90 | 96 | 107 | 107 | 139 | 143 | 83 | 83 | 265 | 269 | 199 | 199 |
| Ar8T_6719 | 15 | 43.611 | 4.605 | 236 | 239 | 303 | 310 | 120 | 123 | 223 | 240 | 188 | 189 | 90 | 92 | 107 | 107 | 139 | 143 | 83 | 83 | 247 | 265 | 199 | 199 |
| Ar8T_6720 | 15 | 43.611 | 4.605 | 236 | 236 | 296 | 306 | 120 | 120 | 223 | 234 | 188 | 189 | 90 | 94 | 107 | 107 | 141 | 141 | 99 | 99 | 247 | 265 | 199 | 199 |
| Ar9T_6712 | 16 | 43.622 | 4.589 | 236 | 236 | 291 | 304 | 120 | 120 | 234 | 238 | 189 | 189 | 90 | 92 | 107 | 107 | 141 | 143 | 99 | 99 | 246 | 246 | 199 | 199 |
| Ar12S_6581 | 17 | 43.59 | 4.438 | 236 | 236 | 306 | 306 | 120 | 120 | 234 | 238 | 188 | 188 | 90 | 92 | 107 | 107 | 143 | 143 | 108 | 108 | 246 | 247 | 199 | 199 |
| Ar12S_6582 | 17 | 43.59 | 4.438 | 236 | 236 | 306 | 306 | 120 | 120 | 234 | 238 | 188 | 189 | 92 | 96 | 107 | 107 | 141 | 143 | 99 | 108 | 265 | 269 | 199 | 199 |
| Ar12S_6583 | 17 | 43.59 | 4.438 | 236 | 236 | 304 | 306 | 120 | 120 | 223 | 234 | 188 | 191 | 90 | 90 | 108 | 108 | 141 | 141 | 99 | 99 | 246 | 246 | 199 | 199 |
| Ar12S_6584 | 17 | 43.59 | 4.438 | 236 | 236 | 296 | 306 | 114 | 120 | 234 | 234 | 189 | 189 | 92 | 96 | 107 | 107 | 141 | 143 | 99 | 99 | 246 | 247 | 199 | 199 |
| Ar12S_6585 | 17 | 43.59 | 4.438 | 236 | 236 | 306 | 306 | 120 | 120 | 234 | 238 | 189 | 189 | 90 | 92 | 107 | 107 | 141 | 141 | 99 | 99 | 247 | 265 | 199 | 199 |
| Ar12S_6586 | 17 | 43.59 | 4.438 | 236 | 236 | 296 | 306 | 114 | 123 | 234 | 234 | 188 | 189 | 92 | 96 | 107 | 107 | 141 | 143 | 99 | 99 | 265 | 269 | 199 | 199 |
| Ar12S_6587 | 17 | 43.59 | 4.438 | 239 | 239 | 304 | 304 | 120 | 123 | 234 | 240 | 189 | 191 | 92 | 92 | 107 | 107 | 143 | 143 | 83 | 99 | 246 | 246 | 199 | 199 |
| Ar12S_6588 | 17 | 43.59 | 4.438 | 236 | 236 | 306 | 315 | 120 | 123 | 234 | 234 | 188 | 189 | 90 | 90 | 107 | 107 | 143 | 143 | 83 | 108 | 246 | 265 | 199 | 199 |
| Ar12S_6589 | 17 | 43.59 | 4.438 | 236 | 236 | 306 | 306 | 120 | 120 | 234 | 234 | 188 | 189 | 90 | 96 | 107 | 107 | 141 | 143 | 99 | 108 | 246 | 247 | 199 | 199 |
| Ar12S_6590 | 17 | 43.59 | 4.438 | 236 | 239 | 296 | 296 | 120 | 120 | 223 | 238 | 191 | 191 | 90 | 92 | 107 | 108 | 141 | 143 | 97 | 97 | 265 | 269 | 199 | 199 |
| Ar12S_6591 | 17 | 43.59 | 4.438 | 236 | 236 | 304 | 306 | 120 | 123 | 234 | 234 | 189 | 189 | 90 | 90 | 107 | 107 | 141 | 143 | 83 | 99 | 265 | 269 | 199 | 199 |
| Ar12S_6592 | 17 | 43.59 | 4.438 | 236 | 236 | 304 | 304 | 120 | 120 | 234 | 240 | 191 | 191 | -9 | -9 | 106 | 107 | 141 | 141 | 99 | 99 | 265 | 269 | 199 | 199 |
| Ar12S_6593 | 17 | 43.59 | 4.438 | 236 | 236 | 306 | 315 | 120 | 123 | 234 | 234 | 188 | 191 | 90 | 90 | 107 | 107 | 143 | 143 | 83 | 108 | 265 | 265 | 199 | 199 |
| Ar12S_6594 | 17 | 43.59 | 4.438 | 236 | 236 | 304 | 306 | 120 | 123 | 234 | 234 | 189 | 191 | 90 | 90 | 107 | 108 | 141 | 141 | 99 | 99 | 247 | 247 | 199 | 199 |
| Ar12S_6595 | 17 | 43.59 | 4.438 | 236 | 236 | 306 | 313 | 120 | 120 | 234 | 234 | 189 | 189 | 90 | 90 | 107 | 108 | 141 | 141 | 89 | 99 | 247 | 248 | 199 | 199 |
| Ar12S_6596 | 17 | 43.59 | 4.438 | 236 | 236 | 296 | 306 | 114 | 120 | 223 | 234 | 189 | 189 | 92 | 92 | 107 | 107 | 141 | 143 | 99 | 99 | 247 | 265 | 199 | 199 |
| Ar12S_6597 | 17 | 43.59 | 4.438 | 236 | 236 | 306 | 306 | 114 | 114 | 234 | 240 | 189 | 189 | 90 | 92 | 107 | 114 | 141 | 141 | 99 | 108 | 246 | 246 | 199 | 199 |
| Ar12S_6598 | 17 | 43.59 | 4.438 | 236 | 236 | 296 | 306 | 120 | 123 | 234 | 234 | 189 | 189 | 90 | 92 | 107 | 107 | 141 | 143 | 99 | 99 | 265 | 269 | 199 | 199 |
| Ar12S_6599 | 17 | 43.59 | 4.438 | 236 | 236 | 306 | 306 | 120 | 123 | 223 | 234 | 189 | 189 | 90 | 96 | 107 | 107 | 141 | 141 | 89 | 99 | 246 | 269 | 199 | 199 |
| Ar12S_6600 | 17 | 43.59 | 4.438 | 236 | 236 | 306 | 306 | 120 | 120 | 234 | 238 | 188 | 188 | 90 | 92 | 107 | 107 | 143 | 143 | 108 | 108 | 247 | 265 | 199 | 199 |
| Ar12S_6601 | 17 | 43.59 | 4.438 | 236 | 239 | 296 | 306 | 120 | 120 | 234 | 240 | 191 | 191 | 90 | 92 | 107 | 108 | 141 | 143 | 99 | 99 | 247 | 265 | 199 | 199 |
| Ar12S_6602 | 17 | 43.59 | 4.438 | 236 | 236 | 296 | 304 | 120 | 120 | 223 | 238 | 189 | 189 | 90 | 92 | 107 | 107 | 143 | 143 | 99 | 108 | 265 | 269 | 199 | 226 |
| Ar12S_6603 | 17 | 43.59 | 4.438 | 236 | 239 | 304 | 306 | 120 | 123 | 223 | 234 | 189 | 191 | 90 | 90 | 107 | 109 | 141 | 141 | 99 | 99 | 265 | 269 | 199 | 199 |
| Ar12S_6604 | 17 | 43.59 | 4.438 | 236 | 236 | 306 | 306 | 120 | 120 | 234 | 238 | 188 | 189 | 90 | 96 | 107 | 107 | -9 | -9 | 99 | 99 | 247 | 265 | 199 | 199 |
| Ar12S_6605 | 17 | 43.59 | 4.438 | 239 | 239 | 291 | 306 | 120 | 120 | 234 | 234 | 188 | 188 | 90 | 90 | 108 | 108 | 139 | 141 | 83 | 99 | 246 | 265 | 199 | 199 |
| Ar12S_6606 | 17 | 43.59 | 4.438 | 236 | 236 | 306 | 313 | 114 | 120 | 234 | 234 | 189 | 189 | 90 | 92 | 107 | 108 | 141 | 143 | 99 | 99 | 246 | 247 | 199 | 199 |
| Ar12S_6607 | 17 | 43.59 | 4.438 | 236 | 239 | 296 | 306 | 114 | 120 | 223 | 234 | 189 | 189 | 90 | 90 | 107 | 108 | 141 | 141 | 89 | 99 | 246 | 265 | 199 | 199 |
| Ar12S_6608 | 17 | 43.59 | 4.438 | 236 | 239 | 304 | 304 | 114 | 120 | 223 | 238 | 188 | 188 | 90 | 92 | 108 | 108 | 139 | 141 | 83 | 99 | 247 | 265 | 199 | 199 |
| Ar12S_6609 | 17 | 43.59 | 4.438 | 236 | 239 | 296 | 306 | 120 | 120 | 223 | 234 | 189 | 189 | 90 | 90 | 107 | 108 | 141 | 143 | 99 | 99 | 265 | 269 | 199 | 199 |
| Ar12S_6610 | 17 | 43.59 | 4.438 | 236 | 236 | 306 | 306 | 120 | 120 | 234 | 238 | 188 | 188 | 92 | 96 | 107 | 107 | 143 | 143 | 108 | 108 | 246 | 247 | 199 | 199 |
| Ar12S_6611 | 17 | 43.59 | 4.438 | 236 | 236 | 306 | 315 | 120 | 120 | -9 | -9 | 189 | 191 | -9 | -9 | 107 | 107 | 139 | 141 | 99 | 99 | 246 | 246 | 199 | 226 |
| Ar12S_6612 | 17 | 43.59 | 4.438 | 236 | 239 | 304 | 306 | 120 | 120 | 223 | 234 | 188 | 188 | -9 | -9 | 107 | 109 | 139 | 141 | 83 | 99 | 246 | 247 | 199 | 199 |
| Ar12S_6613 | 17 | 43.59 | 4.438 | 236 | 236 | 296 | 306 | 120 | 120 | 234 | 238 | 189 | 189 | 90 | 96 | 111 | 114 | 143 | 143 | 99 | 99 | -9 | -9 | 199 | 199 |
| Ar12S_6614 | 17 | 43.59 | 4.438 | 238 | 238 | 300 | 300 | 120 | 120 | 223 | 234 | 188 | 191 | 90 | 92 | 107 | 111 | 141 | 143 | 99 | 99 | 247 | 247 | 199 | 199 |
| Ar12S_6615 | 17 | 43.59 | 4.438 | 236 | 239 | 296 | 296 | 120 | 120 | 223 | 234 | 188 | 191 | -9 | -9 | 108 | 111 | 141 | 143 | 99 | 99 | 247 | 265 | 199 | 199 |
| Ar12S_6616 | 17 | 43.59 | 4.438 | 236 | 236 | 296 | 306 | 120 | 123 | 234 | 234 | 188 | 188 | 90 | 92 | 107 | 107 | 143 | 143 | 108 | 108 | 246 | 269 | 199 | 199 |
| Ar12S_6617 | 17 | 43.59 | 4.438 | 236 | 236 | 306 | 306 | 114 | 123 | 234 | 238 | 188 | 189 | 92 | 92 | 107 | 107 | 143 | 143 | 108 | 108 | 246 | 269 | 199 | 199 |
| Ar12S_6618 | 17 | 43.59 | 4.438 | 236 | 236 | 306 | 306 | 114 | 120 | 234 | 238 | 189 | 189 | 90 | 92 | 107 | 107 | 141 | 141 | 99 | 99 | 247 | 265 | 199 | 199 |
| Ar12S_6619 | 17 | 43.59 | 4.438 | 236 | 236 | 306 | 306 | 114 | 123 | 234 | 238 | 188 | 188 | 92 | 92 | 107 | 107 | 141 | 143 | 99 | 108 | 246 | 269 | 199 | 199 |
| Ar12S_6620 | 17 | 43.59 | 4.438 | 236 | 236 | 304 | 306 | 120 | 123 | 234 | 234 | 189 | 191 | 90 | 92 | 100 | 100 | 141 | 143 | -9 | -9 | 246 | 265 | 199 | 199 |
| Ar12S_6621 | 17 | 43.59 | 4.438 | 236 | 239 | 306 | 313 | 120 | 120 | 234 | 234 | 189 | 189 | 90 | 90 | 107 | 107 | 141 | 141 | 89 | 99 | 265 | 269 | 199 | 199 |
| Ar12S_6622 | 17 | 43.59 | 4.438 | 236 | 236 | 306 | 313 | 114 | 120 | 234 | 234 | 189 | 189 | 90 | 90 | 107 | 108 | 141 | 143 | 99 | 99 | 265 | 269 | 199 | 199 |
| Ar12S_6623 | 17 | 43.59 | 4.438 | 234 | 234 | 300 | 300 | 120 | 123 | 234 | 240 | 189 | 189 | 96 | 96 | 107 | 107 | 141 | 143 | 83 | 108 | 265 | 269 | 199 | 199 |
| Ar12S_6624 | 17 | 43.59 | 4.438 | 236 | 239 | 306 | 313 | 114 | 120 | 223 | 234 | 189 | 189 | 90 | 92 | -9 | -9 | 141 | 143 | 99 | 99 | 246 | 265 | 199 | 199 |
| Ar12S_6625 | 17 | 43.59 | 4.438 | 236 | 236 | 306 | 315 | 120 | 120 | 234 | 234 | 189 | 191 | 92 | 96 | 107 | 107 | 141 | 143 | 83 | 99 | 246 | 269 | 199 | 199 |
| Ar12S_6626 | 17 | 43.59 | 4.438 | 236 | 236 | 304 | 306 | 120 | 120 | 234 | 234 | 188 | 191 | 90 | 90 | 107 | 108 | 141 | 141 | 99 | 99 | 246 | 265 | 199 | 199 |
| Ar12S_6627 | 17 | 43.59 | 4.438 | 236 | 236 | 296 | 304 | 120 | 120 | 234 | 238 | 189 | 189 | 90 | 92 | 107 | 114 | 141 | 143 | 99 | 99 | 247 | 265 | 199 | 199 |
| Ar12S_6628 | 17 | 43.59 | 4.438 | 236 | 236 | 296 | 306 | 114 | 120 | 234 | 234 | 189 | 189 | 90 | 92 | 107 | 107 | 141 | 141 | 99 | 99 | 247 | 265 | 199 | 199 |
| Ar12T_6629 | 18 | 43.59 | 4.438 | 236 | 236 | 304 | 313 | 120 | 120 | 223 | 223 | 189 | 189 | 92 | 96 | 107 | 107 | 141 | 143 | 83 | 108 | 246 | 247 | 199 | 199 |
| Ar12T_6630 | 18 | 43.59 | 4.438 | 236 | 236 | 291 | 306 | 120 | 120 | 234 | 234 | 189 | 189 | 92 | 96 | 107 | 107 | 139 | 143 | 99 | 99 | 246 | 247 | 199 | 199 |
| Ar12T_6631 | 18 | 43.59 | 4.438 | 236 | 236 | 296 | 296 | 120 | 120 | -9 | -9 | 189 | 191 | 92 | 96 | 107 | 107 | 139 | 141 | 83 | 99 | 246 | 247 | 199 | 199 |
| Ar12T_6632 | 18 | 43.59 | 4.438 | 236 | 236 | 306 | 313 | 120 | 120 | 223 | 223 | 189 | 191 | 90 | 90 | 107 | 111 | 139 | 143 | 99 | 99 | 246 | 246 | 199 | 199 |
| Ar12T_6633 | 18 | 43.59 | 4.438 | 236 | 236 | 306 | 313 | 120 | 120 | 223 | 238 | 189 | 191 | 96 | 96 | 107 | 111 | 139 | 143 | 99 | 99 | 246 | 246 | 199 | 199 |
| Ar12T_6634 | 18 | 43.59 | 4.438 | 236 | 236 | 304 | 304 | 120 | 120 | 234 | 234 | 191 | 191 | 90 | 90 | 107 | 107 | 141 | 141 | 83 | 89 | 246 | 265 | 199 | 199 |
| Ar12T_6635 | 18 | 43.59 | 4.438 | 228 | 239 | 306 | 306 | 120 | 120 | 223 | 234 | 189 | 189 | 90 | 90 | -9 | -9 | 141 | 143 | 99 | 108 | 246 | 246 | 199 | 199 |
| Ar12T_6636 | 18 | 43.59 | 4.438 | 236 | 236 | 291 | 313 | 120 | 120 | 223 | 234 | 189 | 189 | 92 | 92 | 107 | 107 | 141 | 143 | 83 | 99 | 246 | 265 | 199 | 199 |
| Ar12T_6637 | 18 | 43.59 | 4.438 | 236 | 236 | 306 | 313 | 120 | 120 | 223 | 238 | 189 | 189 | 92 | 96 | 107 | 107 | 143 | 143 | 89 | 99 | 246 | 246 | 199 | 226 |
| Ar12T_6638 | 18 | 43.59 | 4.438 | 236 | 236 | 306 | 306 | 114 | 120 | 223 | 223 | 193 | 193 | 90 | 94 | 107 | 107 | 143 | 143 | 99 | 99 | 246 | 246 | 199 | 199 |
| Ar12T_6639 | 18 | 43.59 | 4.438 | 236 | 236 | 306 | 306 | 120 | 123 | 234 | 234 | 189 | 189 | 90 | 92 | 107 | 107 | 141 | 141 | 99 | 99 | 265 | 269 | 199 | 199 |
| Ar12T_6640 | 18 | 43.59 | 4.438 | 236 | 236 | 304 | 306 | 120 | 123 | 234 | 240 | 188 | 189 | 92 | 92 | 107 | 107 | 141 | 141 | 83 | 99 | -9 | -9 | 199 | 199 |
| Ar12T_6641 | 18 | 43.59 | 4.438 | 236 | 236 | 306 | 313 | 120 | 120 | 223 | 223 | 189 | 189 | 90 | 90 | -9 | -9 | 143 | 143 | 89 | 99 | 246 | 269 | 199 | 199 |
| Ar12T_6642 | 18 | 43.59 | 4.438 | 236 | 236 | 304 | 304 | 120 | 120 | 223 | 234 | 189 | 191 | 90 | 96 | 107 | 107 | 141 | 143 | 83 | 108 | 247 | 265 | 199 | 199 |
| Ar12T_6643 | 18 | 43.59 | 4.438 | 236 | 236 | 296 | 306 | 120 | 120 | 223 | 234 | 189 | 191 | 92 | 96 | 107 | 109 | 143 | 143 | 89 | 99 | 246 | 246 | 199 | 226 |
| Ar12T_6644 | 18 | 43.59 | 4.438 | 236 | 236 | 296 | 304 | 120 | 120 | 223 | 238 | 191 | 191 | 90 | 92 | 107 | 111 | 141 | 143 | 99 | 99 | 247 | 247 | 199 | 226 |
| Ar12T_6645 | 18 | 43.59 | 4.438 | 236 | 236 | 291 | 304 | 120 | 120 | 234 | 234 | 189 | 189 | 90 | 96 | 107 | 107 | 141 | 143 | 83 | 99 | 246 | 265 | 199 | 199 |
| Ar12T_6646 | 18 | 43.59 | 4.438 | 239 | 239 | 306 | 306 | 120 | 123 | 223 | 234 | 188 | 189 | 90 | 90 | -9 | -9 | 141 | 143 | 99 | 108 | 246 | 246 | 199 | 199 |
| Ar12T_6647 | 18 | 43.59 | 4.438 | 236 | 239 | 296 | 306 | 120 | 120 | 234 | 240 | 188 | 191 | 90 | 90 | -9 | -9 | 141 | 143 | 99 | 99 | 247 | 265 | 199 | 199 |
| Ar12T_6648 | 18 | 43.59 | 4.438 | 236 | 236 | 296 | 306 | 120 | 120 | 234 | 234 | 189 | 189 | 90 | 92 | 107 | 107 | 143 | 143 | 83 | 99 | 246 | 247 | 199 | 199 |
| Ar12T_6649 | 18 | 43.59 | 4.438 | 236 | 239 | 296 | 306 | 120 | 120 | 234 | 240 | 189 | 189 | 90 | 92 | 108 | 111 | 143 | 176 | 99 | 108 | 265 | 269 | 199 | 199 |
| Ar12T_6650 | 18 | 43.59 | 4.438 | 236 | 236 | 306 | 306 | 123 | 123 | 223 | 234 | 188 | 191 | 90 | 96 | 107 | 107 | 139 | 143 | 99 | 99 | 246 | 246 | 199 | 199 |
| Ar12T_6651 | 18 | 43.59 | 4.438 | 236 | 236 | 291 | 313 | 120 | 120 | 223 | 234 | 189 | 191 | 90 | 92 | 107 | 107 | 141 | 143 | 83 | 108 | 246 | 247 | 199 | 199 |
| Ar12T_6652 | 18 | 43.59 | 4.438 | 236 | 236 | 296 | 304 | 120 | 120 | 223 | 238 | 188 | 191 | 90 | 90 | 109 | 111 | 141 | 143 | 99 | 99 | 247 | 269 | 199 | 226 |
| Ar12T_6653 | 18 | 43.59 | 4.438 | 236 | 236 | 296 | 296 | 120 | 120 | 223 | 223 | 189 | 189 | 90 | 90 | 107 | 107 | 141 | 143 | 99 | 99 | 246 | 269 | 199 | 199 |
| Ar12T_6654 | 18 | 43.59 | 4.438 | 236 | 236 | 291 | 304 | 120 | 120 | 234 | 234 | 189 | 191 | 90 | 90 | 107 | 114 | 141 | 143 | 83 | 83 | 247 | 265 | 199 | 199 |
| Ar12T_6655 | 18 | 43.59 | 4.438 | 236 | 236 | 304 | 313 | 120 | 120 | 223 | 223 | 189 | 189 | 90 | 96 | 107 | 107 | 141 | 143 | 83 | 108 | 246 | 265 | 199 | 199 |
| Ar12T_6656 | 18 | 43.59 | 4.438 | 221 | 236 | 296 | 304 | 120 | 120 | 234 | 234 | 189 | 191 | 92 | 96 | 107 | 107 | 139 | 141 | 89 | 108 | 246 | 246 | 199 | 199 |
| Ar12T_6657 | 18 | 43.59 | 4.438 | 236 | 236 | 296 | 304 | 120 | 120 | 234 | 238 | 189 | 189 | 90 | 90 | 107 | 107 | 141 | 143 | 99 | 99 | 246 | 269 | 199 | 199 |
| Ar12T_6658 | 18 | 43.59 | 4.438 | 236 | 236 | 291 | 313 | 123 | 123 | 234 | 234 | 188 | 189 | 90 | 96 | 107 | 109 | 141 | 141 | 83 | 83 | 246 | 265 | 199 | 226 |
| Ar12T_6659 | 18 | 43.59 | 4.438 | 236 | 236 | 304 | 313 | 120 | 120 | 223 | 223 | 189 | 191 | 90 | 92 | 100 | 100 | 141 | 143 | -9 | -9 | 246 | 246 | 199 | 199 |
| Ar12T_6660 | 18 | 43.59 | 4.438 | 236 | 239 | 306 | 313 | 120 | 120 | 223 | 240 | 188 | 189 | 92 | 92 | 107 | 114 | 141 | 141 | 83 | 108 | 246 | 246 | 199 | 199 |
| Ar12T_6661 | 18 | 43.59 | 4.438 | 236 | 236 | 304 | 306 | 120 | 120 | 238 | 240 | 188 | 189 | 90 | 92 | 107 | 111 | 143 | 176 | 99 | 108 | 265 | 269 | 199 | 199 |
| Ar12T_6662 | 18 | 43.59 | 4.438 | 236 | 239 | 296 | 306 | 120 | 120 | 223 | 234 | 189 | 191 | 92 | 96 | 107 | 111 | 139 | 143 | 99 | 99 | 246 | 246 | 199 | 199 |
| Ar12T_6663 | 18 | 43.59 | 4.438 | 239 | 239 | 304 | 306 | 120 | 123 | 223 | 234 | 189 | 191 | 90 | 90 | 107 | 108 | 141 | 141 | 99 | 99 | 246 | 265 | 199 | 199 |
| Ar12T_6664 | 18 | 43.59 | 4.438 | 236 | 236 | 304 | 308 | 120 | 123 | 234 | 234 | 188 | 191 | 90 | 90 | 107 | 108 | 141 | 141 | 99 | 99 | 246 | 246 | 199 | 199 |
| Ar12T_6665 | 18 | 43.59 | 4.438 | 236 | 236 | 306 | 306 | 120 | 123 | 223 | 234 | 189 | 189 | 92 | 96 | 107 | 107 | 141 | 141 | 89 | 99 | 246 | 246 | 199 | 199 |
| Ar12T_6666 | 18 | 43.59 | 4.438 | 236 | 236 | 304 | 306 | 120 | 120 | 234 | 234 | 189 | 191 | 90 | 90 | 108 | 108 | 141 | 141 | 99 | 99 | 265 | 269 | 199 | 199 |
| Ar12T_6667 | 18 | 43.59 | 4.438 | 236 | 236 | 296 | 313 | 114 | 120 | 223 | 234 | 189 | 189 | 92 | 92 | 107 | 107 | 143 | 143 | 83 | 99 | 246 | 247 | 199 | 199 |
| Ar12T_6668 | 18 | 43.59 | 4.438 | 236 | 236 | 304 | 306 | 120 | 123 | 234 | 234 | 189 | 189 | 90 | 90 | 108 | 108 | 141 | 143 | 99 | 108 | 265 | 269 | 199 | 199 |
| Ar13S_6725 | 19 | 43.62977 | 4.42562 | 236 | 236 | 303 | 304 | 120 | 120 | 223 | 223 | 188 | 188 | 90 | 96 | 107 | 108 | 141 | 143 | 108 | 108 | 246 | 246 | 199 | 199 |
| Ar13S_6726 | 19 | 43.62977 | 4.42562 | 236 | 236 | 296 | 306 | 114 | 120 | 234 | 234 | 188 | 189 | 90 | 96 | 107 | 111 | 141 | 143 | 83 | 83 | 269 | 269 | 199 | 199 |
| Ar14T_6721 | 20 | 43.684 | 4.513 | 236 | 236 | 296 | 306 | 120 | 120 | 234 | 238 | 189 | 191 | 90 | 90 | 107 | 108 | 141 | 141 | 99 | 99 | 246 | 246 | 199 | 226 |
| Ar14T_6722 | 20 | 43.684 | 4.513 | 236 | 236 | 291 | 306 | 120 | 120 | 223 | 234 | 189 | 191 | 90 | 90 | 107 | 108 | 141 | 141 | 99 | 99 | 246 | 246 | 226 | 226 |
| Ar14T_6723 | 20 | 43.684 | 4.513 | 236 | 236 | 303 | 306 | 123 | 123 | 238 | 238 | 188 | 189 | -9 | -9 | 111 | 114 | 141 | 143 | 99 | 108 | 246 | 246 | 199 | 199 |
| Ar14T_6724 | 20 | 43.684 | 4.513 | 236 | 236 | 296 | 313 | 120 | 120 | 234 | 238 | 189 | 189 | 90 | 90 | 105 | 107 | 143 | 143 | 99 | 99 | 247 | 265 | 199 | 199 |
| Ar15S_6727 | 21 | 43.541 | 4.355 | 236 | 239 | 296 | 304 | 120 | 120 | 223 | 238 | 188 | 189 | 90 | 92 | 107 | 107 | 139 | 143 | 83 | 83 | 246 | 246 | 199 | 199 |
| Ar15S_6728 | 21 | 43.541 | 4.355 | 236 | 236 | 303 | 304 | 123 | 123 | 234 | 234 | 188 | 191 | 90 | 92 | 107 | 107 | 143 | 143 | 83 | 89 | 269 | 269 | 199 | 226 |
| Ar15S_6729 | 21 | 43.541 | 4.355 | 236 | 236 | 303 | 304 | 120 | 123 | 234 | 234 | 188 | 191 | 90 | 90 | 107 | 111 | 143 | 143 | 83 | 89 | 269 | 269 | 199 | 199 |
| Ar15S_6730 | 21 | 43.541 | 4.355 | 236 | 239 | 304 | 306 | 120 | 120 | 234 | 238 | 189 | 191 | 92 | 92 | 107 | 107 | 139 | 143 | 83 | 99 | 246 | 269 | 199 | 199 |
| Ar15S_6731 | 21 | 43.541 | 4.355 | 236 | 236 | 306 | 306 | 123 | 123 | 234 | 234 | 191 | 191 | 90 | 92 | 107 | 107 | 141 | 143 | 83 | 89 | 246 | 246 | 199 | 199 |
| Ar15S_6732 | 21 | 43.541 | 4.355 | 236 | 236 | 306 | 306 | 120 | 123 | 234 | 234 | 188 | 188 | 90 | 90 | 107 | 111 | 143 | 143 | 83 | 89 | 246 | 246 | 226 | 226 |
| Ar15S_6733 | 21 | 43.541 | 4.355 | 236 | 239 | 296 | 304 | 120 | 123 | 223 | 238 | 188 | 191 | 92 | 92 | 107 | 108 | 139 | 139 | 83 | 99 | 246 | 246 | 199 | 199 |
| Ar15S_6734 | 21 | 43.541 | 4.355 | 236 | 236 | 303 | 304 | 123 | 123 | 234 | 234 | 188 | 191 | 90 | 92 | 107 | 111 | 141 | 143 | 83 | 89 | 246 | 246 | 199 | 199 |
| Ar15S_6735 | 21 | 43.541 | 4.355 | 236 | 239 | 304 | 304 | 120 | 120 | 234 | 238 | 188 | 189 | 92 | 92 | 107 | 107 | 139 | 143 | 83 | 83 | 246 | 246 | 199 | 199 |
| Ar15S_6736 | 21 | 43.541 | 4.355 | 236 | 236 | 303 | 306 | 120 | 123 | 234 | 234 | 191 | 191 | 90 | 92 | 107 | 107 | 141 | 143 | 83 | 89 | 246 | 269 | 199 | 199 |
| Ar15S_6984 | 21 | 43.541 | 4.355 | 236 | 236 | 303 | 304 | 120 | 123 | 234 | 234 | 188 | 188 | 90 | 92 | 107 | 111 | 143 | 143 | 83 | 89 | 246 | 269 | 199 | 199 |
| Ar15S_6985 | 21 | 43.541 | 4.355 | 236 | 236 | 296 | 304 | 114 | 120 | 234 | 238 | 189 | 191 | 92 | 92 | 107 | 107 | 143 | 143 | 99 | 108 | 265 | 269 | 199 | 199 |
| Ar15S_6986 | 21 | 43.541 | 4.355 | 236 | 236 | 303 | 306 | 114 | 114 | 234 | 240 | 189 | 189 | 94 | 96 | 107 | 108 | 139 | 143 | 83 | 99 | 246 | 246 | 199 | 199 |
| Ar15S_6987 | 21 | 43.541 | 4.355 | 236 | 236 | 306 | 313 | 120 | 120 | 234 | 240 | 189 | 189 | 90 | 92 | 107 | 107 | 143 | 143 | 99 | 99 | 246 | 265 | 199 | 199 |
| Ar15S_6988 | 21 | 43.541 | 4.355 | 236 | 236 | 296 | 306 | 120 | 120 | 234 | 238 | 189 | 189 | 90 | 92 | 107 | 107 | 143 | 143 | 99 | 99 | 265 | 265 | 199 | 199 |
| Ar15S_6989 | 21 | 43.541 | 4.355 | 236 | 239 | 304 | 306 | 120 | 120 | 234 | 238 | 189 | 191 | 92 | 92 | 107 | 107 | 139 | 143 | 83 | 99 | 246 | 246 | 199 | 199 |
| Ar15S_6990 | 21 | 43.541 | 4.355 | 236 | 236 | 306 | 306 | 120 | 120 | 234 | 238 | 189 | 191 | 90 | 92 | 107 | 107 | 143 | 143 | 99 | 108 | 265 | 265 | 199 | 199 |
| Ar15S_6991 | 21 | 43.541 | 4.355 | 236 | 236 | 291 | 306 | 120 | 120 | 234 | 234 | 188 | 189 | 90 | 90 | 107 | 107 | 141 | 143 | 83 | 108 | 246 | 246 | 199 | 199 |
| Ar15S_6992 | 21 | 43.541 | 4.355 | 236 | 239 | 296 | 313 | 120 | 123 | 234 | 238 | 189 | 191 | 92 | 92 | 107 | 108 | 143 | 143 | 99 | 99 | 247 | 265 | 199 | 199 |
| Ar15S_6993 | 21 | 43.541 | 4.355 | 236 | 236 | 306 | 306 | 120 | 120 | 238 | 238 | 188 | 189 | 90 | 96 | 107 | 107 | 141 | 141 | 99 | 99 | 246 | 247 | 199 | 199 |
| Ar15S_6994 | 21 | 43.541 | 4.355 | 236 | 236 | 296 | 306 | 114 | 120 | 234 | 238 | 189 | 191 | 96 | 96 | 107 | 108 | 139 | 143 | 87 | 87 | 246 | 265 | 199 | 226 |
| Ar15S_6995 | 21 | 43.541 | 4.355 | 236 | 236 | 291 | 306 | 120 | 120 | 234 | 234 | 188 | 189 | 90 | 90 | 107 | 107 | 141 | 143 | 99 | 108 | 246 | 266 | 199 | 199 |
| Ar15S_6996 | 21 | 43.541 | 4.355 | 236 | 236 | 304 | 306 | 120 | 120 | 234 | 240 | 188 | 189 | 90 | 90 | 107 | 107 | 139 | 143 | 99 | 99 | 265 | 265 | 199 | 199 |
| Ar15S_6998 | 21 | 43.541 | 4.355 | 236 | 236 | 306 | 306 | 114 | 120 | 234 | 238 | 189 | 191 | 90 | 92 | 107 | 107 | 143 | 143 | 99 | 108 | 265 | 269 | 199 | 199 |
| Ar15S_6999 | 21 | 43.541 | 4.355 | 236 | 236 | 306 | 306 | 114 | 120 | 234 | 238 | 188 | 191 | 90 | 92 | 107 | 107 | 143 | 143 | 108 | 108 | 265 | 269 | 199 | 199 |
| Ar15S_7000 | 21 | 43.541 | 4.355 | 236 | 236 | 296 | 306 | 114 | 120 | 234 | 238 | 189 | 189 | 92 | 92 | 107 | 107 | 143 | 143 | 99 | 99 | 265 | 269 | 199 | 199 |
| Ar15S_7001 | 21 | 43.541 | 4.355 | 236 | 236 | 304 | 306 | 120 | 120 | 234 | 240 | 189 | 191 | 96 | 96 | 107 | 108 | 141 | 143 | 99 | 99 | 247 | 266 | 199 | 199 |
| Ar15S_7002 | 21 | 43.541 | 4.355 | 236 | 236 | 296 | 313 | 120 | 123 | 234 | 238 | 189 | 191 | 90 | 90 | 107 | 108 | 143 | 143 | 99 | 99 | 247 | 269 | 199 | 199 |
| Ar15S_7003 | 21 | 43.541 | 4.355 | 236 | 236 | 296 | 306 | 120 | 120 | 223 | 238 | 188 | 189 | 94 | 94 | 107 | 107 | 139 | 143 | 99 | 99 | 246 | 246 | 199 | 199 |
| Ar15S_7004 | 21 | 43.541 | 4.355 | 236 | 236 | 306 | 306 | 120 | 120 | 234 | 238 | 189 | 189 | 90 | 92 | 107 | 107 | 143 | 143 | 99 | 99 | 265 | 265 | 199 | 199 |
| Ar15S_7005 | 21 | 43.541 | 4.355 | 236 | 236 | 291 | 296 | 120 | 120 | 234 | 234 | 189 | 189 | 90 | 90 | 107 | 107 | 141 | 143 | 83 | 108 | 246 | 246 | 199 | 199 |
| Ar16T_6747 | 22 | 43.605 | 4.486 | 236 | 236 | 304 | 306 | 120 | 120 | 234 | 238 | 189 | 191 | 90 | 90 | 107 | 114 | 141 | 141 | 83 | 99 | 247 | 247 | 199 | 199 |
| Ar16S_6737 | 23 | 43.605 | 4.486 | 236 | 239 | 304 | 306 | 114 | 120 | 234 | 240 | 189 | 191 | 90 | 96 | 107 | 114 | 143 | 143 | 99 | 99 | 246 | 246 | 199 | 199 |
| Ar16S_6738 | 23 | 43.605 | 4.486 | 228 | 228 | 296 | 306 | 120 | 120 | 234 | 238 | 189 | 191 | 92 | 92 | 107 | 108 | 141 | 141 | 99 | 99 | 269 | 269 | 199 | 199 |
| Ar16S_6739 | 23 | 43.605 | 4.486 | 236 | 236 | 296 | 306 | 120 | 123 | 234 | 238 | 189 | 189 | 92 | 92 | 107 | 108 | 139 | 176 | 83 | 99 | 247 | 247 | 199 | 199 |
| Ar16S_6740 | 23 | 43.605 | 4.486 | 236 | 236 | 306 | 306 | 120 | 120 | 234 | 234 | 189 | 191 | 92 | 92 | 107 | 108 | 141 | 143 | 99 | 99 | 246 | 246 | 199 | 199 |
| Ar16S_6741 | 23 | 43.605 | 4.486 | 236 | 236 | 296 | 313 | 114 | 120 | 234 | 234 | 189 | 191 | 90 | 96 | 107 | 114 | 141 | 143 | 99 | 108 | 246 | 247 | 199 | 199 |
| Ar16S_6742 | 23 | 43.605 | 4.486 | 236 | 236 | 296 | 306 | 114 | 123 | 234 | 238 | 189 | 189 | 92 | 92 | 107 | 107 | 141 | 176 | 99 | 99 | 247 | 269 | 199 | 199 |
| Ar16S_6743 | 23 | 43.605 | 4.486 | 236 | 236 | 296 | 304 | 120 | 120 | 234 | 234 | 189 | 189 | 92 | 96 | 108 | 108 | 139 | 141 | 99 | 99 | 246 | 246 | 199 | 199 |
| Ar16S_6744 | 23 | 43.605 | 4.486 | 236 | 236 | 306 | 306 | 114 | 120 | 223 | 234 | 188 | 188 | 90 | 96 | 107 | 107 | 139 | 141 | 83 | 99 | 247 | 269 | 199 | 199 |
| Ar16S_6745 | 23 | 43.605 | 4.486 | 236 | 236 | 306 | 313 | 114 | 120 | 234 | 234 | 189 | 191 | 90 | 96 | 107 | 107 | 143 | 143 | 99 | 108 | 246 | 247 | 199 | 199 |
| Ar16S_6746 | 23 | 43.605 | 4.486 | 236 | 236 | 296 | 306 | 114 | 120 | 234 | 234 | 188 | 189 | 96 | 96 | 107 | 107 | 139 | 139 | 83 | 99 | 247 | 269 | 199 | 199 |
| Ar16S_7006 | 23 | 43.605 | 4.486 | 236 | 239 | 296 | 304 | 114 | 120 | 234 | 234 | 189 | 189 | 90 | 90 | 107 | 108 | 141 | 143 | 99 | 99 | 247 | 265 | 199 | 226 |
| Ar16S_7007 | 23 | 43.605 | 4.486 | 236 | 236 | 296 | 306 | 120 | 123 | 234 | 238 | 189 | 189 | 92 | 92 | 107 | 108 | 141 | 176 | 99 | 99 | 247 | 269 | 199 | 199 |
| Ar16S_7008 | 23 | 43.605 | 4.486 | 236 | 239 | 306 | 306 | 120 | 120 | 234 | 234 | 191 | 191 | 90 | 92 | 107 | 111 | 143 | 143 | 83 | 99 | 246 | 269 | 199 | 199 |
| Ar16S_7009 | 23 | 43.605 | 4.486 | 236 | 236 | 296 | 306 | 114 | 120 | 234 | 234 | 189 | 191 | 90 | 90 | 107 | 108 | 141 | 143 | 83 | 99 | 246 | 265 | 199 | 199 |
| Ar16S_7010 | 23 | 43.605 | 4.486 | 236 | 236 | 296 | 306 | 120 | 120 | 234 | 234 | 189 | 189 | 92 | 92 | 107 | 108 | 139 | 141 | 99 | 99 | 247 | 247 | 199 | 199 |
| Ar16S_7011 | 23 | 43.605 | 4.486 | 236 | 236 | 296 | 306 | 120 | 120 | 234 | 234 | 189 | 189 | 92 | 92 | 108 | 108 | 139 | 143 | 99 | 99 | 246 | 247 | 199 | 199 |
| Ar16S_7012 | 23 | 43.605 | 4.486 | 236 | 239 | 306 | 306 | 114 | 120 | 234 | 234 | 188 | 189 | 90 | 90 | 107 | 111 | 139 | 139 | 89 | 99 | 246 | 247 | 199 | 199 |
| Ar16S_7013 | 23 | 43.605 | 4.486 | 236 | 236 | 291 | 306 | 120 | 120 | 234 | 234 | 189 | 189 | 90 | 92 | 107 | 107 | 141 | 141 | 99 | 108 | 246 | 265 | 226 | 226 |
| Ar16S_7014 | 23 | 43.605 | 4.486 | 236 | 236 | 306 | 306 | 120 | 120 | 234 | 240 | 188 | 189 | 90 | 90 | 107 | 114 | 139 | 143 | 99 | 99 | 246 | 247 | 199 | 199 |
| Ar16S_7015 | 23 | 43.605 | 4.486 | 236 | 236 | 296 | 306 | 114 | 120 | 234 | 234 | 188 | 189 | 90 | 90 | 107 | 107 | 139 | 141 | 99 | 99 | 247 | 269 | 199 | 199 |
| Ar16S_7016 | 23 | 43.605 | 4.486 | 236 | 236 | 291 | 304 | 120 | 120 | 223 | 234 | 189 | 189 | 96 | 96 | 108 | 108 | 139 | 143 | 99 | 99 | 246 | 246 | 199 | 199 |
| Ar16S_7017 | 23 | 43.605 | 4.486 | 236 | 236 | 296 | 314 | 120 | 120 | 223 | 234 | 188 | 191 | 90 | 92 | 107 | 107 | 143 | 176 | 99 | 99 | 246 | 269 | 199 | 199 |
| Ar16S_7018 | 23 | 43.605 | 4.486 | 236 | 239 | 296 | 306 | 114 | 120 | 234 | 234 | 189 | 191 | 90 | 92 | 107 | 108 | 141 | 143 | 83 | 99 | 246 | 246 | 199 | 226 |
| Ar16S_7019 | 23 | 43.605 | 4.486 | 236 | 236 | 296 | 304 | 120 | 120 | 234 | 234 | 189 | 191 | 90 | 90 | 107 | 108 | 143 | 143 | 89 | 108 | 246 | 265 | 199 | 199 |
| Ar16S_7020 | 23 | 43.605 | 4.486 | 236 | 239 | 296 | 304 | 114 | 120 | 234 | 234 | 188 | 189 | 90 | 96 | 107 | 108 | 141 | 143 | 99 | 99 | 246 | 269 | 199 | 199 |
| Ar16S_7021 | 23 | 43.605 | 4.486 | 236 | 236 | 303 | 313 | 114 | 120 | 234 | 234 | 188 | 191 | 90 | 96 | 107 | 107 | 143 | 143 | 99 | 99 | 246 | 269 | 199 | 199 |
| Ar17T_6669 | 24 | 43.544 | 4.686 | 236 | 236 | 291 | 296 | 120 | 120 | 234 | 234 | 188 | 188 | 90 | 92 | 108 | 108 | 143 | 143 | 99 | 99 | 246 | 246 | 199 | 199 |
| Ar17T_6670 | 24 | 43.544 | 4.686 | 236 | 236 | 291 | 306 | 114 | 120 | 234 | 240 | 189 | 189 | 90 | 92 | 107 | 107 | 143 | 143 | 89 | 99 | 246 | 247 | 199 | 199 |
| Ar17T_6671 | 24 | 43.544 | 4.686 | 236 | 236 | 304 | 313 | 120 | 120 | 234 | 240 | 191 | 191 | 90 | 90 | 107 | 114 | 141 | 141 | 99 | 99 | 246 | 269 | 199 | 199 |
| Ar17T_6672 | 24 | 43.544 | 4.686 | 236 | 236 | 306 | 313 | 120 | 120 | 234 | 238 | 189 | 189 | 90 | 96 | 107 | 108 | 139 | 141 | 99 | 99 | 246 | 247 | 199 | 199 |
| Ar17T_6673 | 24 | 43.544 | 4.686 | 236 | 236 | 296 | 304 | 120 | 123 | 234 | 234 | 189 | 189 | 92 | 96 | 107 | 108 | 141 | 141 | 83 | 99 | 246 | 265 | 199 | 199 |
| Ar17T_6674 | 24 | 43.544 | 4.686 | 236 | 236 | 304 | 304 | 120 | 120 | -9 | -9 | 189 | 191 | 90 | 92 | 107 | 107 | 139 | 143 | 99 | 99 | 246 | 247 | 199 | 199 |
| Ar17T_6675 | 24 | 43.544 | 4.686 | 236 | 236 | 304 | 313 | 120 | 120 | 234 | 234 | 191 | 191 | 90 | 90 | 107 | 107 | 141 | 141 | 99 | 99 | 246 | 269 | 199 | 199 |
| Ar18T_6748 | 25 | 43.727 | 4.658 | 236 | 236 | 296 | 304 | 120 | 123 | 234 | 234 | 188 | 188 | 90 | 90 | 108 | 111 | 141 | 141 | 83 | 99 | 246 | 247 | 199 | 199 |
| Ar18T_6749 | 25 | 43.727 | 4.658 | 236 | 236 | 296 | 306 | 120 | 123 | 234 | 240 | 191 | 191 | 90 | 90 | 107 | 108 | 139 | 141 | 83 | 99 | 246 | 246 | 199 | 199 |
| Ar18T_6750 | 25 | 43.727 | 4.658 | 236 | 236 | 296 | 304 | 120 | 120 | 234 | 234 | 189 | 189 | 90 | 90 | 107 | 108 | 141 | 143 | 99 | 108 | 246 | 246 | 199 | 226 |
| Ar18T_6751 | 25 | 43.727 | 4.658 | 236 | 236 | 296 | 304 | 120 | 120 | 234 | 234 | 189 | 191 | 90 | 92 | 107 | 111 | 143 | 143 | 89 | 89 | 246 | 247 | 199 | 199 |
| Ar18T_6752 | 25 | 43.727 | 4.658 | 236 | 236 | 296 | 306 | 120 | 120 | 223 | 234 | 189 | 191 | 90 | 92 | 107 | 108 | 139 | 141 | 83 | 99 | 246 | 246 | 199 | 199 |
| Ar18T_6753 | 25 | 43.727 | 4.658 | 236 | 236 | 291 | 296 | 120 | 120 | 234 | 240 | 188 | 188 | 90 | 92 | 108 | 111 | 141 | 141 | 83 | 99 | 246 | 247 | 199 | 199 |
| Ar18T_6754 | 25 | 43.727 | 4.658 | 236 | 236 | 291 | 296 | 120 | 120 | 234 | 234 | 189 | 191 | 90 | 90 | 107 | 111 | 143 | 143 | 89 | 89 | 246 | 247 | 199 | 199 |
| Ar18T_6755 | 25 | 43.727 | 4.658 | 236 | 236 | 306 | 306 | 120 | 123 | 234 | 240 | 191 | 191 | 90 | 92 | 107 | 107 | 139 | 141 | 83 | 99 | 265 | 265 | 199 | 199 |
| Ar18T_6757 | 25 | 43.727 | 4.658 | 236 | 236 | 291 | 296 | -9 | -9 | 234 | 240 | 188 | 189 | 90 | 92 | 107 | 108 | 141 | 143 | 89 | 99 | 246 | 247 | 199 | 199 |

S1 Table : microsatelite Data

Ind : individual ID, Pop: Putative population of individuals, from Column 4, Microsatellite Markers Used
