# Supplementary material for: Population genetics of the Mediterranean corn borer (Sesamia nonagrioides) differs between wild and cultivated plants
Source: PLoS One. 2020 Mar 19;15(3):e0230434. doi: 10.1371/journal.pone.0230434 (PMC7081988; doi:10.1371/journal.pone.0230434)
Supplement: S2 Table — Pop: Putative population, Loc: microsatellite loci. (DOCX) [file pone.0230434.s008.docx]

| Pop | Locality | Loc_1 | Loc_2 | Loc_3 | Loc_4 | Loc_5 | Loc_6 | Loc_7 | Loc_8 | Loc_9 | Loc_10 | Loc_11 |
| --- | --- | --- | --- | --- | --- | --- | --- | --- | --- | --- | --- | --- |
| 1 | Longage 1 (31) | 2 | 7 | 3 | 4 | 3 | 3 | 4 | 4 | 3 | 4 | 1 |
| 2 | Longage 2 (31) | 3 | 5 | 3 | 5 | 3 | 4 | 4 | 4 | 3 | 4 | 1 |
| 3 | St-Clar-de-Rivière (31) | 2 | 5 | 3 | 3 | 2 | 4 | 4 | 3 | 3 | 4 | 1 |
| 4 | Longages 3 (31) | 2 | 6 | 3 | 3 | 3 | 4 | 4 | 3 | 3 | 4 | 1 |
| 5 | Longages 4 (31) | 2 | 6 | 2 | 4 | 3 | 4 | 4 | 3 | 3 | 4 | 1 |
| 6 | Poucharramet (31) | 2 | 7 | 2 | 4 | 3 | 3 | 4 | 3 | 3 | 4 | 1 |
| 7 | Cambernard (31) | 2 | 7 | 2 | 4 | 3 | 3 | 4 | 5 | 3 | 4 | 1 |
| 8 | Lavergne (46) | 1 | 3 | 2 | 3 | 2 | 2 | 2 | 3 | 2 | 2 | 1 |
| 9 | Aubenas (07) | 1 | 3 | 3 | 4 | 2 | 2 | 2 | 1 | 1 | 2 | 1 |
| 10 | Arles 2 (13) | 1 | 2 | 3 | 3 | 3 | 1 | 2 | 2 | 2 | 2 | 1 |
| 11 | Arles 3 (13) | 3 | 5 | 3 | 4 | 3 | 3 | 4 | 3 | 4 | 4 | 1 |
| 12 | Arles 4 (13) | 1 | 3 | 3 | 3 | 2 | 3 | 4 | 3 | 1 | 3 | 2 |
| 13 | Arles 6 (13) | 1 | 2 | 1 | 1 | 1 | 2 | 2 | 1 | 2 | 1 | 1 |
| 14 | Arles 7 (13) | 3 | 6 | 3 | 4 | 3 | 3 | 4 | 4 | 4 | 4 | 1 |
| 15 | Arles 8 (13) | 2 | 6 | 3 | 3 | 3 | 4 | 1 | 3 | 2 | 3 | 1 |
| 16 | Arles 9 (13) | 1 | 2 | 1 | 2 | 1 | 2 | 1 | 2 | 1 | 1 | 1 |
| 17 | Arles 12 (13) | 4 | 7 | 3 | 4 | 3 | 3 | 7 | 3 | 5 | 5 | 2 |
| 18 | Arles 12 (13) | 4 | 6 | 3 | 4 | 4 | 4 | 6 | 4 | 4 | 4 | 2 |
| 19 | Arles 13 (13) | 1 | 4 | 2 | 2 | 2 | 2 | 3 | 2 | 2 | 2 | 1 |
| 20 | Arles 14 (13) | 1 | 5 | 2 | 3 | 3 | 1 | 5 | 2 | 2 | 3 | 2 |
| 21 | Arles 15 (13) | 2 | 6 | 3 | 4 | 3 | 4 | 3 | 3 | 5 | 5 | 2 |
| 22 | Arles 16 (13) | 1 | 2 | 1 | 2 | 2 | 1 | 2 | 1 | 2 | 1 | 1 |
| 23 | Arles 16 (13) | 3 | 7 | 3 | 4 | 3 | 3 | 4 | 4 | 4 | 4 | 2 |
| 24 | Arles 17 (13) | 1 | 5 | 3 | 3 | 3 | 3 | 3 | 3 | 3 | 4 | 1 |
| 25 | Arles 18 (13) | 1 | 4 | 2 | 3 | 3 | 2 | 3 | 3 | 4 | 3 | 2 |
| ALL |  | 6 | 13 | 3 | 5 | 4 | 5 | 8 | 6 | 6 | 6 | 2 |

S2 table : number of alleles per locus (loc) and per population. Pop : Putative population, Loc: microsatellite loci
