# Supplementary material for: Population genetics of the Mediterranean corn borer (Sesamia nonagrioides) differs between wild and cultivated plants
Source: PLoS One. 2020 Mar 19;15(3):e0230434. doi: 10.1371/journal.pone.0230434 (PMC7081988; doi:10.1371/journal.pone.0230434)
Supplement: S3 Table — Loc.: Locality, Lo: Longages, Pou: Poucharamet, Cam: Cambernard, Lav: Lavergne, Ar: Arles. Dept.: Department, 07: Ardèche, 13: Bouches du Rhône, 46: Lot, 31: Haute Garonne. Reg.: Region. SW: Southwest, RV: Rhone Valley #: Population number. (DOCX) [file pone.0230434.s009.docx]

| **Loc.** | Lo1 | Lo2 | SCR | Lo3 | Lo4 | Pou | Cam | Lav | Aub | Ar2 | Ar3 | Ar4 | Ar6 | Ar7 | Ar8 | Ar9 | Ar12 | Ar12 | Ar13 | Ar14 | Ar15 | Ar16 | Ar16 | Ar17 | Ar18 |
| --- | --- | --- | --- | --- | --- | --- | --- | --- | --- | --- | --- | --- | --- | --- | --- | --- | --- | --- | --- | --- | --- | --- | --- | --- | --- |
| **Dept** | 31 | 31 | 31 | 31 | 31 | 31 | 31 | 46 | 07 | 13 | 13 | 13 | 13 | 13 | 13 | 13 | 13 | 13 | 13 | 13 | 13 | 13 | 13 | 13 | 13 |
| **Reg.** | SW | SW | SW | SW | SW | SW | SW | SW | RV | RV | RV | RV | RV | RV | RV | RV | RV | RV | RV | RV | RV | RV | RV | RV | RV |
| **Plant** | M | M | M | M | M | M | M | M | S | T | T | T | T | T | T | T | S | T | S | T | S | T | S | T | T |
| **#** | 1 | 2 | 3 | 4 | 5 | 6 | 7 | 8 | 9 | 10 | 11 | 12 | 13 | 14 | 15 | 16 | 17 | 18 | 19 | 20 | 21 | 22 | 23 | 24 | 25 |
| 2 | 0.016 |  |  |  |  |  |  |  |  |  |  |  |  |  |  |  |  |  |  |  |  |  |  |  |  |
| 3 | 0.014 | -0.001 |  |  |  |  |  |  |  |  |  |  |  |  |  |  |  |  |  |  |  |  |  |  |  |
| 4 | 0.012 | -0.006 | -0.009 |  |  |  |  |  |  |  |  |  |  |  |  |  |  |  |  |  |  |  |  |  |  |
| 5 | 0.025 | -0.003 | -0.001 | -0.007 |  |  |  |  |  |  |  |  |  |  |  |  |  |  |  |  |  |  |  |  |  |
| 6 | 0.032 | -0.001 | 0.011 | 0.005 | -0.005 |  |  |  |  |  |  |  |  |  |  |  |  |  |  |  |  |  |  |  |  |
| 7 | 0.019 | 0.003 | 0.001 | -0.002 | -0.006 | 0.005 |  |  |  |  |  |  |  |  |  |  |  |  |  |  |  |  |  |  |  |
| 8 | 0.092 | 0.004 | 0.032 | 0.012 | -0.021 | 0.025 | 0.025 |  |  |  |  |  |  |  |  |  |  |  |  |  |  |  |  |  |  |
| 9 | 0.218 | 0.19 | 0.167 | 0.167 | 0.135 | 0.155 | 0.169 | 0.257 |  |  |  |  |  |  |  |  |  |  |  |  |  |  |  |  |  |
| 10 | 0.132 | 0.106 | 0.102 | 0.102 | 0.089 | 0.079 | 0.105 | 0.236 | 0.239 |  |  |  |  |  |  |  |  |  |  |  |  |  |  |  |  |
| 11 | 0.121 | 0.081 | 0.1 | 0.075 | 0.065 | 0.06 | 0.09 | 0.102 | 0.195 | 0.079 |  |  |  |  |  |  |  |  |  |  |  |  |  |  |  |
| 12 | 0.12 | 0.108 | 0.102 | 0.108 | 0.106 | 0.122 | 0.121 | 0.207 | 0.276 | 0.137 | 0.187 |  |  |  |  |  |  |  |  |  |  |  |  |  |  |
| 13 | 0.215 | 0.177 | 0.191 | 0.142 | 0.168 | 0.176 | 0.179 | 0.043 | 0.455 | 0.387 | 0.248 | 0.355 |  |  |  |  |  |  |  |  |  |  |  |  |  |
| 14 | 0.072 | 0.042 | 0.054 | 0.042 | 0.035 | 0.037 | 0.046 | 0.046 | 0.216 | 0.12 | 0.087 | 0.139 | 0.164 |  |  |  |  |  |  |  |  |  |  |  |  |
| 15 | 0.126 | 0.088 | 0.093 | 0.073 | 0.074 | 0.083 | 0.062 | 0.09 | 0.255 | 0.188 | 0.116 | 0.19 | 0.181 | 0.061 |  |  |  |  |  |  |  |  |  |  |  |
| 16 | -0.073 | -0.079 | -0.058 | -0.083 | -0.092 | -0.014 | -0.064 | -0.122 | 0.314 | 0.172 | 0.02 | 0.148 | _ | -0.018 | 0.12 |  |  |  |  |  |  |  |  |  |  |
| 17 | 0.043 | 0.045 | 0.037 | 0.029 | 0.033 | 0.035 | 0.027 | 0.091 | 0.174 | 0.048 | 0.056 | 0.097 | 0.166 | 0.041 | 0.062 | -0.017 |  |  |  |  |  |  |  |  |  |
| 18 | 0.042 | 0.026 | 0.03 | 0.022 | 0.022 | 0.019 | 0.027 | 0.027 | 0.156 | 0.053 | 0.037 | 0.117 | 0.151 | 0.044 | 0.068 | -0.111 | 0.022 |  |  |  |  |  |  |  |  |
| 19 | 0.132 | 0.046 | 0.073 | 0.045 | 0.02 | 0.029 | 0.048 | -0.058 | 0.3 | 0.169 | 0.029 | 0.181 | 0.192 | 0.057 | 0.062 | 0.035 | 0.031 | 0.011 |  |  |  |  |  |  |  |
| 20 | 0.116 | 0.106 | 0.099 | 0.103 | 0.097 | 0.103 | 0.1 | 0.149 | 0.216 | 0.009 | 0.099 | 0.171 | 0.285 | 0.134 | 0.2 | -0.086 | 0.074 | 0.045 | 0.098 |  |  |  |  |  |  |
| 21 | 0.077 | 0.054 | 0.065 | 0.048 | 0.035 | 0.048 | 0.058 | 0.016 | 0.147 | 0.08 | 0.038 | 0.137 | 0.141 | 0.04 | 0.085 | -0.039 | 0.036 | 0.038 | 0.03 | 0.095 |  |  |  |  |  |
| 22 | 0.055 | -0.006 | -0.019 | -0.016 | -0.006 | 0.011 | -0.067 | 0.008 | 0.328 | 0.216 | 0.133 | 0.261 | _ | 0.102 | 0.111 | _ | 0.003 | 0.001 | 0.02 | 0 | 0.088 |  |  |  |  |
| 23 | 0.033 | 0.046 | 0.047 | 0.035 | 0.039 | 0.04 | 0.041 | 0.09 | 0.178 | 0.07 | 0.064 | 0.1 | 0.147 | 0.079 | 0.105 | -0.03 | 0.024 | 0.036 | 0.068 | 0.067 | 0.046 | 0.052 |  |  |  |
| 24 | 0.028 | 0.037 | 0.032 | 0.012 | 0.027 | 0.041 | 0.037 | 0.082 | 0.194 | 0.097 | 0.056 | 0.14 | 0.146 | 0.089 | 0.123 | -0.116 | 0.031 | 0.013 | 0.087 | 0.052 | 0.054 | 0.028 | 0.009 |  |  |
| 25 | 0.087 | 0.071 | 0.084 | 0.059 | 0.066 | 0.059 | 0.067 | 0.115 | 0.206 | 0.106 | 0.05 | 0.22 | 0.178 | 0.11 | 0.131 | 0.04 | 0.055 | 0.04 | 0.045 | 0.068 | 0.067 | 0.035 | 0.05 | 0.014 |  |

**S3 Table .** Pairwise Fst using ENA (Weir 1996). **Loc**.: Locality, Lo: Longages, Pou: Poucharamet, Cam: Cambernard, Lav : Lavergne, Ar: Arles. **Dept**.: Department, 07: Ardèche, 13 : Bouches du Rhône, 46 : Lot, 31 : Haute Garonne. **Reg**. : Region. SW : Southwest, RV: Rhone Valley **#**: Population number (refers to Fig 1.).
